# Convergence of Soft Electronics and Artificial Intelligence: From Materials to Intelligent Systems

**DOI:** 10.1007/s40820-026-02265-x

**Published:** 2026-06-30

**Authors:** Huijae Park, Gyuho Choi, Sangjin Yoon, Daeho Lee, Seung Hwan Ko

**Affiliations:** 1 Wearable Soft Electronics Lab, Department of Mechanical Engineering, Seoul National University, 1, Gwanak-ro, Gwanak-gu, Seoul, 08826 Republic of Korea; 2 Department of Mechanical Engineering, Gachon University, Seongnam, 13120 Republic of Korea; 3 Institute of Engineering Research/Institute of Advanced Machinery and Design (SNU-IAMD), Seoul National University, 1, Gwanak-ro, Gwanak-gu, Seoul, 08826 Republic of Korea; 4 Interdisciplinary Program in Bioengineering, Seoul National University, 1, Gwanak-ro, Gwanak-gu, Seoul, 08826 Republic of Korea

**Keywords:** Soft electronics, Wearable sensors, Functional materials, Artificial intelligence, E-skin

## Abstract

Soft electronics are an emerging class of mechanically compliant platforms that enable conformal, skin-interfaced sensing and actuation on curvilinear and dynamic surfaces. These systems combine deformation-tolerant electrical functionality with soft contact mechanics, but their in-use performance is strongly influenced by time-varying interfaces, motion-induced artifacts, and the system burden associated with dense multimodal integration. Advances in soft electronics are now converging with artificial intelligence, which supports reliable information extraction from high-dimensional signals and enables on-device inference that tolerates variability across users and day-to-day conditions. Here, progress in this convergence from materials to intelligent systems is summarized. Material and interface foundations are introduced first, focusing on deformation-tolerant conductors, low-impedance biointerfaces, and breathable substrate strategies that support extended wear. Manufacturing and integration approaches are then discussed, highlighting scalable fabrication, multilayer interconnects, and energy-autonomous wireless operation that enable higher channel counts and multifunctional architectures. Learning-based pipelines are subsequently reviewed with emphasis on artifact suppression, nonideality compensation, multimodal inference, and efficient edge deployment. Finally, emerging directions including neuromorphic computing and in-sensor computing are discussed, together with current challenges and future opportunities toward deployable intelligent soft systems that operate continuously and reliably in everyday settings.

## Highlights

- The synergistic integration of advanced soft materials, scalable manufacturing, and hardware architectures is examined to ensure high-fidelity signal acquisition in dynamic environments.
- Recent advances in artificial intelligence and neuromorphic computing that are utilized to overcome the physical limitations of soft materials and enable efficient data processing.
- A detailed discussion on the system-level applications of AI-integrated soft electronics across personalized healthcare, immersive human-machine interfaces, and soft robotics, as well as current deployment challenges and future research directions.

## Graphical abstract

**Figure:**
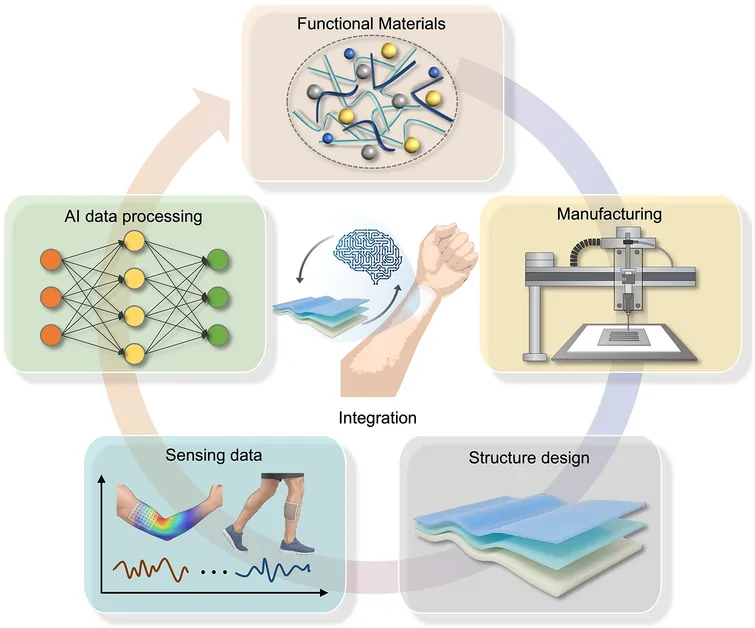

## Introduction

Soft electronics have expanded wearable and skin-interfaced technologies by enabling intimate contact, mechanical compliance, and distributed sensing on surfaces that bend, stretch, and twist. As soft material platforms mature alongside low-power embedded electronics, the field is moving beyond proof-of-concept demonstrations toward sustained operation during everyday use. In this regime, performance is determined not only by baseline sensitivity but also by signal stability under motion, sweat, temperature fluctuations, and repeated deformation. These constraints motivate a convergence perspective in which materials, integration, hardware, and artificial intelligence (AI) are co-designed rather than optimized in isolation.

At the materials level, reliable operation has focused on preserving electrical functionality under deformation while maintaining skin-compatible interfaces. A widely used approach is the use of composite architectures, where conductive fillers form percolation networks inside soft matrices, remain a widely used strategy to retain conduction without sacrificing elastomeric compliance [1–3]. More recently, intrinsically stretchable conductors and conductive polymers have been engineered to sustain continuous conduction pathways at large strain and to support conformal biointerfaces [4, 5]. For biopotential acquisition, self-adhesive and sweat-tolerant PEDOT:PSS electrodes reduce dependence on gels and improve wearability under humid conditions [6, 7]. Concurrently, MXene-based and conducting polymer hydrogels provide mechanically compliant interfaces that lower skin–electrode impedance and boost signal-to-noise ratios [8, 9]. Furthermore, piezoelectric and triboelectric materials enable dynamic motion sensing and self-powered operation [10, 11], while breathable substrates mitigate moisture and heat accumulation during prolonged wear [12, 13].

As systems progress from isolated sensors to integrated wearable platforms, system architecture becomes as decisive as material choice. Scalable manufacturing and dense routing strategies have been introduced to support rising channel counts and multifunctional stacks, including roll-to-roll production and multilayer interconnect approaches that preserve compliance while increasing integration density [14–16]. Additionally, energy-autonomous wireless operation is being realized through motion-powered and thermoelectric architectures, reducing reliance on rigid batteries for continuous monitoring [17, 18]. However, scaling and integration amplify the analysis burden of soft systems. As channel count grows and sensing becomes multimodal, the system produces high-dimensional, time-varying signals whose statistics shift with placement, motion, and environment, making reliable interpretation a core design requirement.

To address this complexity, AI is increasingly integrated not merely as a post-processing tool, but as a core computational layer that bridges physical transduction imperfections and system-level decision-making. End-to-end denoising and motion-tolerant learning pipelines reduce artifact sensitivity during movement [19, 20]. Meanwhile, drift-aware feature learning compensates for longer-term nonidealities such as baseline evolution, reducing the recalibration burden across different users and days of wear [21, 22]. As intelligent systems shift toward edge, lightweight temporal models combined with stretchable sensing represent a critical move toward efficient, closed-loop intelligence on compliant hardware [23, 24].

**Fig. 1:**
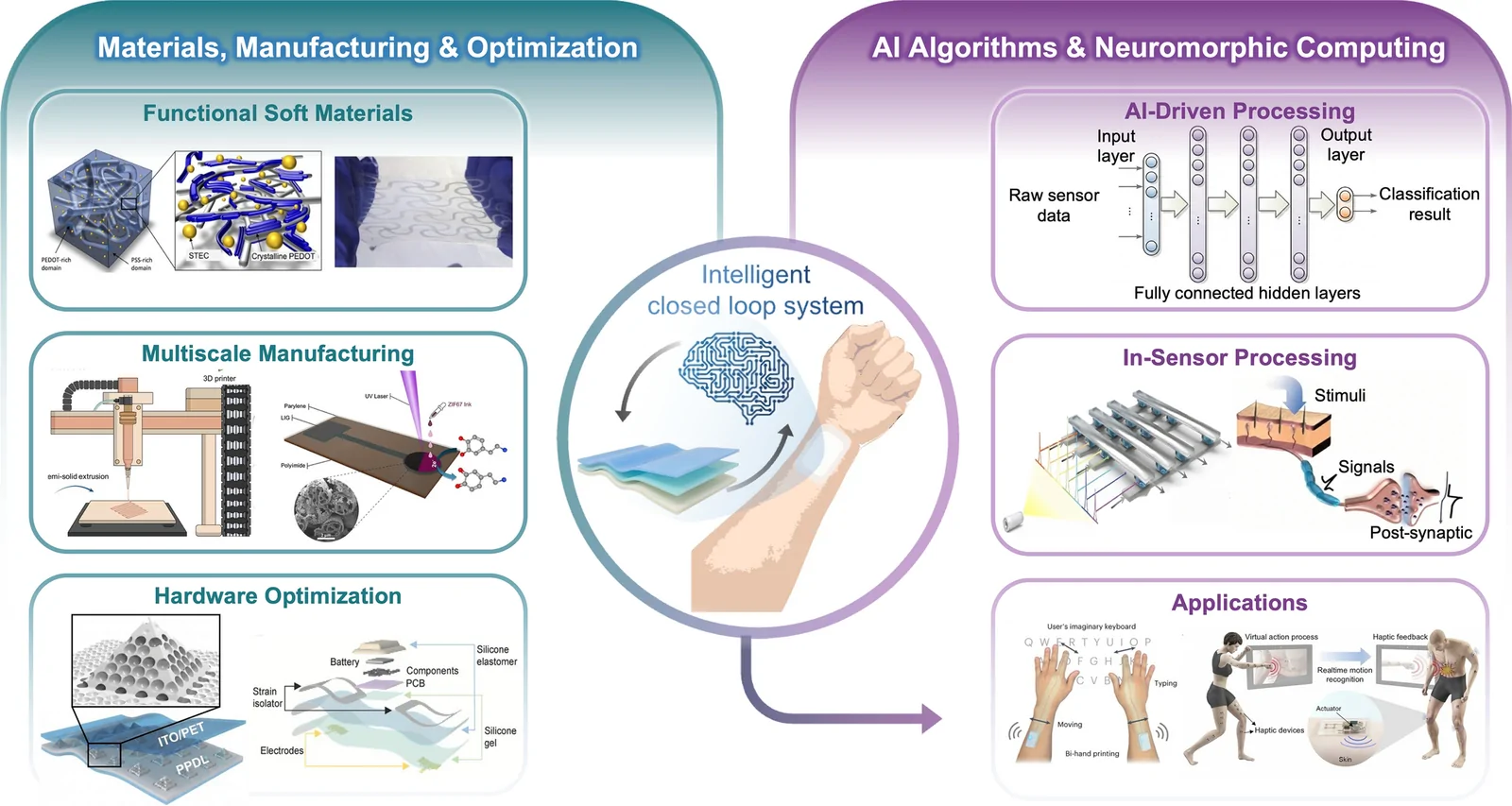
Overview of the synergistic integration of functional soft materials and artificial intelligence for next-generation intelligent soft systems. The schematic highlights two complementary pillars for next-generation intelligent soft systems. The left side focuses on materials, manufacturing, and hardware optimization to generate high-fidelity sensing signals, while the right side presents AI algorithms and neuromorphic in-sensor processing that enable real-time enhancement, inference, and application-level decision-making. The center illustrates an intelligent closed-loop paradigm in which material–device–algorithm co-optimization provides continuous feedback to improve performance in wearable electronics, human–machine interfaces, and soft robotics. Reproduced with permission [4]. Copyright 2017, American Association for the Advancement of Science (AAAS). Reproduced with permission [25]. Copyright 2023, American Association for the Advancement of Science (AAAS). Reproduced with permission [26]. Copyright 2025, American Chemical Society. Reproduced with permission [27]. Copyright 2019, American Chemical Society. Reproduced with permission [28]. Copyright 2021, Wiley–VCH. Reproduced with permission [29]. Copyright 2024, Springer Nature. Reproduced with permission [30]. Copyright 2025, Springer Nature. Reproduced with permission [31]. Copyright 2023, Springer Nature. Reproduced with permission [32]. Copyright 2023, Springer Nature. Reproduced with permission [33]. Copyright 2025, Springer Nature

Several reviews have addressed soft wearable materials, flexible sensing modalities, or learning-based analytics from their respective perspectives. As shown in Fig. 1, this review connects those discussions along a unified path from physical transduction to deployable system-level intelligence, with attention to how materials, integration, power, and learning mutually influence one another. We emphasize cross-layer dependencies that motivate co-design, and we discuss emerging directions, including neuromorphic and in-sensor computing, that may further shorten the sensing-to-decision loop for next-generation intelligent soft systems.

Mechanistically, this co-design problem operates as a cascade of coupled constraints. Material properties like interfacial contact and electromechanical stability dictate device-level signal quality, including the noise floor and baseline drift. Device architecture determines sampling density, which in turn amplifies routing and power requirements. Ultimately, these hardware realities define the algorithmic requirements. Motion-sensitive interfaces demand robust denoising, viscoelastic responses require extended memory, and dense arrays need energy-efficient models. Therefore, cross-layer optimization is best approached as a joint allocation of information, robustness, and power budgets rather than the independent enhancement of isolated components [8, 9, 13–21, 23, 24, 34].

To move beyond a catalog of recent advances, this review identifies the principal deployment bottlenecks currently limiting real-world intelligent soft systems. These include energy-compute trade-offs, long-term stability, recalibration burden, manufacturing variability, and the pressing need for standardized evaluation frameworks.

## Advanced Material Foundations for Intelligent Wearable Systems

Wearable devices often fail in real-world applications not because they lack a sensing mechanism, but because their electrical function, interfacial contact, or mechanical stability deteriorate during repeated operation [35, 36]. Our discussion of materials begins with three practical requirements: preserving reliable electrical performance under deformation, adapting to environmental changes, and sustaining breathable, skin-compatible contact over extended wear [4, 12, 37]. We address these challenges through three broad material directions: electrically active conductors and semiconductors, stimuli-responsive functional materials, and breathable substrates for skin interfacing. Because intelligent wearables must also convert dynamic mechanical inputs into meaningful electrical signals, this chapter additionally considers electromechanical transduction materials, particularly piezoelectric and triboelectric platforms, as part of the broader material foundation for dynamic and self-powered operation [11, 38]. This section therefore defines the material and interfacial basis on which the later discussions of fabrication, system integration, and intelligence are built.

### Conductive, Semiconducting, and Electromechanical Transduction Materials for Soft and Stretchable Electronics

Stable biosignal acquisition during daily activity remains challenging because mechanical deformation and time-varying skin contact continuously disrupt the electrical interface. Accordingly, material selection for wearable electronics is guided not only by how reliably electrical pathways are preserved under deformation, but also by how effectively mechanical inputs can be converted into usable electrical signals under practical wearing conditions [38]. In this context, conductive and semiconducting materials provide the basic electrical framework for signal transport, amplification, and interfacial stability, whereas electromechanical transduction materials directly translate deformation, pressure, or motion into measurable electrical output [10, 11]. Carbon-based nanostructures such as carbon nanotubes and graphene form percolation networks that provide mechanically resilient conduction, while metallic nanomaterials such as silver nanowires and deformable liquid metals deliver low resistivity for interconnects and high-current operation. In parallel, conducting polymers such as PEDOT:PSS and emerging two-dimensional materials such as MXenes support bioelectronic interfaces through mixed ionic-electronic transport, hydrophilic surfaces, and high effective surface area [1, 2, 4, 39]. Beyond these conductive pathways, piezoelectric and triboelectric materials broaden the material basis of soft electronics by enabling dynamic sensing and, in some cases, self-powered operation [11, 38]. From this standpoint, the following discussion considers four complementary material directions: carbon-based nanostructures, metallic conductors and liquid metals, conducting polymers and MXene-based materials, and electromechanical transduction materials for dynamic and self-powered sensing.

#### Carbon-based Nanostructures–Carbon Nanotubes and Graphene

Carbon nanotubes (CNTs) are widely used as conductive fillers for soft electronics because their high aspect ratio supports percolated transport networks within diverse matrices while tolerating mechanical deformation. CNT networks have been implemented across wearable-friendly substrates including fabrics and smart textiles, paper-based platforms, and porous foams, enabling conformal sensing under repeated deformation and daily-use conditions [40–43]. In practice, device performance is often governed by network morphology and junction behavior rather than the intrinsic conductivity of individual nanotubes, so architectural control of alignment and bundle patterning has been used to tune strain response under deformation [44]. Zhu et al. reported electronic skins that employ aligned few-walled CNT arrays embedded in polymer composites, where deformation modulated anisotropic conduction pathways and intertube contact resistance and the devices maintained stable responses after 15,000 bend–release cycles at power levels below 10 µW [1].

**Fig. 2:**
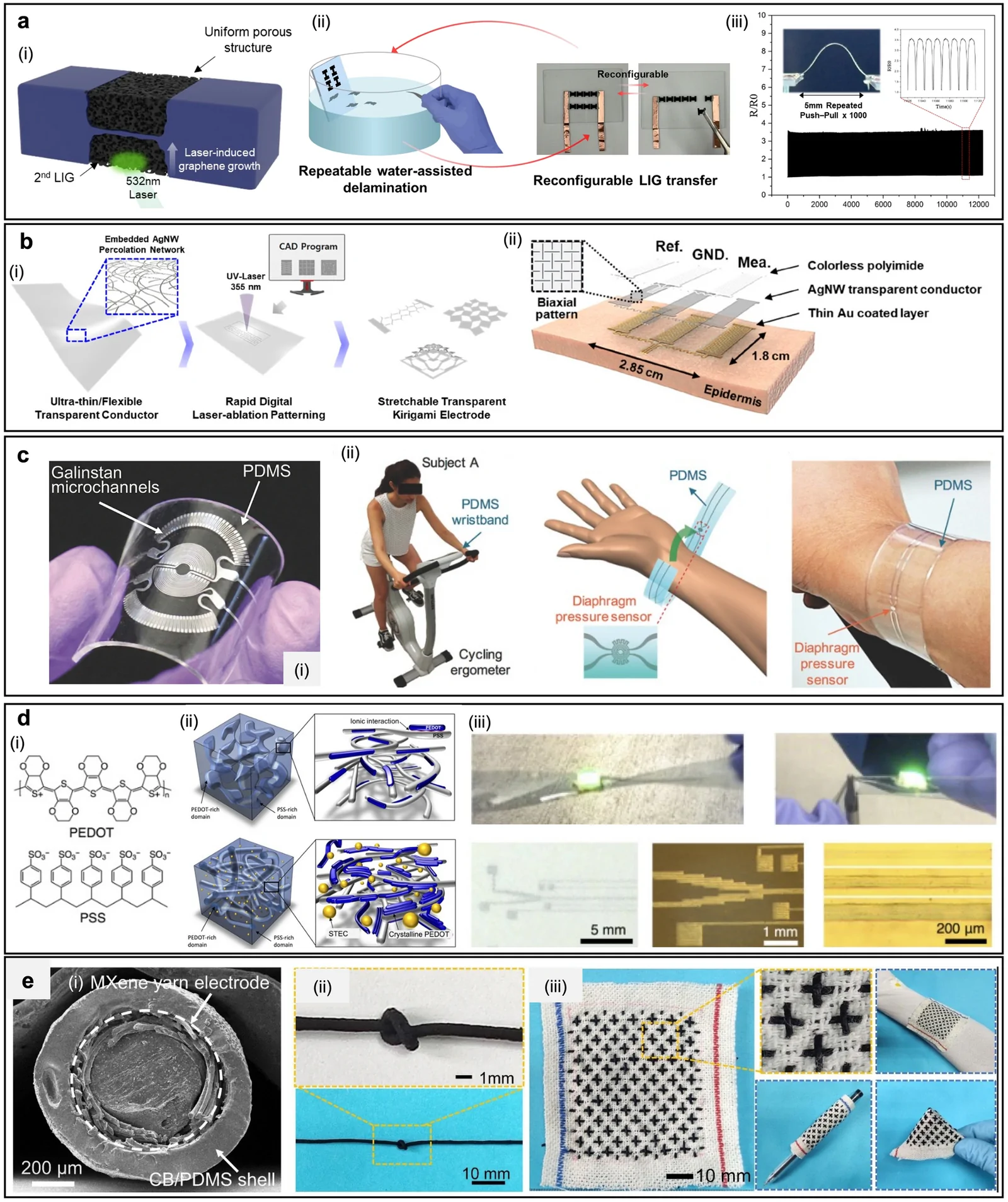
Conductive and semiconducting materials for soft and stretchable electronics. **a** Process schematic illustration for laser-induced graphene, transfer application, and bending-cycle durability test. Reproduced with permission [48]. Copyright 2025, Elsevier. **b **Laser-patterned Ag nanowire percolation electrodes in kirigami layouts for stretchability, including a kirigami EP sensor for capturing electrophysiology signals. Reproduced with permission [2]. Copyright 2019, American Chemical Society. **c** PDMS microfluidic diaphragm pressure sensor using Galinstan channels for wearable monitoring, including a photograph of the fabricated device. Reproduced with permission [49]. Copyright 2017, Wiley–VCH. **d** Morphology schematics comparing a conventional PEDOT:PSS film and a STEC-enhanced stretchable PEDOT film, with additive-engineered stretchable PEDOT:PSS conductors. Reproduced with permission [4]. Copyright 2017, American Association for the Advancement of Science (AAAS). **e** Elastomer-encapsulated MXene-based yarn electrodes for fiber and textile devices. Reproduced with permission [39]. Copyright 2024, Elsevier

Graphene offers complementary advantages through high carrier mobility and accessible surface area, and wearable implementations frequently employ porous frameworks or controlled microcrack networks to amplify resistance modulation for motion and physiological-signal detection [45, 46]. Laser-induced graphene (LIG) provides a practical route to patterned graphene-like conductors on polymeric precursors, and wearable demonstrations have leveraged crack-engineered designs [47]. Choi et al. reported robust freestanding LIG electrodes formed by double-sided laser irradiation of a PEDOT:PSS–Kevlar composite film, which reduced ablation and promoted more uniform through-thickness porosity, retained low resistance after 1000 bending cycles, and showed minimal change before and after adhesive-free transfer, enabling reattachment and circuit rearrangement on diverse substrates for wearable energy devices [48] (Fig. 2a). Overall, carbon-based nanostructures are best interpreted through a structure–property–integration perspective, where network topology, junction stability, and transferability collectively determine on-body signal reliability and multifunctional device integration.

#### Metallic Nanomaterials and Deformable Liquid Metals

Metallic nanomaterials, particularly silver nanowires (AgNWs), are essential components for developing transparent and stretchable electrodes due to their high electrical conductivity and optical translucency. These one-dimensional (1D) nanostructures form percolation networks that maintain electrical pathways under moderate deformation, making them suitable for precise biosignal sensing and sensor arrays. Recent advancements have demonstrated that integrating AgNW percolation networks into Kirigami-inspired architectures facilitates extreme mechanical stretchability while maintaining high transparency. This structural approach allows the conductive network to withstand large-scale strains by redistributing mechanical stress through engineered cuts, preventing the abrupt failure of electrical connections. Won et al. introduced a kirigami-patterned nanowire percolation electrode with application-tunable elasticity, achieving ultrahigh stretchability beyond 400% tensile strain while largely preserving electrical characteristics, and maintaining strain reversibility over 10,000 stretch–release cycles with optical transparency above 80% [2] (Fig. 2b). They further demonstrated wearable-relevant implementations including an ultrastretchable transparent heater and a conformal electrophysiology sensor, and extended the platform toward human–machine interfacing by integrating the e-skin sensor with a quadrotor drone.

Beyond kirigami architectures, aligned AgNW frameworks have also enabled ultrasensitive monitoring of subtle human motion, providing a complementary example of how nanowire orientation and network geometry can improve strain-sensing performance [50].

Liquid metals (LMs), such as gallium-based alloys, offer a unique combination of metallic conductivity and fluidic deformability, enabling intrinsically stretchable conductors and interconnects that can accommodate large strains without the fatigue typical of solid metals. Unlike solid conductors, LMs remain in a liquid state at room temperature, allowing them to flow and reconfigure within microfluidic channels or elastomeric matrices under deformation. In wearable implementations, performance and reliability are closely tied to oxide-mediated interfacial behavior, robust encapsulation against leakage, and stable electrical contact under repeated bending and stretching. These materials support high-performance sensing platforms, such as microfluidic diaphragm pressure sensors for health and tactile monitoring, which leverage the fluidic nature of LMs to detect subtle pressure changes through geometric deformation. Gao et al. demonstrated a microfluidic tactile diaphragm pressure sensor based on embedded Galinstan microchannels with a 70× 70 µm cross-section, achieving sub-50 Pa pressure resolution with detection limits below 100 Pa and a response time of 90 ms. They further implemented an embedded equivalent Wheatstone-bridge configuration that increased the voltage sensitivity to 0.0835 kPa^−1^ and enabled temperature self-compensation over 20–50 °C [49] (Fig. 2c). Wearable demonstrations included a PDMS wristband for real-time pulse monitoring and a multi-sensor PDMS glove for distributed tactile feedback. High-resolution LM patterning on hydrogel interfaces further supports skin-conformal layouts and self-recovering electrical pathways under damage [51].

#### Conducting Polymers and Emerging 2D Materials (MXenes)

Conducting polymers, represented by poly(3,4-ethylenedioxythiophene):poly(styrenesulfonate) (PEDOT:PSS), are central to wearable bioelectronic interfaces because mixed ionic–electronic transport can reduce interfacial impedance and stabilize on-skin recordings under motion. Beyond geometric strain engineering, recent progress has progressively targeted intrinsically stretchable molecular conductors that preserve high conductivity under large deformation. Wang et al. reported an intrinsically stretchable PEDOT:PSS platform in which ionic “enhancer” additives both promote morphology reorganization and increase doping efficiency. Under large deformation, the films still exceeded 100 S cm^−1^ at 600% strain, with fracture occurring near 800%. In cyclic loading, conductivity remained ~ 3600 S cm^−1^ after 1000 cycles to 100% strain. Under static strain conditions, the system delivered > 4100 S cm^−1^ at 100% strain and > 3100 S cm^−1^ at 0% strain [4] (Fig. 2d).

Biointerface performance has also been improved through molecular and mesoscale structuring, including topological supramolecular network designs and vertical phase-separation strategies that promote robust charge transport and stable contact for electrophysiology sensing [5, 52]. For long-term wear, self-adhesive and sweat-tolerant PEDOT:PSS dry electrodes have been developed to support conformal ECG/EMG acquisition without auxiliary fixation, emphasizing stable adhesion and environmental robustness as practical determinants of signal quality [6, 7]. In parallel, binder-free vapor-phase deposition on everyday fabrics expands process compatibility for breathable, textile-integrated PEDOT:PSS sensors [53].

MXenes, particularly Ti_3_C_2_T_*x*_, provide a rapidly expanding materials platform for wearable sensing due to metallic conductivity and hydrophilic, functionalized surfaces that integrate well with soft matrices and fibers [54–56]. Mechanical sensing has been advanced through composite and hierarchical architectures, including tile-like stacked microstructures for broad-range strain sensing, electrostatically assembled MXene/CNT hybrids for waterproof pressure sensing in e-skin formats [57, 58]. Yu et al. developed a stretchable multimodal textile sensor using a core–sheath coaxial yarn in which a Ti_3_C_2_T_*x*_-coated elastic yarn serves as the core electrode and a carbon black/PDMS layer functions as the piezoresistive sheath; the multilevel structure supported pressure sensitivity up to 16.21 N^−1^ over a 5 N range and a gauge factor of 12.09 over 100% strain with good cyclic stability, while multi-electrode acquisition and stimulus-dependent signal polarity allowed a single yarn platform to differentiate pressure, strain, and bending [39] **(**Fig. 2e**)**. Collectively, MXene-supported fibers, papers, and printed devices support multimodal, high-density sensing layers that can be embedded into wearable form factors while maintaining soft, body-compatible integration [54, 55, 59].

Collectively, carbon nanomaterials, metallic conductors including deformable liquid metals, and conducting polymers with emerging MXene-based 2D conductors each define distinct materials platforms for wearable sensing, with device-level performance governed not only by intrinsic electrical properties but also by the adopted form factor and integration strategy.

#### Electromechanical Transduction Materials for Dynamic and Self-Powered Wearable Sensing

Conductive fillers and mixed ionic-electronic materials provide the basic electrical framework for wearable biointerfaces, but they do not fully explain how intelligent wearables read dynamic events on the body. Signals such as pulse waves, transient touch, and body motion originate from mechanical deformation, so materials that directly convert deformation into electrical output are also needed [38, 60]. In this respect, piezoelectric and triboelectric materials extend the material foundation of intelligent wearables by introducing transduction modes that are suited to dynamic sensing and, in some cases, power generation [61].

**Fig. 3:**
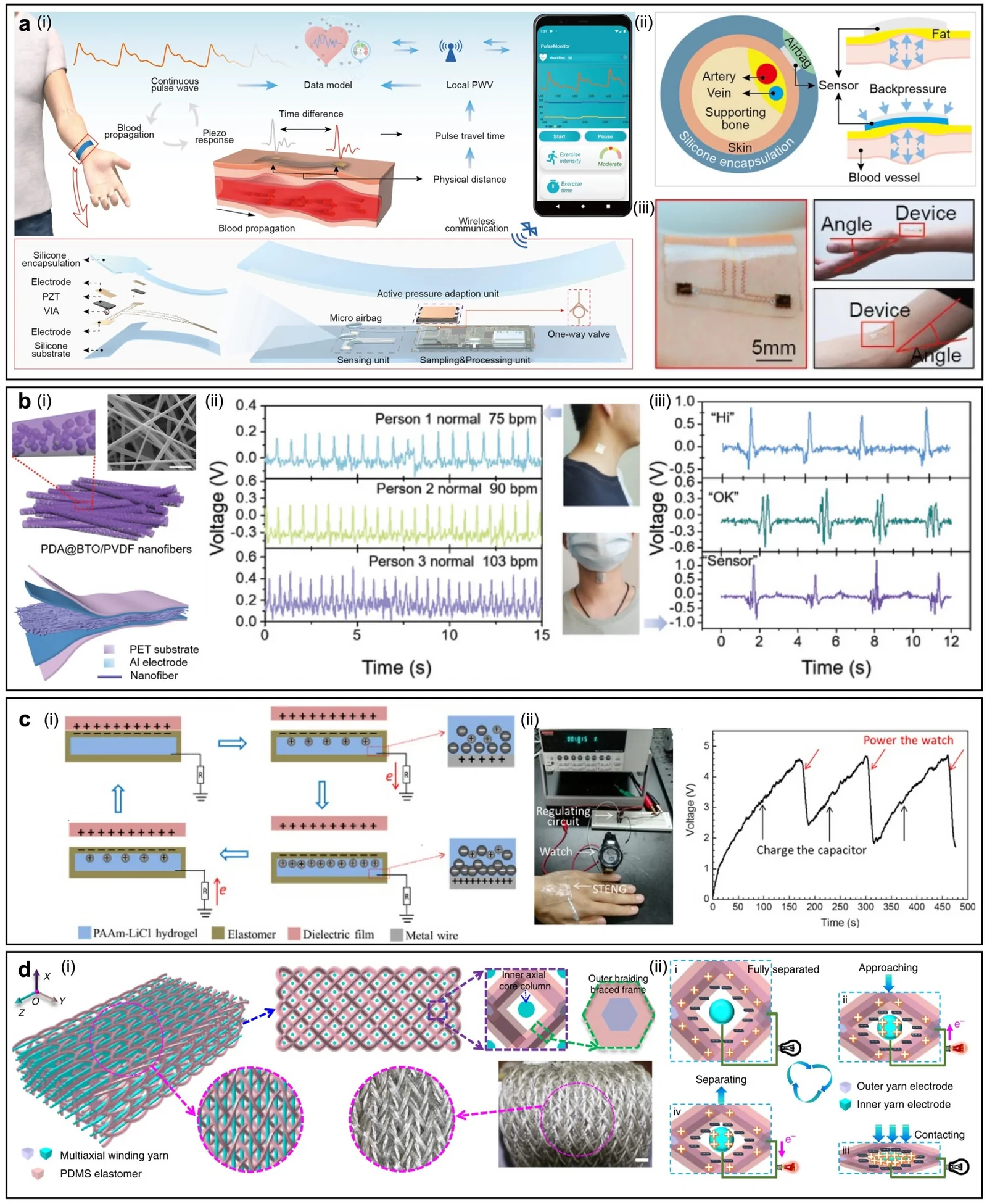
Piezoelectric and Triboelectric Materials and Systems Beyond Purely Conductive Pathways. **a** Wireless piezoelectric wristband for continuous blood-pressure monitoring, showing local pulse-wave sensing, airbag-assisted backpressure control, and mounted-device images under wrist and elbow deformation. Reproduced with permission [38]. Copyright 2023, Springer Nature. **b** Dopamine-coated electrospun nanofibers for multimodal physiological monitoring, showing fiber morphology, pulse waveforms from different users wearing the MFP textile on the neck, and dynamic output signals for spontaneous voice recognition. Reproduced with permission [60]. Copyright 2021, Wiley–VCH. **c** Working mechanism of the STENG and its self-charging operation, showing the energy-harvesting principle, a self-charging system powering an electronic watch, and the voltage profile of a 2.2 µF capacitor during charging and watch operation. Reproduced with permission [11]. Copyright 2017, American Association for the Advancement of Science (AAAS). **d** Structural design and operating principle of the 3DB-TENG, showing the braided architecture with an outer braced frame and inner axial core, the 3D four-step rectangular braiding strategy, and the vertical contact-separation working mechanism. Reproduced with permission [62]. Copyright 2020, Springer Nature

This role is well illustrated by piezoelectric systems for physiological monitoring. Li et al. developed a wrist-mounted platform that combined a piezoelectric sensor array with contact control, signal acquisition, and learning-based estimation for continuous blood-pressure tracking. A key point of this work was not only signal generation itself, but also stable coupling to the artery during wear. With this design, the system reached Grade A performance, with errors of − 0.05 ± 4.61 mmHg for systolic pressure and 0.11 ± 3.68 mmHg for diastolic pressure, and was evaluated in 87 volunteers [38] (Fig. 3a).

Piezoelectric transduction is also useful when the sensing layer must remain effective in textile-like formats under repeated deformation. Su et al. reported a nonwoven piezoelectric textile in which interfacial design improved both mechanical integrity and signal output. The device showed a sensitivity of 3.95 V N^−1^ and less than 3% signal loss after 7400 cycles, indicating that piezoelectric sensing can be translated from discrete soft devices to wearable textiles for pulse monitoring, motion sensing, and voice-related detection [60] (Fig. 3b).

Other studies broaden this piezoelectric direction further. Recent work has moved toward artificial mechanoreceptive behavior and AI-enabled piezoelectric wearable monitoring, suggesting that piezoelectric materials can support not only signal capture but also softer and more integrated device concepts [10, 63, 64].

Triboelectric materials address a somewhat different need. Their importance becomes clearer when sensing, deformability, and reduced dependence on external power are required at the same time [65]. Pu et al. showed this with a transparent and stretchable triboelectric interface that functioned both as an energy harvester and as a pressure-sensitive skin. The device sustained 1160% uniaxial strain, maintained 96.2% average transmittance in the visible range, and delivered a peak power density of 35 mW m^−2^, showing that a single soft structure can support both tactile readout and self-powered operation [11] (Fig. 3c).

The same idea becomes more practical when implemented in textile architectures. Dong et al. designed a three-dimensional braided triboelectric textile in which the fabric structure itself created the contact-separation geometry needed for output generation. Under 3 Hz loading and 20 N force, the device produced an open-circuit voltage of 90 V and a peak power density of 26 W m^−3^, and it could resolve weight changes below 0.1 g [62] (Fig. 3d). This result is meaningful because it shows that textile structure can shape transduction behavior directly, rather than simply serving as a carrier.

These studies clarify the distinct roles of piezoelectric and triboelectric materials in wearable systems [64, 66]. Piezoelectric platforms are effective for converting small biomechanical inputs into stable electrical signals, whereas triboelectric platforms are particularly useful when self-powered sensing, tactile interaction, and textile-compatible energy harvesting are required at the same time. Considering both therefore extends the material discussion beyond maintaining conductivity under strain to selecting transduction routes for dynamic and multifunctional wearable operation [65, 66].

These distinctions suggest that material selection in intelligent wearable systems depends not only on conductivity, but also on how each material class balances deformability, interface stability, and transduction function for the intended application. Carbon nanomaterials are useful for mechanically resilient conductive networks, although their performance can depend strongly on network morphology and junction stability. AgNWs are attractive for transparent and highly conductive electrodes, whereas liquid metals are more suitable for large-strain interconnects and deformable current pathways, despite the need for encapsulation and leakage control. Conducting polymers such as PEDOT:PSS are effective at soft biointerfaces because their mixed ionic-electronic transport can reduce interfacial impedance, while MXenes are particularly attractive for multimodal and high-surface-area sensing because their hydrophilic and functionalized surfaces support printing, fiber integration, and chemical interfacing, despite concerns regarding oxidation and long-term stability. In contrast, piezoelectric and triboelectric materials extend the material framework beyond conductive pathways by directly converting mechanical inputs into electrical output. Piezoelectric materials are generally better suited for stable readout of subtle biomechanical signals, whereas triboelectric materials are more attractive for self-powered sensing, tactile interaction, and textile-compatible energy harvesting. Table 1 further summarizes representative examples across these material classes by comparing their device forms, key performance metrics, and wearable applications.

**Table 1:** Material platforms and key metrics for wearable sensors

Category	Material	Material form	Representative performance	Application demonstration	References
Carbon nanomaterials	Carbon nanotube	SWNT–tissue-paper stacked pressure patch	Sensitivity 2.2 kPa^−1^ (35–2500 Pa) Sensitivity 1.3 kPa^−1^ (2500–11,700 Pa)	Radial pulse monitoring Muscle motion monitoring	[41]
		Zigzag CNT-bundle array strain strip	GF 0.51–64.08 Strain range up to 500%	Subtle motion monitoring Large motion monitoring	[44]
	Graphene	GWF-on-tape strain patch	GF ≈ 10^3^ (2–6% strain) GF ≈ 10^6^ (> 7% strain)	Phonation monitoring Pulse monitoring	[45]
		Graphene-paper piezoresistive pressure patch	Working range 0–20 kPa Sensitivity 17.2 kPa^−1^ (0–2 kPa)	Respiration monitoring Voice recognition	[46]
		Crack-engineered LIG-on-PI-fabric strain strip	*R*^2^ = 0.99747 (0–10% strain) GF ≈ 17 (0–10% strain) GF = 420 (0–2% strain, crack-engraved)	Breathing monitoring Speech-signal recognition	[47]
Metallic conductors and liquid–metal systems	Nanowire	Kirigami AgNW transparent electrode	Optical transparency > 80% Tensile strain > 400% 10,000-cycle reversibility	Electrophysiology monitoring Heater actuation	[2]
		Aligned nanowire strain strip	GF ≈ 35.8 Response ≈ 230 ms Hysteresis < 8.1% 1000 cycles; power < 35 µW	Finger motion monitoring Swallowing monitoring	[50]
	Liquid metal	Galinstan microchannel diaphragm pressure sensor	Sub-50 Pa resolution < 100 Pa detection limit Response 90 ms Sensitivity 0.0835 kPa^−1^	Pulse monitoring Tactile-pressure sensing	[49]
		LM-on-hydrogel fine-line patterning	Minimum linewidth 85 µm	Hydrogel interconnect patterning Self-healing prototyping	[51]
Conducting polymers and MXene-based 2D conductors	Conducting polymer	STEC-PEDOT film conductor	> 3100 Scm^−1^ (0% strain) > 4100 Scm^−1^ (100% strain) 3600 Scm^−1^ after 1000 cycles at 100% strain	Interconnect routing Transistor-array integration	[4]
		oCVD-PEDOT-on-fabric pressure sensor	Sensitivity 8 kPa^−1^ Response/recovery 260/30 ms (≈ 1.8 kPa)	Fabric-pressure sensing Wearable monitoring	[53]
	MXene	MXene–PANIF tile-stacked strain strip	GF up to 2369.1 Range up to 80% strain Detection limit 0.1538% strain	Pulse monitoring Wireless transmission	[57]
		Waterproof MXene sponge pressure sensor	Sensitivity 10.8 kPa^−1^ Detection limit 4.6 Pa Response/recovery 40/60 ms 5000 cycles; WCA 142°	Pressure mapping Waterproof monitoring	[58]
		Core–sheath CPMY yarn multimodal textile sensor	Pressure sensitivity 16.21 N^−1^ (range 5 N) Durability 2000 cycles GF 12.09 (0–100% strain)	Multimodal discrimination Gesture recognition	[39]
Electromechanical transduction materials	Piezoelectric materials	PZT-based wrist-mounted piezoelectric sensor array	Grade A BP accuracy Error − 0.05 ± 4.61 mmHg for systolic BP Error 0.11 ± 3.68 mmHg for diastolic BP	Continuous wireless blood-pressure monitoring	[38]
		PDA@BTO/PVDF nanofiber nonwoven piezoelectric textile	Sensitivity 3.95 V N^−1^ < 3% signal decline after 7400 cycles	Pulse monitoring Motion monitoring Voice recognition	[60]
	Triboelectric materials	PDMS-based ultrastretchable transparent triboelectric e-skin	Uniaxial stretchability 1160% Average visible transmittance 96.2% Peak power density 35 mW m^−2^	Biomechanical energy harvesting Tactile sensing	[11]
		PDMS-coated yarn 3D braided triboelectric textile	Open-circuit voltage 90 V at 3 Hz and 20 N Peak power density 26 W m^−3^ Weight resolution < 0.1 g	e-textile power harvesting Self-powered sensing	[62]

### Advanced Functional Materials with Environmental Responsiveness

Beyond providing stable electrical pathways, next-generation wearable systems are progressively incorporating stimuli-responsive materials that can actively adapt to their surrounding environment. In soft electronics, these environmental perturbations span a broad range of conditions, from changes in temperature and local chemical environment to repeated deformation and abrasion at material networks or skin interfaces [67–69]. This shift from passive sensing to “smart” functionality is essential for enhancing the long-term reliability and user interactivity of soft electronics, particularly under daily-use conditions where sweat, temperature fluctuations, repetitive deformation, and abrasion continuously perturb both material networks and skin interfaces. To achieve this, materials are increasingly designed to embed adaptation directly into the material platform rather than relying solely on electronic or algorithmic compensation [70]. In this context, environmental responsiveness in wearable materials should be understood not only as immediate stimulus-driven functional change, but also as a broader adaptive capacity that helps preserve usability, readability, and operational reliability under variable real-world conditions.

From this perspective, representative strategies include reversible physical transformation, intuitive optical signaling, and, under harsher conditions, autonomous recovery [71–73]. Phase change materials allow adaptive mechanical and thermal management by reversibly tuning stiffness, shape, and heat transport, which is particularly valuable for conformal biointerfaces and thermally robust operation. Thermochromic and colorimetric materials add visualized sensing through stimulus-induced color changes, enabling immediate feedback and low-power, human-interpretable readout. When repeated use conditions progress from functional perturbation to actual structural damage such as cracks, delamination, or percolation-network disruption, autonomous self-healing materials supported by dynamic bonding chemistries become important as an extended strategy for restoring structural and functional integrity. Collectively, these environmentally responsive materials transform wearable platforms into resilient and interactive systems that can perform reliably under the complex and variable conditions of real-world use [74].

#### Phase Change Materials for Functional Transformation and Adaptive Switching

**Fig. 4:**
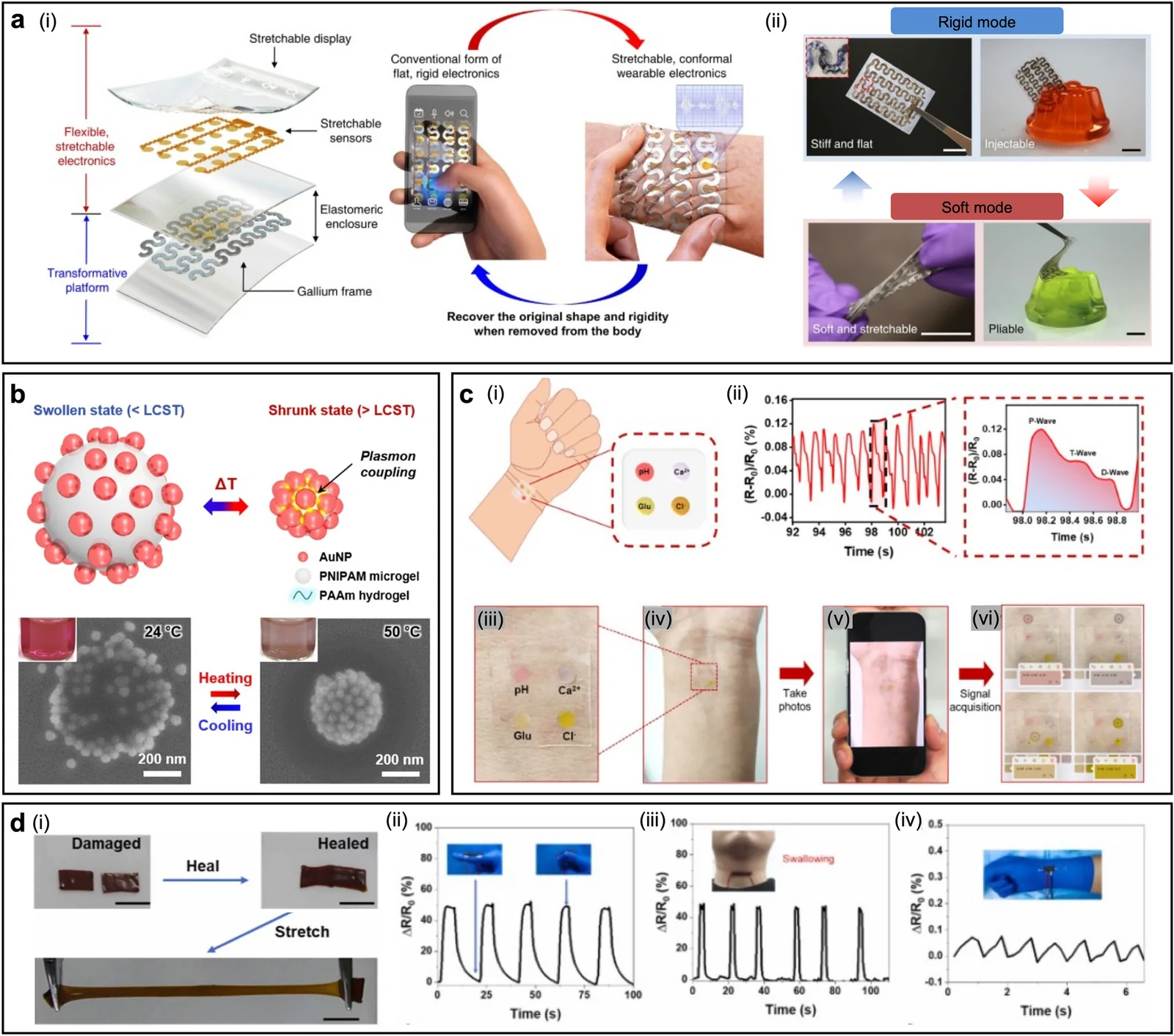
Advanced functional materials with environmental responsiveness for reliable, adaptive, and visualized soft electronics. **a** Temperature-triggered rigid-to-soft transition from a flat handheld device to a skin-mounted wearable for EMG/ECG recording, with photographs of rigid (top) and soft (bottom) modes. Reproduced with permission [71]. Copyright 2019, American Association for the Advancement of Science (AAAS). **b** SEM images of 51 nm AuNP plasmonic microgels in swollen (24 °C) and shrunken (50 °C) states, with inset photos of the dispersions. Reproduced with permission [72]. Copyright 2018, Springer Nature. **c** Wearable hydrogel patch for sweat detection and pulse monitoring, including an on-skin image after sweat secretion and a smartphone app–based colorimetric analysis schematic. Reproduced with permission [68]. Copyright 2025, Elsevier. **d** PGS-0.2DA-0.2PIL self-healing sensor with tensile recovery after 9 h healing at room temperature and on-body monitoring of finger flexion, swallowing, and pulse. Scale bar 500 µm. Reproduced with permission [69]. Copyright 2024, Elsevier

Among environmentally responsive material strategies, phase change materials provide a representative route for adaptive functional switching in wearable systems, where mechanical compliance is not always a fixed requirement. Phase change materials (PCMs) support reconfigurable wearable and implantable systems by providing reversible transitions in physical state and mechanical properties, thereby allowing devices to actively tune stiffness, shape, and stretchability in response to thermal stimuli. This adaptive switching is particularly valuable for intelligent wearables because mechanical compliance is not always a fixed requirement. Devices often benefit from being rigid during handling, alignment, or insertion, while remaining soft and conformal during long-term operation on the body. A representative advance in this direction is temperature-triggered mechanically transformative electronics, where platform-level tuning of mechanical characteristics supports shape- and flexibility-reconfigurable systems without sacrificing functional reliability. In this context, Byun et al. outlined a framework spanning device architectures, materials and underlying physical principles, and scalable fabrication approaches to achieve programmable stiffness while retaining stretchability. Their gallium-based transformative platform exhibited a stiffness tuning ratio greater than 104, with effective elastic moduli ranging from 50 to 950 MPa in the rigid state and decreasing to about 30 kPa in the soft state after phase transition. In addition, their tunable pressure sensor showed an onset sensitivity change from 15.77 to 0.86 kPa^−1^ while expanding the dynamic range from approximately 80 kPa to 1.0 MPa, quantitatively illustrating how PCM-enabled mechanical switching can rebalance sensitivity and operating range depending on device use conditions. In summary, the study illustrates that PCM-enabled mechanical switching can expand the practical design space for both wearable and implantable systems [71] (Fig. 4a).

Alongside mechanical transformation, PCMs contribute to thermal management and environmental adaptation in soft hardware, where heat flow and temperature excursions directly affect both comfort and signal stability during prolonged wear. Highly flexible phase-change films that integrate solar thermal storage with sensitive motion detection exemplify a materials pathway toward wearable thermal regulation while preserving transduction capability under deformation [67]. PCMs can also be combined with thermoresponsive hydrogel interfaces to introduce adaptive optical/electrical switching, as demonstrated by hydrogels that encode rewritable transparency-based dynamic memory behaviors while remaining strain-sensitive, enabling multifunctional interfaces whose readout can be modulated by both temperature and deformation [70]. Collectively, PCM-supported platforms extend environmentally responsive design beyond sensing toward system-level reconfigurability, improving the robustness and usability of wearable bioelectronics under variable real-world conditions.

#### Thermochromic and Colorimetric Materials for Visualized Sensing Indicators

Beyond adaptive switching of mechanical and thermal states, environmentally responsive materials can also provide intuitive visual readouts of external or physiological changes, which is particularly useful in wearable settings where continuous electronic readout is constrained by power, wiring, and motion robustness.

Thermochromic and colorimetric materials address this need by converting temperature, chemistry, or deformation into human-interpretable optical signals that can be read directly or captured using simple imaging tools such as a smartphone camera, enabling low-power and user-friendly sensing layers that complement electrical transduction.

In temperature visualization, material and structural design determine whether color change remains fast, repeatable, and mechanically stable under bending and stretching. Micro/nanoencapsulated phase-change-based thermochromic membranes offer flexible platforms for reversible color switching under thermal cycling, while porous nanofiber architectures further enhance sensitivity by increasing surface area and accelerating thermal exchange [75, 76]. At the device level, these optical layers can be integrated into textile formats to realize dynamic thermochromic smart textiles for temperature-visualized healthcare and thermal management, where spatial color patterns directly report local thermal states [77].

As a representative example of wearable-ready colorimetric temperature sensing, Choe et al. developed a stretchable hydrogel patch embedding thermoresponsive plasmonic microgels based on AuNP-decorated PNIPAM. The patch exhibited rapid, strain-insensitive color switching with an extinction-peak shift of 176 nm within ~ 1 s and a temperature resolution of ~ 0.2 °C. By tuning the transition temperature, they further implemented array-type patches that enable clear visualization across 25–40 °C [72] (Fig. 4b).

Moving beyond thermal readout alone, Wu et al. demonstrated a multimode hydrogel wearable patch that integrates a strain-responsive conductive hydrogel with a colorimetric hydrogel for sweat analysis. The conductive hydrogel exhibited a toughness of 1279.6 kJ m^−3^, conductivity of 3.06 S m^−1^, self-healing efficiency of 95% within 12 h, an ultralow strain detection limit of 0.25%, and fast response and recovery times of 113 and 69 ms, respectively. In parallel, the colorimetric module covered physiologically relevant sweat ranges of pH 4–6.8, Cl^−^ 40–60 mM, Ca^2+^ 0–2 mM, and glucose 0.06–0.2 mM, thereby extending the system from visual signal indication to semi-quantitative physiological assessment. Together, these features enabled simultaneous monitoring of pulse, ECG, breathing, joint motion, and sweat biomarkers through a smartphone-assisted vision interface, while maintaining self-healing, tissue adhesion, transparency, flexibility, and antibacterial behavior [68] (Fig. 4c).

Finally, fiber-level integration strategies provide a scalable route to textile-integrated, multifunctional systems. One representative approach incorporates thermochromic particles into coaxial composite fibers, allowing optical cues and electrical signals to be co-located within wearable form factors [78].

#### Damage-Responsive Self-Healing Materials for Durable Soft Electronics

When wearable materials are subjected to repeated deformation and interfacial stress during prolonged use, environmental responsiveness must extend beyond functional adaptation to include autonomous recovery, which is essential for maintaining the long-term operational stability, structural integrity, and signal reliability of soft electronic components under daily stretching, bending, and twisting [74].

Unlike conventional elastomers that accumulate irreversible cracks and interfacial delamination, self-healing polymers use dynamic crosslinking motifs to restore mechanical cohesion and electrical continuity after structural failure, thereby reducing drift and intermittent signal loss that directly undermine downstream data-driven interpretation. Accordingly, self-healing design in wearables spans multiple scales, from polymer-network strategies that support repeatable recovery across environments, including physically entangled PDMS networks, to bioinspired architectures that combine softness with toughness for stable cyclic electromechanical responses, and to multilayer-oriented approaches that autonomously realign damaged stacks to recover interconnect function while mitigating electrical crosstalk [79, 80].

A recent advance that is particularly relevant to bioelectronic interfacing is the emergence of hydrogel-like conductive elastomers that integrate skin-matching softness with elastomer-like durability and environmental stability within a single platform. Li et al. demonstrated this concept by molecularly designing a hydrogel-like conductive elastomer comprising a furfuryl alcohol-modified PGS prepolymer and a poly(ionic liquid) crosslinked through reversible Diels–Alder chemistry. The elastomer combined a low modulus of 6.41 kPa with ultrafast self-healing, recovering about 98% of both fracture strain and strength within 5 s at room temperature, while also maintaining rapid healing in water and at − 5 °C under slight pressure. It further exhibited stable electrical responses over 3000 cycles at 100% strain, environmental stability over 100 days in air, and a broad sensing range from 0.1% to 500% strain. These attributes enabled durable and recyclable flexible sensors for biocompatible on-body applications including facial motion sensing, ECG/EMG recording, and robotic-arm control **(**Fig. 4d**)**.

Beyond mechanical recovery alone, self-healing concepts are increasingly extended toward multifunctional modules, where damage tolerance must be maintained across self-powered sensing and energy-related components. Representative examples include stretchable conductive eutectogels that combine self-repair with energy harvesting and self-powered sensing [73].

### Conformal Contact and Breathable Substrates for Skin-Interfaced Electronics

The physical coupling between a wearable device and human skin is often the dominant determinant of signal quality, because even high-performance sensing materials can be undermined by imperfect contact, variable pressure, and sweat-induced interfacial changes [81]. Accordingly, substrates for skin-interfaced electronics must deliver conformal contact to stabilize the electrical interface on curvilinear, textured skin, while maintaining high breathability to prevent sweat accumulation, skin irritation, and thermal discomfort during prolonged wear [12, 82, 83].

Achieving this balance requires co-design across materials and structures. Porous architectures such as electrospun nanofiber scaffolds and porous elastomeric networks provide high gas permeability and passive thermal and moisture management without sacrificing flexibility, while hydrogel-based substrates offer tissue-like modulus, inherent ionic conductivity, and bioadhesive contact that supports stable long-term interfacing. Collectively, these strategies establish a mechanically compliant yet physiologically compatible foundation that supports reliable, high-fidelity physiological data acquisition during long-duration use [36].

#### Nanofiber Architectures for High Breathability and Flexibility

Nanofiber-based architectures are essential for achieving the air and moisture permeability required for long-term skin-interfaced operation, because electrospun porous scaffolds can sustain gas exchange while remaining mechanically compliant under continuous wear. Recent progress has emphasized structural designs that actively regulate the microenvironment at the skin interface, including Janus hierarchical gradient honeycomb textiles that manage thermal and moisture gradients and maintain a dry, comfortable interface during prolonged use [84]. In parallel, controlling nanofiber alignment supports anisotropic sensing platforms, where fiber orientation governs directional response and supports selective multidirectional strain detection with improved signal discriminability [85].

**Fig. 5:**
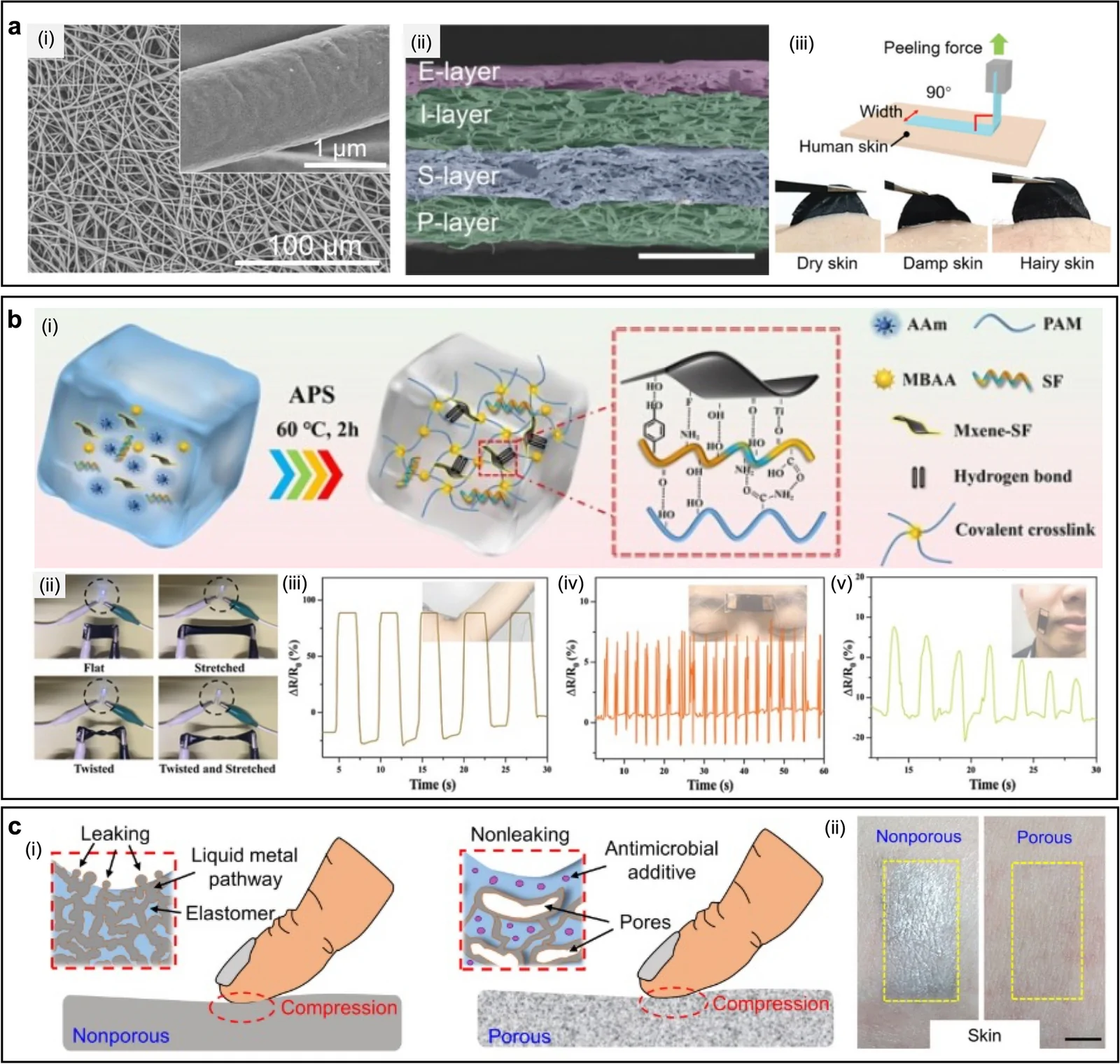
Conformal contact and breathable substrate designs for skin-interfaced electronics. **a** Top-view and cross-sectional SEM of SPRABE-skin, a schematic of the 90° adhesion test, and photographs on dry, damp, and hairy human skin. Scale bar 100 µm. Reproduced with permission [12]. Copyright 2023, Wiley–VCH. **b** PAM/(MXene-SF) hydrogel preparation schematic, circuit operation under stretching and twisting, and wearable responses (ΔR/R0) to elbow rotation, frowning, and smiling. Reproduced with permission [8]. Copyright 2024, Elsevier. **c** Compression comparison of nonporous and porous EGaIn composites, antimicrobial formulation, and 3-day skin-wear photographs. Scale bar 5 mm. Reproduced with permission [86]. Copyright 2023, American Association for the Advancement of Science (AAAS)

Beyond passive breathability, nanofiber scaffolds provide a versatile framework for integrating functional conductors and sensing layers without compromising permeability. A representative demonstration of nanofiber-supported system integration was reported by Hao et al. who developed a stretchable, breathable, and self-adhesive electronic skin using a scalable multilayer architecture that combines a fibrous thermoplastic polyurethane scaffold for tissue-like softness and permeability with MXene-based functional layers for both strain sensing and adhesive biopotential acquisition [12] (Fig. 5a). In this system, the fibrous scaffold exhibited a Young’s modulus of 3.36 MPa, while the MXene-CNT@TPU strain layer delivered ultrahigh sensitivity over a broad working range, with a maximum gauge factor of 63,494 at 485% strain. At the same time, the adhesive MXene-WPU electrode layer provided an adhesive strength of about 145 N m^−1^ even on dry, sweaty, and hairy skin, and showed lower skin–electrode interfacial impedance than commercial Ag/AgCl electrodes across 1–104 Hz, thereby enabling higher-fidelity ECG, EMG, and EEG acquisition under dynamic disturbances and supporting wireless long-duration monitoring in practical use conditions.

Nanofiber architectures also support broader functionality relevant to intelligent wearables, including self-powered electronic skins based on all-nanofiber triboelectric nanogenerators with breathable and antibacterial characteristics, and breathable MXene-supported epidermic sensors that couple photothermal functionality with ultrasensitive human–machine interaction [37, 87]. Finally, iontronic pressure-sensing formats based on all-nanofiber ionic membranes and porous electrodes provide a complementary route to high-performance pressure transduction while preserving ultraviolet shielding and antibacterial features, highlighting how breathability and data fidelity can be co-optimized within fibrous platforms [88, 89].

#### Conformal Contact Hydrogel Substrates for Skin-Interfaced Bioelectronics

Hydrogel substrates provide tissue-like mechanical compliance and intrinsic ionic conductivity, establishing an effective medium for stable skin-interfaced bioelectronics [90, 91]. Compared with conventional dry electrodes, hydrogels can form low-impedance and conformal contact on curvilinear, textured skin, which is particularly important for maintaining stable biopotential acquisition during long-duration wear. Recent studies have therefore emphasized ionic conductive hydrogels with high biological adaptability and low interface impedance for capturing weak electrophysiological signals, together with electrode designs that separate conductive and substrate-adhesive functions to improve long-term interface robustness [81, 92].

Advanced hydrogel formulations progressively incorporate functional fillers and network engineering to expand operating stability and sensing modalities. Li et al. reported an ultrastretchable and self-adhesive MXene nanocomposite hydrogel that addresses MXene oxidation and weak nanosheet to matrix coupling by introducing silk-fibroin-modified MXene into a polyacrylamide network, achieving high stretchability of 1560%, toughness of 165 kJ m_−3_, conductivity of 0.25 S m_−1_, and low interface impedance that supports reliable monitoring of weak electrophysiological signals alongside thermosensitive body-temperature readout [8] (Fig. 5b).

Beyond the material level, integrating bioadhesion into hydrogel hardware supports conformal wearable systems that remain stable under dynamic use while supporting richer sensing outputs. Collectively, these hydrogel substrate strategies provide a stable hardware foundation for continuous high-fidelity data acquisition that is required for intelligent diagnostic and interaction systems [90].

#### Porous Elastomeric Substrates for Robust and Permeable Electronics

Porous elastomeric substrates provide a practical hardware foundation for regulating heat and moisture at the skin-electronics interface while maintaining soft mechanical compliance. At the structural level, highly air- and water-permeable hierarchical mesh designs further support stretchable electronic skin patches that remain functional under submerged or high-humidity conditions, while gas-permeable and ultrathin porous electrodes reduce mechanical loading on the epidermis and support unobtrusive epidermal integration [82, 93].

Beyond comfort-related functions, structural refinement of porous substrates improves durability and safety in wearable hardware by addressing leakage, contamination, and signal consistency. Xu et al. reported phase separation-derived porous liquid metal–elastomer composites in which a porous damping mechanism suppresses deformation-driven leakage and reduced percolation thresholds help preserve conductive pathways during strain. At an EGaIn volume fraction of about 25%, the porous composite maintained an electrical conductivity of approximately 2.0 × 105 S m^−1^, which increased up to about 1.2 × 10^6^ S m^−1^ at 55% EGaIn. At the same time, the porous architecture provided a water vapor transmission rate of about 4900 g m^−2^ day^−1^ and reduced the elastic modulus to about 1.5 MPa, compared with about 16.7 MPa for the nonporous counterpart, thereby improving both breathability and tissue-level mechanical compliance. The material also showed only about 15% resistance change after 1000 cycles at 500% strain, exhibited no observable leakage before rupture at about 550% strain, and presented a magnetic susceptibility of − 3.23 ppm. Together with the antimicrobial functionality introduced by ε-polylysine, these characteristics enabled a mechanically stable, breathable, and MRI-compatible soft conductor that maintained high electrical performance under deformation, making it well suited for skin-interfaced bioelectronics for cardiac monitoring and mechanically imperceptible electrical stimulation [86] (Fig. 5c). Complementary material and architecture approaches include gradient porous elastomers combined with self-assembled silver nanowires to improve gas permeability and sensing fidelity, and breathable multilayer e-skin designs that reduce strain dependence of output to maintain consistent readout during daily use [13, 94]. Together, these porous elastomeric foundations expand breathable and mechanically compliant platforms toward robust, long-lasting wearable systems that can support high-quality physiological data streams for intelligent diagnostics. Taken together, these material strategies define the functional basis of intelligent wearable systems, but their practical impact depends on how reliably they can be translated into manufacturable, interconnected, and scalable device architectures. On this basis, the following section turns to fabrication and interconnection strategies that convert these material capabilities into integrated wearable systems.

## Innovative Manufacturing and Interconnection Strategies

The transformation of active soft materials into integrated intelligent wearable systems depends on manufacturing and interconnection strategies that preserve structural integrity while sustaining functional reliability [16, 95]. While material innovations define electrical performance and mechanical compliance, fabrication and packaging determine whether those properties remain reproducible when assembled into dense, deformable networks on skin-compatible platforms [14]. By combining high-resolution patterning with scalable architectures, diverse device functions can be integrated with accurate registration and robust interconnectivity without compromising softness [96, 97]. Consequently, these manufacturing advances provide the hardware basis for stable, high-fidelity signal transduction across large-area interfaces, enabling the practical deployment of sophisticated wearable systems. In this context, this section demonstrates how soft material functionalities are organized into reproducible system layouts through precise patterning, multilayer integration, and scalable interconnection, thereby bridging material capability and operational device architecture.

### High Resolution Patterning and Printing for Precise Circuitry

High-resolution patterning is a fundamental pillar in advanced wearable electronics because it supports miniaturized sensor arrays and dense integration of heterogeneous functions within restricted, curvilinear form factors [16, 98]. Such spatial resolution is often necessary for placing multiple sensing nodes on a single skin-interfaced patch, supporting applications such as spatial temperature mapping and high-density electrophysiological recording [99, 100]. In this context, conventional lithography remains a cornerstone because it can deliver submicrometer feature definition and high-throughput fabrication of dense circuitry on flexible substrates. The geometric fidelity of lithographic patterning directly influences signal integrity in high-density layouts by reducing parasitic coupling and electrical crosstalk between adjacent interconnects, thereby preserving channel clarity and improving the reliability of physiological readout [101].

Alongside lithography, maskless additive approaches have expanded as practical routes for patterning functional materials with higher design flexibility and improved material efficiency [102]. Inkjet printing supports selective deposition of conductive, semiconductive, and dielectric inks only where needed, reducing material waste and eliminating mask fabrication and aggressive etching steps. These approaches are particularly useful for soft electronics because they are often compatible with chemically sensitive organic materials and elastomers that cannot tolerate high thermal budgets. By combining lithography for extreme feature control with inkjet printing and laser-induced patterning for rapid, material-specific fabrication, manufacturing frameworks can be tailored to the mechanical and electrical constraints of each device layer while maintaining overall integration density and functional reliability [96].

#### Inkjet Printing of Functional Inks for Soft Electronics

Additive direct-write printing based on inkjet techniques provides a maskless and material-efficient route to pattern functional nanomaterials on nonconventional substrates, which is progressively important for soft electronics that must remain compatible with low thermal budgets and mechanically compliant form factors.

These digital processes support selective deposition of conductive, semiconductive, and dielectric inks with minimal material waste, and ink formulation together with droplet formation and wetting behavior becomes a central design variable that governs feature fidelity and electrical continuity after deformation. Inkjet printing has also been applied to active layers for soft sensing and bioelectronics, including graphene and PEDOT:PSS patterns on skin-conformable polyurethane for temperature sensing and biomimicking stretchable organic electrochemical transistors that support bioinspired signal transduction [99]. More broadly, recent perspectives and system-level demonstrations emphasize that printed nanomaterials can serve as a unifying platform for integrated flexible wearables, and sustainable process choices are progressively highlighted as practical constraints for scaling electronic textiles [95, 102].

**Fig. 6:**
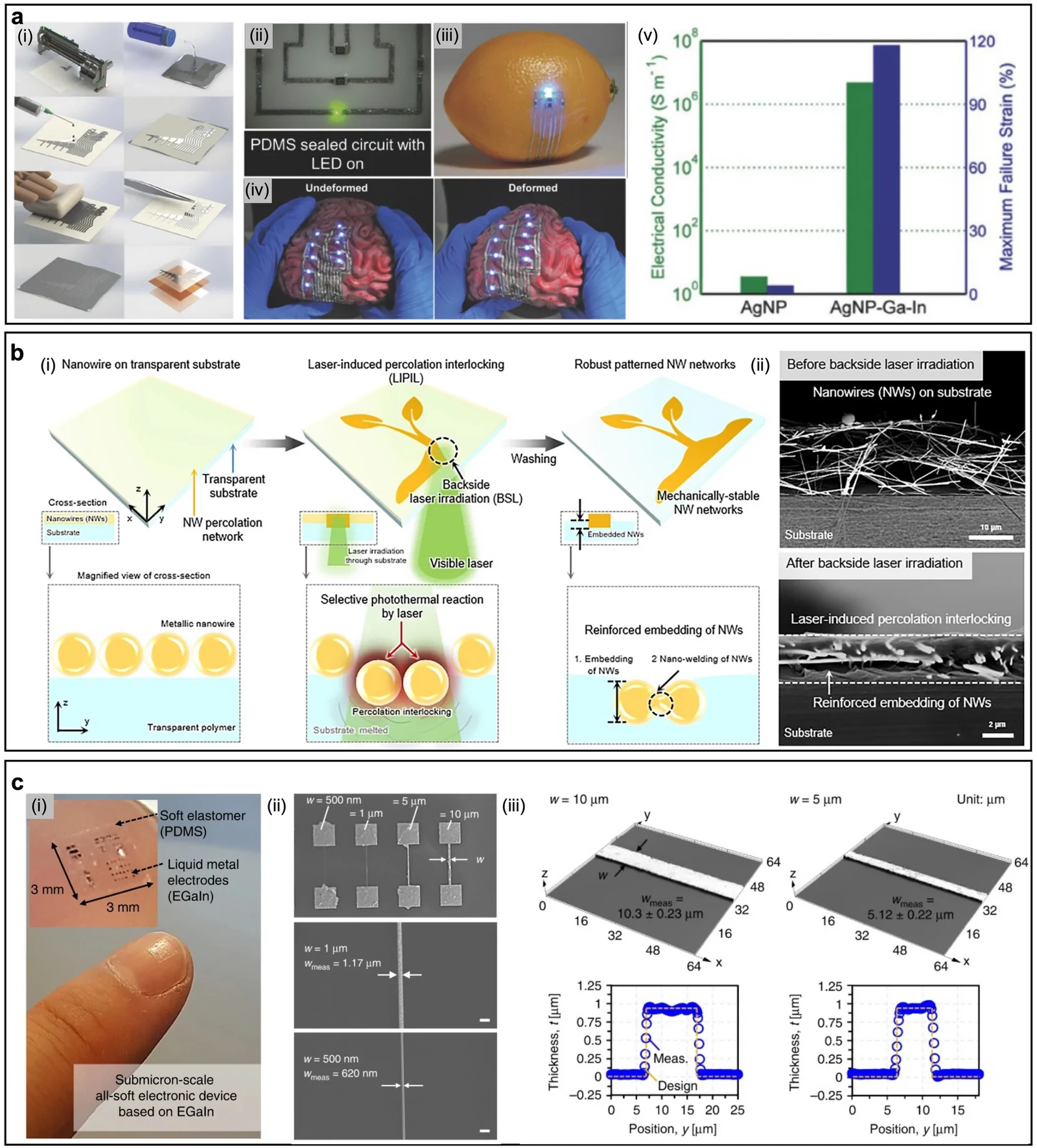
High-resolution patterning and printing routes for precise soft circuitry. **A** 3D hydrographic printing of ultrathin soft electronics, including printed AgNP traces, an AgNP–Ga–In circuit with surface-mounted components, hydrographic transfer onto 3D objects, LED operation under deformation, and conductivity and failure-strain changes after EGaIn deposition. Reproduced with permission [103]. Copyright 2018, Wiley–VCH. **b** Laser-Induced Nanowire Percolation Interlocking, including a process schematic and cross-sectional SEM images before and after backside laser irradiation. Reproduced with permission [96]. Copyright 2025, Springer Nature. **c** Soft-material encapsulation and release of EGaIn micro–nano lines, including SEM images from 10 µm to 500 nm and 3D profiles and cross-sections of representative line widths. Scale bar 2 µm. Reproduced with permission [101]. Copyright 2020, Springer Nature

A key bottleneck for printed metallic conductors on heat-sensitive substrates is the conversion of nanoparticle inks into percolated conductive networks, because conventional thermal sintering can deform polymer substrates or degrade soft matrices. Tavakoli et al. introduced an EGaIn-assisted room-temperature sintering strategy for inkjet-printed silver nanoparticle traces that uses a thin EGaIn coating to bind Ag nanoparticles into a continuous conductive pathway, increases conductivity by orders of magnitude, and improves tolerance to tensile strain while enabling transferable tattoo-like thin-film circuits for conformal integration on complex three-dimensional surfaces such as skin [103] (Fig. 6a).

Inkjet manufacturing is also expanding from planar patterns toward integrated three-dimensional and fluidic architectures that reduce interconnect burden by co-fabricating multiple functions within a single platform.

A representative direction is 3D-printed epifluidic electronic skin designed for multimodal data capture and machine-learning-based health surveillance, underscoring that low-temperature material compatibility and interlayer adhesion are decisive for maintaining data integrity in continuously operating wearable systems [25].

#### Laser-Induced Patterning and Scribing for Nanostructured Functional Circuits

Laser-induced patterning and scribing provide a maskless, high-precision fabrication route for nanostructured functional circuits on soft hardware, enabling localized conversion or restructuring of materials without exposing the entire substrate to harsh chemicals or high thermal budgets. By directly transforming precursors into conductive or semiconductive networks with spatial selectivity, laser strategies support rapid prototyping of high-resolution layouts while preserving the mechanical compliance and breathability that are often required for wearable platforms.

A persistent reliability challenge in soft electronics is the weak adhesion and mechanical fragility of metallic nanowire networks on deformable substrates, which can lead to delamination and performance loss under repeated deformation. Jung et al. reported a generalized laser-induced nanowire percolation interlocking process in which interfacial photothermal energy drives interpenetration between the nanowire network and the thermoplastic matrix, creating a mechanically interlocked percolation network that improves adhesion and robustness without relying on an insulating protective layer and supports reusable wearable physiological sensors as well as stable functionalization in wet environments [96] **(**Fig. 6b**)**.

In addition to metallic systems, laser processing progressively serves as a tool for local property engineering across carbonaceous and polymeric platforms, allowing designers to tune porosity, microstructure, and interfacial bonding in a spatially programmable manner. Laser-induced conversion can also generate MOF-derived flexible electrodes with microscale definition for electrochemical sensing, extending laser fabrication from geometric patterning to functional electrode synthesis [26]. Collectively, these approaches position laser-induced manufacturing as a versatile route to robust, material-specific circuit formation that supports long-duration wearable operation and stable data acquisition.

#### Microscale Patterning Strategies for High Resolution Flexible Electronics

Microscale patterning strategies are essential for achieving the device density and functional complexity required for next-generation flexible electronics, particularly as wearables evolve toward multifunctional on-skin computing platforms. High-resolution patterning of conductive and semiconductive materials reduces footprint while enabling dense sensor arrays and local signal-conditioning circuits, which is difficult to realize through low-resolution direct-write approaches alone. Accordingly, photolithography, transfer printing, and microcontact printing provide scalable routes to compact layouts that preserve mechanical compliance through appropriate structural design, supporting high-fidelity signal acquisition and processing on stretchable substrates [98].

Kim et al. reported a nanofabrication strategy for all-soft and high-density electronic devices based on eutectic gallium indium, in which a hybrid approach combining electron-beam lithography with soft lithography patterns a biphasic structure that embeds high-resolution EGaIn features within elastomeric matrices and achieves feature sizes down to 180 nm with 1 μm line spacing, thereby enabling an unusually high combination of resolution, conductivity, and wiring density in soft interconnects that can remain functional under deformation [101] (Fig. 6c).

Beyond liquid metals, microscale patterning supports high-resolution integration of soft organic and permeable textile electronics. Multilayer patterning strategies for intrinsically stretchable electronics highlight how stacked architectures can increase functional density without sacrificing deformability, while sacrificial-layer-assisted nanoscale transfer printing provides a reliable route to assemble high-resolution components with controlled interfaces [16, 104]. In parallel, photo-patternable PEDOT:PSS hydrogels expand microscale lithography into bioelectronic interfaces by enabling fine patterns through UV-curable chemistries, and in-textile photolithography addresses substrate roughness by enabling localized polymerization directly on fibers while maintaining permeability and flexibility [100, 105]. Collectively, these microscale fabrication approaches build the manufacturing basis for compact, mechanically compliant, and high-density soft systems suitable for continuous wearable monitoring.

### Scalable Manufacturing and High Density Interconnect Architectures

The transition of wearable electronics from laboratory prototypes to integrated systems requires large-area scalable manufacturing that can deliver uniform performance over the body’s wide and curvilinear surfaces, because practical “smart skin” deployment depends on both meter-scale coverage and reproducible device characteristics across many sensing nodes [14]. High-throughput processes such as roll-to-roll manufacturing and automated large-scale printing support cost-effective production and consistent layer deposition, which supports multi-node arrays that capture systemic physiological information with stable quality and reduces device-to-device variability that can otherwise limit model generalization and increase calibration burden [106, 107]. As functionality grows to include multimodal sensing, local signal processing, and power modules, scalability must be coupled with high-density integration through multilayer stacking and mechanically compatible interconnect architectures, where vertical routing and through-via technologies alleviate planar wiring congestion without sacrificing conformability [15, 97]. Finally, complex circuit geometries that exploit three-dimensional deformation modes can mitigate the trade-off between stretchability and active area ratio, allowing dense electronics to remain mechanically robust under extreme multi-axial deformation while maintaining a compact on-skin footprint, which collectively establishes the hardware foundation for reliable operation of next-generation intelligent wearables [108, 109].

#### Large Area Scalable Manufacturing for Wearable Electronic Systems

Large-area scalable manufacturing is a fundamental requirement for translating soft electronics from laboratory prototypes to deployable wearable products, because integrated wearable systems must maintain uniform electrical performance and mechanical integrity across expansive, deformable substrates. High-throughput manufacturing routes such as roll-to-roll processing and continuous solution-based printing support rapid production of functional layers at reduced unit cost while improving reproducibility, which is essential for multi-node sensor arrays that must generate consistent data quality at scale.

**Fig. 7:**
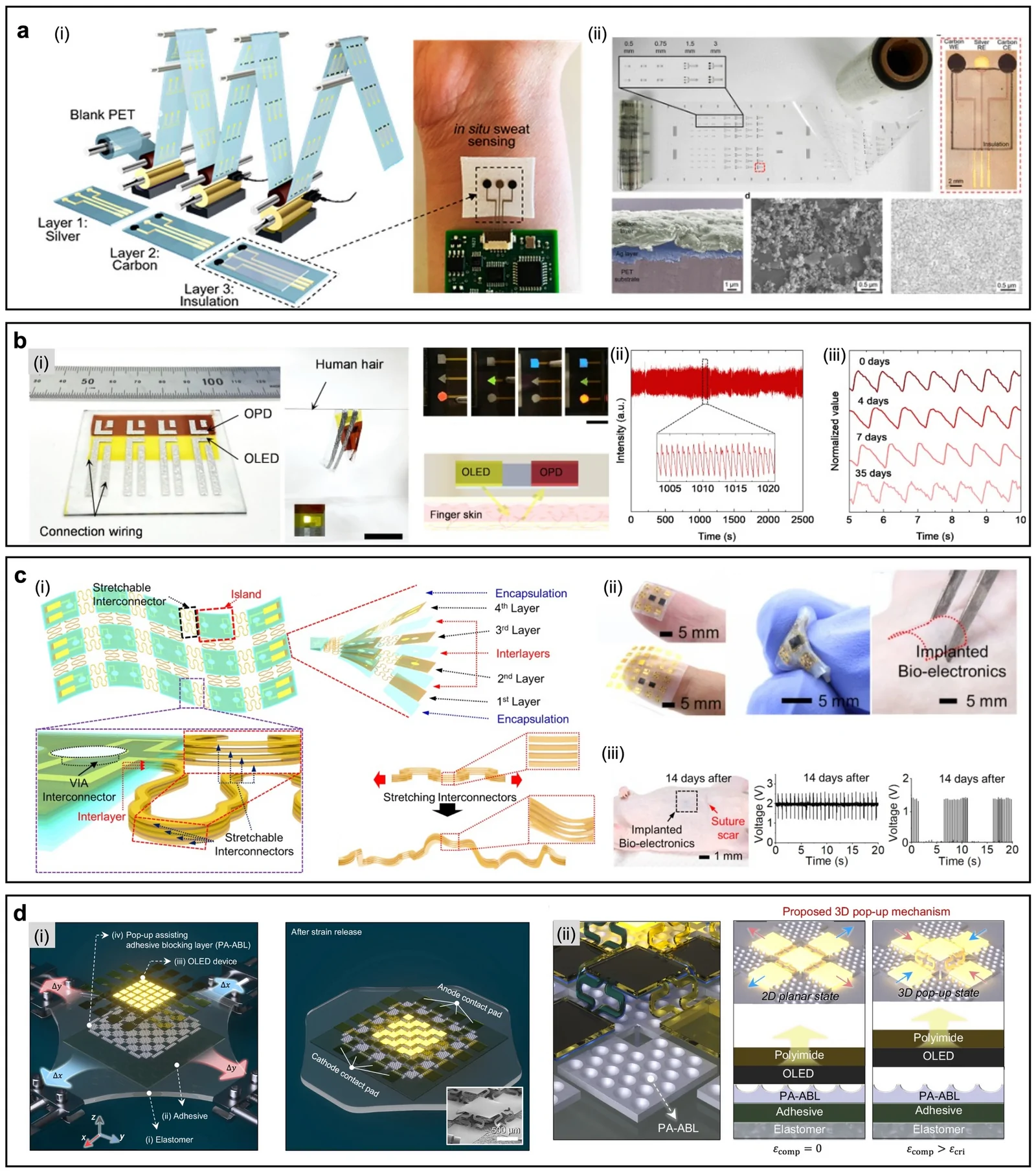
Scalable manufacturing and high-density interconnect architectures for soft bioelectronics. **a** Roll-to-roll gravure-printed PET electrode arrays using silver, carbon, and insulation layers, with roll-scale production and optical/SEM characterization. Reproduced with permission [14]. Copyright 2018, American Chemical Society. **b** All-solution-processed ultrathin OLED/OPD photoplethysmography system, including device photographs, multicolor displays, operation schematic, and long-term pulse-wave and air-stability results. Scale bar 1 cm. Reproduced with permission [110]. Copyright 2024, American Association for the Advancement of Science (AAAS). **c** Vertically-Separated Multilayer Stretchable Circuit with rigid islands and vertically separated stretchable interconnectors, including multilayer schematics, deformation illustrations, fabricated devices, and in vivo ECG sensing and stimulation. Reproduced with permission [97]. Copyright 2024, Springer Nature. **d** PA-ABL–assisted OLED assembly on biaxially prestrained elastomers, including planar-to-pop-up transition schematics and representative device images. Reproduced with permission [109]. Copyright 2024, Springer Nature

Bariya et al. implemented roll-to-roll gravure printing to produce electrode arrays on continuous rolls of flexible substrates, achieving highly uniform electrochemical kinetics by optimizing both ink formulation and the resulting microstructure. They further showed that the same printed electrodes can be readily functionalized into sensors with reproducibly high performance across multiple analyte targets in noninvasively collected biofluids, including real-time sweat tracking during exercise, thereby highlighting a practical pathway to low-cost, disposable wearable electrochemical sensors [14] (Fig. 7a). Sun et al. established all-solution processes for ultrathin organic optoelectronics using a universal trilayer device structure and demonstrated a large-area self-powered photoplethysmogram sensor array integrated with a large-area organic solar module, highlighting how complex multimaterial functions can be manufactured on ultrathin substrates without vacuum-intensive steps [110] (Fig. 7b). Beyond device examples, scalable manufacturing progressively targets interconnect and architecture complexity, including full roll-to-roll fabrication of through-substrate vias for double-sided wearable electronics and continuous production of patterned multilayer elastic substrates with liquid metal wiring, which together expand large-area processing from single-layer printing toward high-density and multilayer system integration [15, 107].

Scalable manufacturing in intelligent soft electronics should therefore be evaluated not only by throughput, cost, or achievable device area, but also by how reproducibly device and signal characteristics are preserved across fabrication batches and extended use. Even when the nominal device design remains unchanged, variations in material deposition, microstructural uniformity, interfacial integrity, and encapsulation quality can alter sensor baselines, sensitivities, and noise statistics. These variations become increasingly consequential as manufacturing scales, because they raise calibration burden and can reduce the transferability of calibration models across devices produced under different process windows. For this reason, scalability and standardization should be considered together. In practice, this requires process-aware quality control, standardized electrical and mechanical benchmarking, and fabrication-aware calibration frameworks that link manufacturing conditions with sensor outputs, so that large-area manufacturing supports not only hardware yield but also reproducible data generation for artificial-intelligence-enabled wearable systems [106, 111].

#### Multilayer Stretchable Interconnects and Advanced Through Via Technologies

High-performance wearable hardware progressively requires a transition from laterally sprawling two-dimensional layouts to vertically integrated three-dimensional architectures, because high-density multimodal systems quickly become wiring-limited when all routing must occur in a single plane. Although serpentine interconnects can accommodate stretchability, their large lateral footprint limits the achievable density of sensors, emitters, and processing units on a single patch. Multilayer stacking and advanced through-via strategies address this bottleneck by enabling vertical routing among functional layers, which supports compact form factors while maintaining mechanical compliance required for skin and soft-tissue interfaces.

Jung et al. reported a multilayer stretchable electronics design approach that preserves a degree of stretchability comparable to single-layer systems while achieving a much more compact lateral form by controlling strain distribution across stacked layers, supported by experimental and computational analyses and demonstrated through multifunctional stretchable implantable bioelectronics and a stretchable multilayer passive-matrix LED array [97] (Fig. 7c).

Beyond multilayer routing, complementary directions in three-dimensional integration include scalable fabrication of large-area stretchable circuits and laser-supported approaches that embed or pattern conductive structures and functional devices within polymer matrices, providing additional pathways to compact, mechanically robust 3D circuitry [112, 113].

At the system level, 3D integration can also expand modality and bandwidth by co-integrating electronics with photonic or optoelectronic elements on deformable substrates, which has supported waterproof stretchable optoelectronics for biomedical and robotic functions and flexible electronic–photonic integration using ultrathin polymer chiplets [114, 115].

#### Complex Circuit Architectures for High Density Stretchable Systems

High-density circuitry on stretchable substrates is often constrained by planar geometric trade-offs, because conventional two-dimensional rigid-island and elastic-bridge layouts rely on long serpentine interconnects that consume substantial area and reduce the active-area ratio while still leaving wiring density limited within a single plane. This limitation becomes especially restrictive for high-resolution displays and dense sensing systems, where maximizing functional surface area must be balanced with compact routing, electrical performance, and mechanical reliability under repeated deformation. Accordingly, complex circuit architectures progressively exploit three-dimensional deformation modes and routing strategies that increase interconnect density without expanding lateral footprint or introducing mechanically fragile bottlenecks [108].

For instance, Kim et al. proposed a three-dimensional buckled height-alternant architecture for stretchable OLEDs that achieves a high initial active-area ratio of 85% together with a system strain up to 40% by combining an optimized dual serpentine design with spatially selective adhesion control that supports reliable transition into a stable three-dimensional buckled state, and the resulting devices maintained performance over 2000 biaxial cycles at 40% system strain while also enabling passive-matrix 7 by 7 displays with comparable area ratio and stretchability [109] (Fig. 7d). Yet even well-integrated and scalable architectures do not by themselves guarantee usable physiological information during real operation. Once these systems are deployed on dynamic, skin-interfaced environments, the next challenge becomes preserving signal fidelity and sustaining autonomous function under noise, deformation, and power constraints, which is the focus of the following section.

## Hardware Optimization for High-Fidelity Signal Acquisition and Energy-Autonomous Wireless Systems

The transition from active materials to integrated intelligent systems is enabled by the comprehensive optimization of hardware architectures to ensure high-fidelity signal acquisition and operational autonomy [116, 117]. While advancements in materials and manufacturing provide the essential structural framework, hardware-level refinement is indispensable for capturing precise physiological data within dynamic, skin-interfaced environments. This multifaceted optimization involves the synergistic integration of engineered sensing interfaces, localized signal conditioning, and energy-efficient power management strategies. By establishing these robust hardware foundations, the platform can minimize signal distortion and ensure the continuous, self-sustained operation required for advanced data analytics [118, 119]. This hardware-level refinement bridges raw sensing performance to the reliable, long-term deployment of next-generation intelligent wearable systems [18, 28]. Accordingly, this section addresses the operational layer of intelligent soft systems, demonstrating how sensing interfaces, local signal conditioning, and power management must work together to convert integrated device architectures into stable and continuously usable data platforms.

### Structural Engineering for Enhanced Sensitivity and Linearity

Structural engineering within active sensing layers provides a practical route to translate intrinsic material properties into system-level performance, because signal fidelity is governed not only by transduction chemistry but also by how stresses are distributed and converted into measurable electrical changes [27, 120]. While the chemical composition of soft materials defines the basic sensing mechanism, the physical architecture of the sensing medium strongly influences responsiveness, linearity, and stability by shaping contact evolution, strain concentration, and effective deformation pathways [121, 122]. In skin-interfaced electronics, homogeneous or unstructured active layers frequently yield limited performance because bulk mechanical responses can dilute stimulus localization and promote early saturation or nonlinear output. Accordingly, geometric tailoring of the active layer has become a central design lever for controlling how external stimuli couple into the sensing interface, enabling signal characteristics that exceed what is typically achievable with flat-film configurations [123].

A key goal of structural modification is to navigate the long-standing trade-off between sensitivity and sensing range while preserving linearity across physiologically relevant regimes. By re-engineering morphology and internal configuration, engineered architectures can introduce progressive deformation, multiscale compliance, and controlled contact modulation, which together create tunable transduction pathways for specific monitoring tasks. This approach can simultaneously support a high effective active-area ratio and enhanced mechanical deformability, allowing stable electrical outputs to be maintained from subtle pressures to larger loading conditions without abrupt clipping. Such architecture-driven tuning establishes a reliable data foundation that is suitable for continuous wearable monitoring and downstream data-driven analysis.

#### Microstructured Surface Engineering using Micro-Domes and Pyramids

Microstructured surface engineering is a core strategy for enhancing tactile-sensor sensitivity and signal quality, because microscale geometries amplify contact modulation under small loads and translate subtle mechanical stimuli into larger electrical changes. Compared with flat interfaces that exhibit limited contact-area evolution, micro-domes, pyramids, and ridge-like patterns introduce localized stress concentration and progressive deformation, which can increase capacitance or resistance contrast in low-pressure regimes and improve feature separability for downstream analysis.

**Fig. 8:**
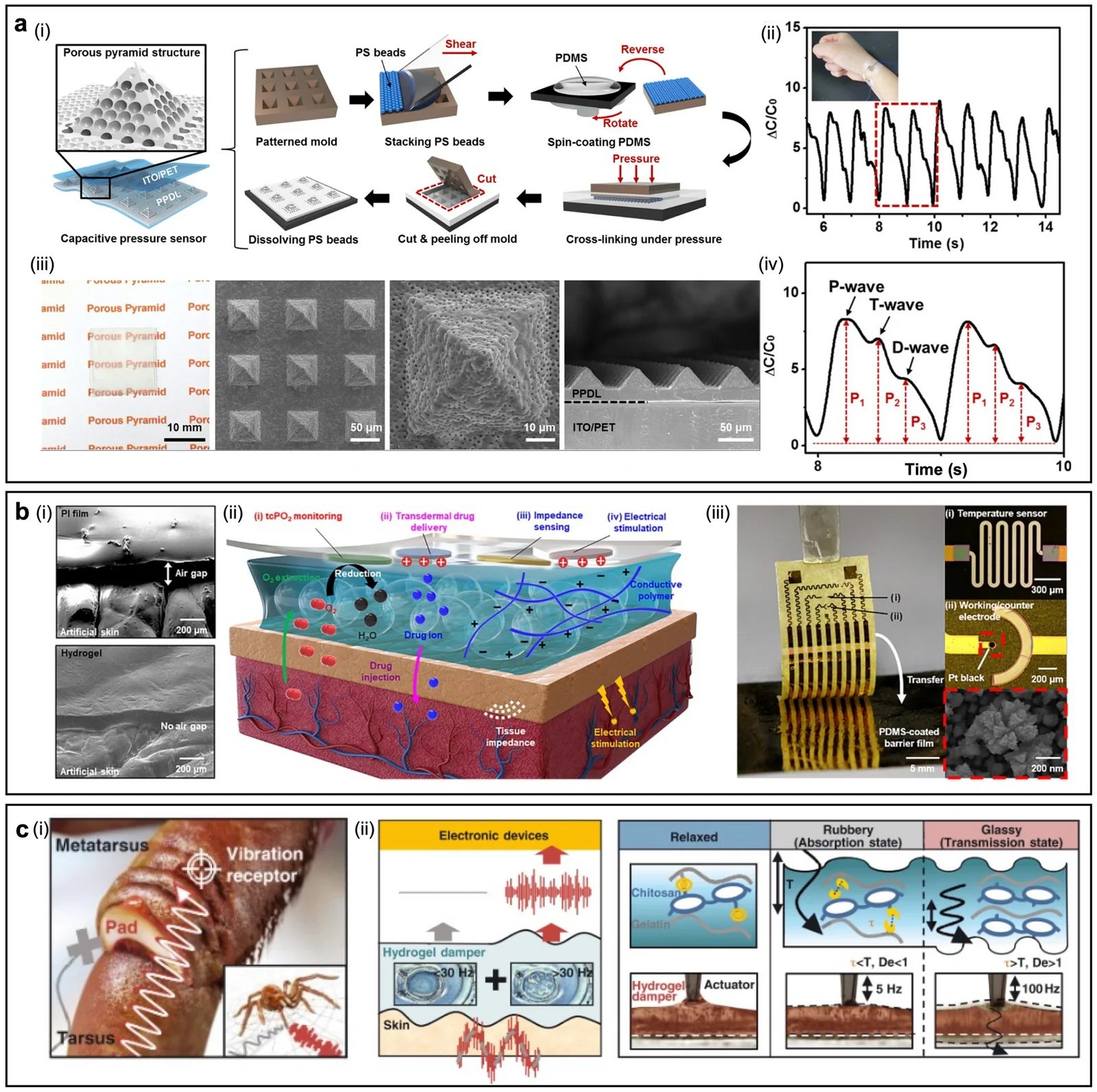
Hardware optimization for high-fidelity signal acquisition and energy-autonomous wireless systems. **a** Capacitive pressure sensor based on a porous pyramid dielectric layer (PPDL), including a PPDL fabrication schematic, optical photo of PPDL on ITO/PET, top-view and cross-sectional SEM images, and real-time radial-artery pulse signals with a magnified segment. Reproduced with permission [27]. Copyright 2019, American Chemical Society. **b** SEM images of PI film and tissue-like ultrasoft hydrogel mounted on artificial skin, and wearable transcutaneous biosensors for tcPO2 and local tissue impedance on PDMS. Reproduced with permission [116]. Copyright 2021, American Association for the Advancement of Science (AAAS). **c** Bioinspired selective frequency damping through a hydrogel damper, showing low-frequency suppression, high-frequency signal transmission, and relaxation-time-dependent filtering. Reproduced with permission [118]. Copyright 2022, American Association for the Advancement of Science (AAAS)

For instance, Yang et al. reported an ultrasensitive capacitive pressure sensor based on a porous pyramid dielectric layer, where internal porosity reduced the effective compressive modulus and amplified pressure-induced changes in effective dielectric constant to reach 44.5 kPa^−1^ below 100 Pa, and the device architecture further decoupled pressure response from substrate strain by placing the sensing elements on hard elastomer islands embedded in a soft matrix while remaining nonresponsive to temperature; the same porous pyramid layer was also shown to support a contact-resistance pressure-sensing mode after conductive-polymer grafting, illustrating a versatile microstructure platform for low-pressure, high-fidelity tactile transduction [27] (Fig. 8a).

In parallel, interlaced ridge-like microstructures offer another structural lever to broaden usable ranges by shaping contact evolution across multiple deformation stages, which can be advantageous when paired with ionic transduction mechanisms that benefit from stable geometric modulation [122].

#### Hierarchical and Porous substrate for Broad Sensing Range

A central trade-off in soft pressure sensing is the inverse coupling between sensitivity and pressure-response range, because microstructured surfaces can amplify low-pressure signals yet often saturate early when structural compressibility is insufficient. Hierarchical and porous substrates can mitigate this limitation by enabling progressive deformation across multiple length scales, delaying saturation while maintaining resolution from subtle physiological loading to larger mechanical inputs and improving output linearity. As a representative example, Bai et al. reported an intrafillable microstructure strategy that simultaneously boosts sensitivity and broadens the pressure-response range by introducing undercuts and grooves that accommodate deformed surface microstructures and effectively increase structural compressibility [120]. The resulting iontronic sensor achieved a minimum sensitivity above 220 kPa^−1^ across a broad regime spanning 0.08 Pa–360 kPa, together with an ultrahigh pressure resolution of 18 Pa or 0.0056% over the full range and remarkable mechanical stability, highlighting how geometric engineering can transform early saturation into staged contact evolution without compromising low-pressure fidelity.

Beyond this design, related hierarchical strategies pursue the same goal by steering contact evolution and redistributing deformation into multistage pathways. Graphene aerogel architectures with controlled porosity support wide-range piezoresistive responses and feature-rich signals suitable for activity recognition and motion detection, while epidermis-inspired random microstructures and hierarchical CNT and PDMS surfaces provide additional routes to improve sensitivity and linearity through geometry-driven contact regulation rather than reliance on a single deformation mode [124, 125].

### In Sensor Signal Conditioning for Noise Suppression and Amplification

While the structural optimization of sensing layers significantly enhances raw sensitivity, the practical utility of wearable systems is ultimately governed by the quality and fidelity of the acquired electrical signals. In the inherently noisy environment of continuous physiological monitoring, raw data are often degraded by electromagnetic interference, baseline drift, and mechanically induced disturbances. In-sensor signal conditioning has therefore emerged as a key hardware-level strategy for preserving signal quality at the point of acquisition, rather than relying solely on back-end digital processing [118]. By integrating conditioning functions directly into soft bioelectronic platforms, researchers can reduce signal distortion during collection and transmission, ensuring that the acquired data more faithfully represent the underlying physiological state [28].

The realization of robust in-sensor conditioning therefore requires a coordinated strategy spanning the signal path from the physical interface to the sensor architecture. This includes stabilizing the sensor-skin interface to maintain low-impedance pathways, which is essential for reducing thermal noise and preserving contact reliability during wear [116]. It also involves structural and material designs that suppress mechanically induced disturbances before they propagate into the electrical readout, thereby mitigating motion artifacts at the source. Together, these hardware-level strategies provide a cleaner and more stable signal foundation for the reliable integration of wearable systems with downstream artificial intelligence algorithms for real-time diagnostics.

#### Material-Driven Interface Stabilization for Low Impedance

The fidelity of bioelectrophysiological recordings is strongly governed by the electrical stability of the skin–electrode junction, because microscale gaps and interfacial dehydration can elevate contact impedance and amplify noise at the point of measurement.

Compared with tissue-like interfaces, conventional dry electrodes frequently suffer from impedance fluctuations due to incomplete conformal contact and mechanical mismatch, which can obscure weak physiological features that must be preserved for reliable downstream analysis.

Lim et al. developed an ultrathin functionalized hydrogel that creates a tissue-mimicking quasi-solid interlayer on skin while sustaining exceptionally high mass permeability together with low interfacial impedance. At the contact region, the hydrogel acts as a liquid-like electrolyte, yet the overall structure remains mechanically robust enough to maintain an ultraconformal junction with low impedance, supporting both wearable electrochemical biosensors and electrical stimulators. In addition, the hydrogel’s porous, ultrathin architecture facilitates rapid transport of target species across the interface, thereby maximizing device performance [116] (Fig. 8b).

Beyond any single material choice, stable low-impedance interfacing typically requires two complementary levers, namely increasing effective interfacial contact while preserving wear comfort and improving ion-to-electron coupling to lower electrochemical impedance without relying on external gels. These goals motivate polymeric and gel-based conductors that provide mixed conduction pathways and sustain interfacial hydration [9].

#### Structural Design and Adaptive Filtering for Motion Artifact Mitigation

Motion artifacts remain a major bottleneck in wearable bioelectronics because relative micro-slips and dynamic mechanical disturbances at the skin–electrode interface can introduce signal components that overlap with physiological bandwidths.

Although digital filtering can suppress some noise, artifacts that share similar spectral content with biosignals are difficult to remove without distorting the signal of interest, which motivates hardware strategies that reduce artifact generation at the source.

Accordingly, structural design and hardware-level filtering aim to reshape how external mechanical energy reaches the sensing interface so that physiological information is preserved while mechanically induced disturbances are attenuated before they enter the electrical pathway.

Park et al. presented a viscoelastic gelatin–chitosan hydrogel damper inspired by spider cuticular pads as an unconventional, material-based bandpass filter that selectively suppresses motion-induced artifacts commonly introduced by unexpected patient movements, including walking and respiration, below roughly 30 Hz. This approach addresses a limitation of conventional digital bandpass filtering, which can remove artifacts but may do so at the cost of signal loss. The hydrogel exhibits a frequency-dependent phase near 30 Hz, where low-frequency components are damped in a rubbery state, whereas higher-frequency target signals are transmitted in a glassy state. Moreover, the damping regime can be shifted by tuning the relaxation time, with the transition frequency moving from 0.89 Hz at 27 °C to 51.8 Hz at 45 °C, highlighting the adaptability of the platform to different signal environments. As a result, it enables acquisition of higher-quality mechanical and electrophysiological signals while reducing reliance on heavy signal processing [118] **(**Fig. 8c**).**

In addition to physical dampers, motion-artifact mitigation commonly follows two complementary structural levers, geometric strain isolation that prevents bulk deformation from concentrating at the sensing region, and interface engineering that stabilizes contact under dynamic conditions.

Kirigami-based epidermal electrodes and kirigami-like multilayer designs distribute strain away from the sensing zone through engineered cut patterns, while strain-isolating materials and interfacial physics strategies further reduce mechanical energy transfer into the active region to support stable wireless monitoring [28, 126].

### Energy-Efficient Wireless Connectivity and Autonomous Power Management

Truly autonomous and untethered wearable systems require overcoming the power-supply bottleneck through energy-efficient wireless connectivity and autonomous power management. While the preceding sections addressed how to acquire and condition high-fidelity signals, long-term practical use is often limited by reliance on bulky, rigid batteries that poorly match the mechanics of skin-interfaced hardware. Bridging high-performance sensing with sustainable operation therefore requires power architectures that enable energy-efficient data transmission and local processing, so that continuous monitoring remains feasible without frequent user intervention and delivers a practical low-maintenance experience [18, 127].

Sustainable wearable ecosystems are commonly advanced along two complementary directions that connect communication and energy autonomy. Simultaneous wireless data and power transfer interfaces reduce system complexity by delivering energy and maintaining a wireless link through a unified channel, which can lower overall hardware burden [128]. In parallel, harvesting-driven energy management supports a shift toward battery-free operation by extracting energy from ambient sources such as biomechanical motion, thermal gradients, and electromagnetic fields, allowing sensing and wireless modules to operate with reduced dependence on conventional storage [17, 129]. Together, these strategies provide a pathway toward self-sustaining platforms that support continuous data streams for advanced analytics and improve the long-term reliability of next-generation intelligent wearables.

#### Simultaneous Wireless Data and Power Transfer (SWIPT) Interfaces

Fully untethered intelligent wearables require wireless power reception and data communication to operate reliably within a single conformable platform, because frequency detuning and coupling loss can arise when antennas and power components deform on skin. In this context, frequency detuning refers to an unintended shift of an antenna’s resonant frequency caused by deformation or changes in the surrounding dielectric environment, which can degrade impedance matching and reduce wireless power transfer and communication efficiency. Accordingly, SWIPT-oriented hardware design emphasizes electromagnetic stability under strain together with deformation-tolerant energy storage and interconnect architectures that maintain resonance and charging efficiency during use.

**Fig. 9:**
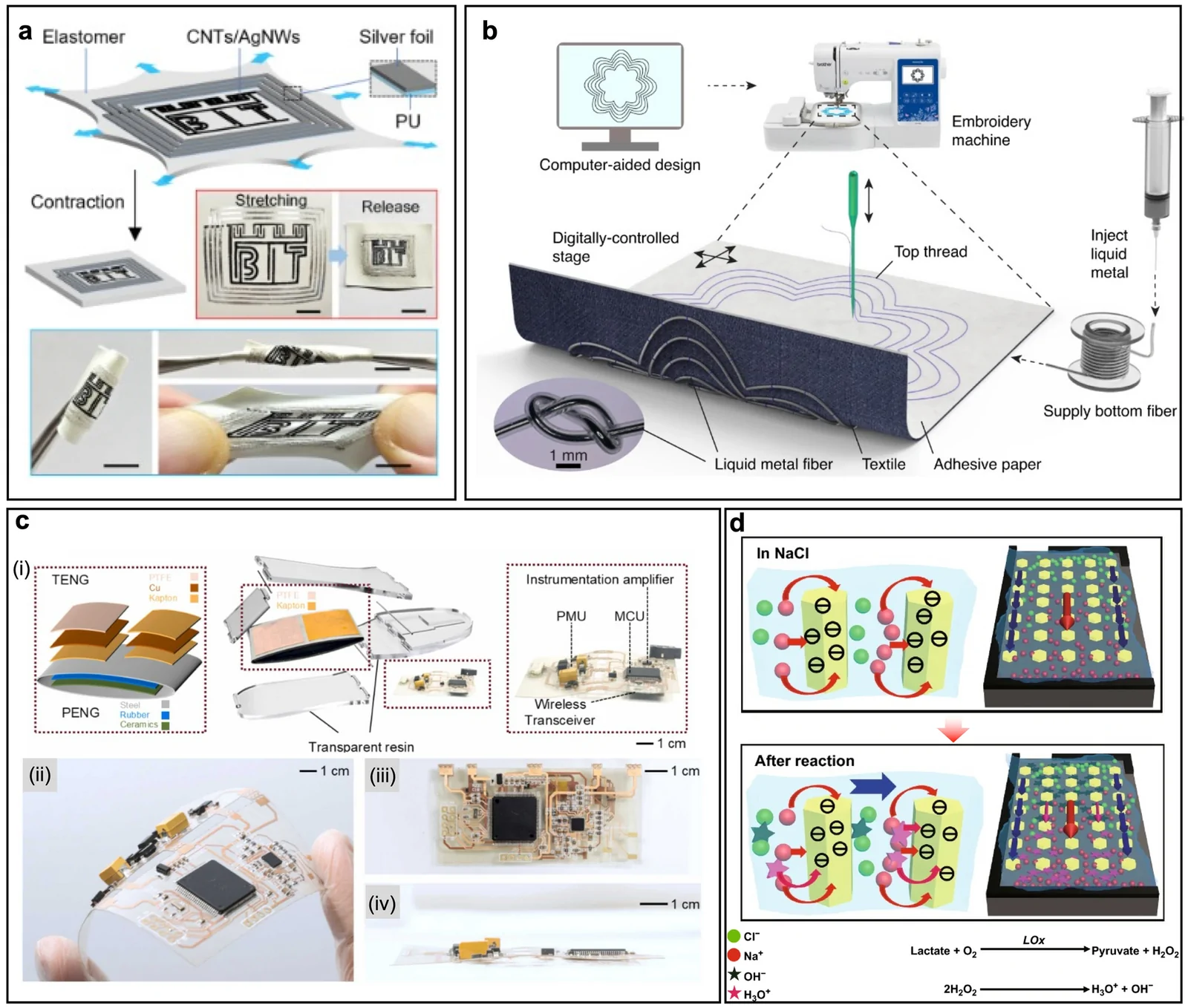
Self-powered and energy-harvesting approaches for intelligent soft electronics.** a** Schematic and profile display of stretchable integrated circuit fabrication via an omnidirectional stretch–contraction strategy, with photos of the circuit on an elastic balloon in stretched and released states and flexibility demonstration. Scale bar 1 cm. Reproduced with permission [117]. Copyright 2025, American Association for the Advancement of Science (AAAS). **b** Schematic illustration of the digital embroidery process, in which liquid metal fibers are prepared by filling perfluoroalkoxy alkane tubing with galinstan. Reproduced with permission [127]. Copyright 2022, Springer Nature. **c** Schematic and photographs of a self-powered wearable heart-rate monitoring system, including a 3D-printed sole with TPHG and a data-processing module, and FPCB images in planar, folded, and side views. Reproduced with permission [119]. Copyright 2025, Elsevier. **d** Schematic illustration of the power generation and biosensing operation of the perspiration-analyzing sites. Reproduced with permission [130]. Copyright 2020, Springer Nature

Wang et al. introduced dual-plating in-plane microbatteries that combine mechanical stretchability with wireless recharging, and they coupled the devices to circuit platforms using an omnidirectional stretch–contraction integration scheme enabled by mask-assisted printing. Under applied strain, folding-like geometries emerge, and the exclusion of active materials from deforming regions helps maintain electrochemical function under large shape changes, offering tolerance up to about 200% omnidirectional strain. Power and energy densities were reported to be approximately one order of magnitude higher than those of previously reported in-plane microbatteries, and this capability was leveraged to realize a stretchable display circuit that can be wirelessly charged as well as an electronic skin that supports touch-like detection of weight, temperature, and object shape [117] (Fig. 9a).

Beyond deformation-tolerant energy storage, textile-integrated SWIPT systems must also preserve electrical performance under repeated bending, washing, and daily wear without sacrificing the flexibility and permeability of clothing materials. Lin et al. demonstrated digitally embroidered liquid–metal electronic textiles in which galinstan-infiltrated perfluoroalkoxy alkane tubing was patterned onto garments through computer-controlled embroidery to realize near-field wireless power transfer and communication in a breathable and conformal format. By overcoming the conventional trade-off between electrical conductivity and mechanical robustness in conductive textile threads, their platform maintained stable resistance under folding and twisting, tolerated repeated washing and drying cycles, and enabled robust wireless connectivity with nearby wearable or implantable devices during daily activities and strenuous exercise [127] (Fig. 9b). Rather than relying on rigid add-on modules or coated conductive layers, this approach embeds wireless functionality directly into the textile architecture itself, making garment-level integration more compatible with daily wear.

In addition to energy storage, deformation-stable wireless links depend on antenna and interface designs that suppress resonance shifts and maintain coupling under stretching. Highly stretchable wireless powering antennas provide one route to stable resonance control, while epidermal RF antennas supported by nanofiber networks and liquid metal textile conductors extend SWIPT-compatible interfaces toward breathable and wearable form factors without sacrificing conductivity [128, 131].

#### Harvesting-Driven Energy Management for Battery-less Wireless Systems

Battery-less wearables represent a key step toward long-term, low-maintenance monitoring, because rigid batteries impose form-factor constraints and replacement or recharging interrupts continuous use. Harvesting-driven energy management addresses this limitation by converting ambient and biomechanical energy into usable electrical power and regulating it to support sensing and wireless communication with minimal stored energy.

Notably, Wu et al. demonstrated a self-powered heart-monitoring system in which a triboelectric and piezoelectric hybrid nanogenerator converts biomechanical energy from walking into electrical energy that directly powers ECG acquisition, delivers a 14 ms operating cycle with an energy consumption of 220 μJ, and transmits signals wirelessly to an AI cloud server for near-instantaneous display on a smartphone or computer. This demonstration illustrates how harvesting, power management, and communication can be co-designed as a single operational loop [119] (Fig. 9c).

However, harvesting-driven wearable systems are not limited to biomechanical power sources, and biofluid transport itself can also be exploited as both the energy source and sensing pathway. Zhang et al. fabricated wearable battery-free perspiration-analyzing sites based on ZnO nanowire arrays on a flexible polydimethylsiloxane substrate, where sweat was guided into microchannels by capillary action and flowed along enzyme-modified ZnO nanoarrays to generate a direct-current electrical signal without any external power supply. In this platform, the sweat-induced hydrovoltaic effect established the baseline potential difference, while enzymatic reaction products modulated the electric double layer and thereby altered the output voltage according to lactate concentration, enabling self-powered biochemical sensing in a compact wearable format [130] (Fig. 9d). Here, physiological sampling and power generation are physically coupled, so sweat transport no longer serves only as a delivery process but becomes part of the device operation itself.

At the system level, stable battery-less operation is rarely achieved by energy conversion alone, because wearable power delivery must remain reliable despite intermittency, variable user activity, and changing environments. This motivates architectures that distribute and buffer harvested energy across wearable form factors and select harvesting modalities based on where energy is most consistently available on the body during use [129, 132]. In parallel, harvesting schemes can be paired with sensing functions so that mechanical inputs simultaneously generate energy and provide physiological context. Biofluid-driven and thermal energy-harvesting pathways can offer complementary operation when motion is limited, supporting use across diverse conditions [17, 18, 133]. Once continuous and energy-sustainable operation has been established at the hardware level, the next challenge is no longer how to acquire signals, but how to interpret them robustly despite their variability, dimensionality, and context dependence. This shift leads naturally to the following section, which examines artificial intelligence as the computational layer that extracts reliable meaning from complex wearable data.

## Artificial Intelligence for Soft and Flexible Electronics

With the material, manufacturing, and hardware foundations established in Sects. 2, 3, 4, the discussion now shifts from building reliable data streams to extracting reliable meaning from them. Therefore, this section reviews artificial intelligence as the computational layer that stabilizes imperfect soft-electronic signals, supports multimodal inference, and frames the privacy and security requirements needed for trustworthy deployment.

Artificial intelligence serves as the integrative layer that transforms advances in soft materials and device engineering into deployable, autonomous systems. Importantly, the dominant algorithmic task is dictated by the dominant physical nonideality: motion artifacts increase the denoising burden, hysteresis and creep increase the temporal memory required for compensation, channel density and multimodal fusion determine model dimensionality, and energy scarcity constrains whether inference can remain continuous at the edge. By bridging physical transduction and decision-making, learning-based methods enable these platforms to process complex spatiotemporal signals, manage uncertainty, and operate efficiently on constrained edge hardware. AI is therefore not merely a post-hoc analytical tool but a co-design dimension that must be matched to the signal statistics produced by materials, interfaces, and device architectures [19–24, 134].

Accordingly, a systematic optimization workflow must begin with application-level targets, such as allowable error, latency, and wear duration. These targets are then translated into physical budgets like channel density and power. Hardware and algorithms are subsequently co-selected so that physical designs suppress dominant disturbances at the source, while AI handles residual nonlinearities.

### AI-Driven Signal Enhancement and Compensation for Material Limitations

In soft electronics, artificial intelligence is not merely a supplementary classifier but an essential compensation layer that co-evolves with materials and mechanics to ensure real-world reliability. While soft materials enable conformal sensing, they inevitably introduce variability at the sensor-skin interface due to motion, viscoelasticity, and environmental changes. AI acts as an algorithmic equalizer, absorbing this variability to convert raw, imperfect signals into stable, interpretable data. Without this robust compensation, advanced models would learn device-specific artifacts rather than true physiological intent, severely limiting their clinical usability. Ultimately, computational signal compensation bridges hardware limitations and scalable intelligence, laying the foundation for dependable inference and complex, closed-loop applications [19, 21, 111].

#### Mitigating Motion Artifacts via Adaptive Filtering and Denoising Autoencoders

Motion artifacts remain a primary bottleneck for soft and flexible wearables. The mechanical compliance that enables intimate skin contact simultaneously introduces non-stationary disturbances, including micro-slippage at the electrode–skin interface, resistance changes caused by strain, pressure fluctuations, and triboelectric parasitics. These artifacts severely distort weak biosignals and often dominate the spectrum of interest during daily activities, rendering conventional fixed linear filtering insufficient. To address this challenge, mitigation strategies in soft electronics typically combine adaptive filtering with learned denoisers. Adaptive filtering utilizes reference channels correlated with motion to track dynamic noise, while denoising autoencoders capture nonlinear artifact patterns to reconstruct physiologically plausible waveforms.

Ullah et al. demonstrate a fully integrated motion artifact reduction strategy for electrocardiograms measured by flexible biosensors using two-dimensional convolutional denoising autoencoders [19]. By learning a direct mapping from contaminated segments to cleaned representations, the model compensates for complex activity-related noise without relying on manual heuristics. This approach stabilizes waveform morphology and enables reliable feature extraction under realistic physical movement. Such learned denoising aligns with the broader shift toward direct inference wearables, where dependable preprocessing is a strict prerequisite for advanced interpretation. Robust artifact mitigation directly dictates whether downstream models can generalize beyond controlled laboratory protocols, enabling diverse applications spanning embedded rehabilitation monitoring [24], silent speech decoding [135], respiratory diagnosis [20], and facial expression recognition [136].

Robust signal conditioning serves as the foundational layer that upgrades soft electronics from passive sensing devices to autonomous intelligent systems. It defines the effective operating envelope for more advanced architectures, including multimodal fusion networks, transformers, and graph neural networks. By compensating for material and interface limitations at the signal level, artifact mitigation acts as the gateway to reliable intelligence.

#### Compensating for Hysteresis and Creep: RNN and LSTM Frameworks

Hysteresis and creep are pervasive in soft and stretchable sensors because their transduction layers and interfaces are inherently viscoelastic and microstructurally reconfigurable under deformation. Consequently, identical stimuli can produce varying electrical outputs depending on the loading and unloading paths or the elapsed time under a constant load. This dynamic behavior yields strong history dependence, rate dependence, and prolonged drift. These effects are particularly problematic for wearable strain or pressure sensors and embedded soft robotic skins operating under continuous bending, shear, and relaxation in unconstrained environments. Recurrent neural networks and long short-term memory frameworks are highly suited to address this regime. They learn a stateful mapping from raw sensor streams to corrected estimates by encoding temporal context, effectively capturing the memory of prior deformations. In practice, recurrent perception models stabilize shape and force inference from embedded soft sensors [137] and maintain closed-loop control with dynamic soft sensing [138].

**Fig. 10:**
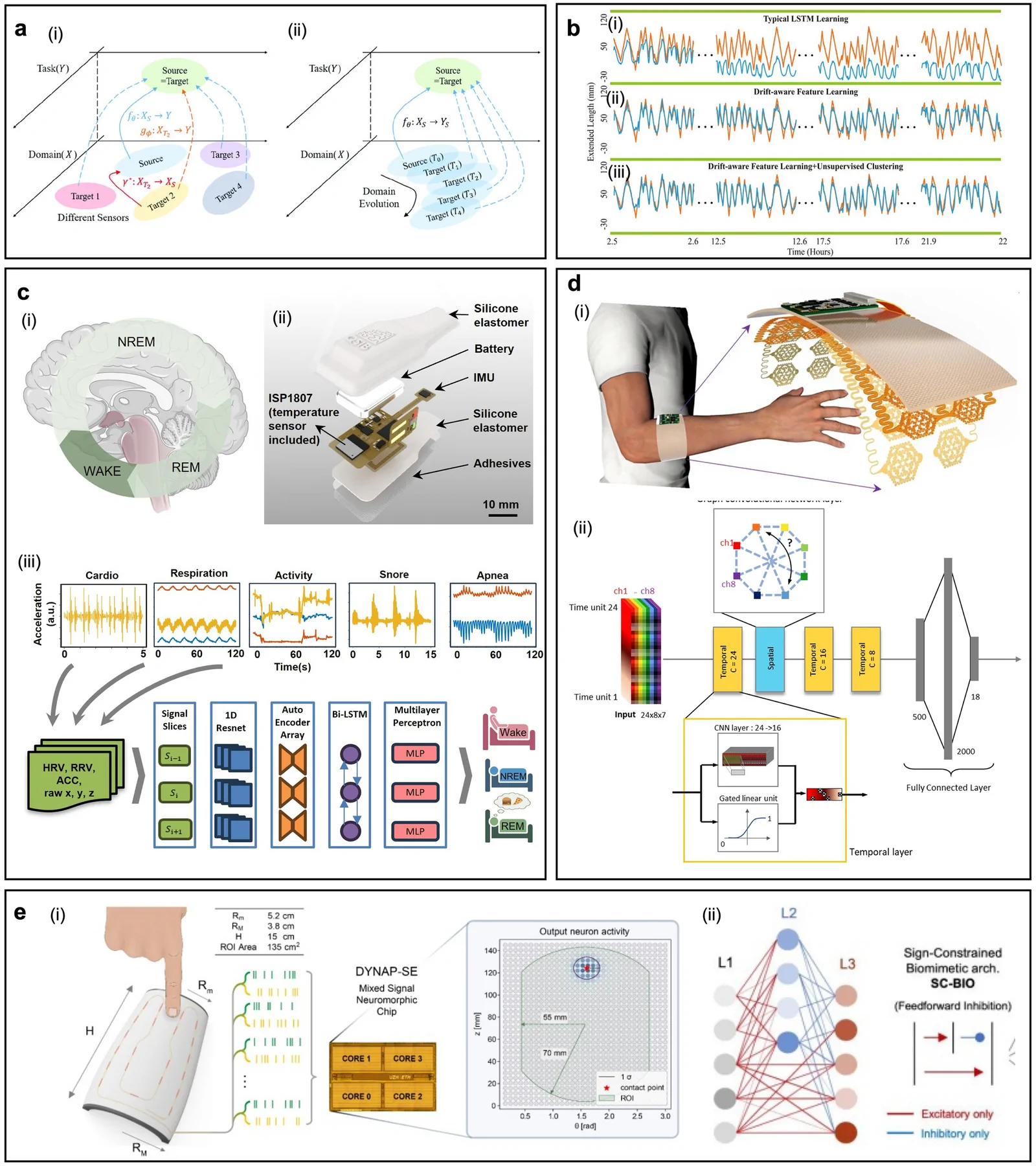
AI-Driven Approaches for Overcoming Material Limitations and Enabling Multimodal Analysis in Soft Electronics. **a** Optimal transport-based domain mappings (γ) for soft sensor calibration, resolving sensor-to-sensor variability through target-to-source mapping and modeling aging-induced long-term drift via successive mappings. Reproduced with permission [111]. Copyright 2020, Wiley–VCH. **b** Comparison of long-term estimation performance in neural networks, contrasting a standard LSTM calibration baseline with improved results achieved via drift-aware feature learning using segmentation and unsupervised clustering. Reproduced with permission [21]. Copyright 2024, Wiley–VCH. **c** System architecture of the neck-mounted LMA wearable and its accompanying signal-processing pipeline, which maps cardiorespiratory and acceleration signals into sleep-stage features for the proposed LMASN model. Reproduced with permission [139]. Copyright 2025, National Academy of Sciences. **d** Overview of a wearable setup in which a stretchable array EMG sensor is integrated with a wireless acquisition module for real-time EMG monitoring and the architecture of the graph attention network. Reproduced with permission [140]. Copyright 2023, Springer Nature. **e** Overview of the touch-encoding system, integrating an FBG-embedded e-skin with a neuromorphic chip, featuring the bioinspired spiking architecture alongside example output-neuron activity. Reproduced with permission [141]. Copyright 2026, Springer Nature

As illustrated in Fig. 10a, Kim et al. proposed an optimal transport-based transfer learning framework designed for the adaptive calibration of soft sensors, effectively addressing variability over extended periods [111]. Rather than treating each sensor as an isolated device requiring complete recalibration, this approach formulates manufacturing variation and drift induced by aging as a distribution shift between a fully calibrated source domain and a target domain evolved over time. Optimal transportation provides a principled method to align these domains by learning a mapping with minimal cost between their output distributions, thereby enabling calibration transfer with limited additional data.

Hysteresis and creep are not merely incidental noise sources but intrinsic dynamical behaviors that fundamentally limit accuracy in downstream tasks, such as tactile contact localization, force estimation [142], and broader interactive sensing scenarios. Explicitly modeling these time-dependent nonlinearities establishes the critical stability required for more complex reasoning architectures. This algorithmic compensation elevates soft electronics from raw sensing to robust inference, paving the way toward learning models that are aware of drift and require no calibration in complex wearable systems.

#### Data-Driven Strategies for Calibration-Free Sensing and Drift Compensation

Data-driven, calibration-free strategies aim to bypass repeated individualized tuning for soft sensors, which are highly susceptible to device-to-device variations and temporal drift. Instead of relying on fixed transfer functions, these models learn invariant representations and align statistical distributions between initial factory measurements and signals acquired in practical environments. To facilitate mass production, methods utilizing domain adaptation and transfer learning can project a calibration model from a limited set of reference sensors to numerous new devices [111]. Within wearable applications, calibration-free algorithms can effectively neutralize confounding variables such as the physical placement of the sensor. For instance, a wristband measuring biological impedance employed a convolutional neural network autoencoder to reconstruct an arterial pulse waveform independent of location, thereby enabling cuffless blood pressure estimation [143].

Wang et al. introduced a feature learning framework for explicit drift recognition [21] (Fig. 10b). By incorporating an autoencoder, the framework compresses historical data into latent representations to effectively identify the core signatures of sensor drift. These features are subsequently utilized as inputs to an LSTM regressor. By treating drift as a learnable state variable, this architecture preserves an accurate mapping even as the physical response of the sensor shifts during extended operation. Crucially, the updating phase leverages prior data alongside only a minimal amount of newly acquired data, significantly reducing the necessity for frequent manual recalibration. When applied to a piezoresistive strain sensor inspired by kirigami structures that exhibits substantial drift over long durations, this drift-aware representation outperforms conventional recurrent baselines.

While earlier discussions emphasize improving instantaneous signal fidelity through artifact removal and modeling short-term nonidealities, such as hysteresis and creep, the fundamental question governing practical adoption remains whether soft sensors can maintain accuracy across diverse users, individual devices, and extended periods of use. From a deployment perspective, manufacturing variation and long-term drift are better interpreted as forms of distribution shift, because they directly alter the data distributions encountered by downstream learning models. However, robust model generalization also requires reference-batch calibration, domain adaptation across fabrication runs, continual updating with small amounts of incoming data, and uncertainty-aware monitoring. Accordingly, standardization at both the device and data levels, including reproducible fabrication windows, stable labeling protocols, and shared evaluation metrics, becomes as important as algorithm design itself [111].

### Advanced Neural Architectures for Multimodal Wearable Data

As wearable platforms evolve into distributed, multimodal systems like electronic skins and smart textiles, the primary bottleneck in soft electronics is shifting from data acquisition to data interpretation. Building on the correction of initial signal imperfections, the core challenge now lies at the systems-inference level. Complex, spatiotemporal signals must be converted into stable, transferable latent states that enable reliable recognition and decision-making across diverse users. While continuous hardware improvements expand the amount of usable information, they also drastically increase data dimensionality and complexity. Ultimately, selecting the right advanced neural architectures determines whether this computational intelligence can operate reliably at the edge with low latency and practical power budgets, paving the way for neuromorphic computing and real-world applications [140, 141, 144–146].

#### Heterogeneous Sensor Fusion using CNNs and Multimodal Models

Heterogeneous sensor fusion is central to intelligent soft wearables because single transducers often provide underdetermined data. Identical electrical changes can arise from genuine physiological signals, physical motion, or contact drift. Consequently, fusing complementary modalities, such as arrays for strain and pressure, inertial measurement unit (IMU) signals, mechanoacoustic vibrations, and bioelectric readouts, creates a joint embedding that is significantly more robust than any individual channel. Convolutional neural network (CNN) encoders are widely used in this context. One-dimensional (1D) convolutional networks learn local waveform motifs in time series data, whereas two-dimensional (2D) variants treat dense sensor arrays as images to capture spatial correlations. Fusion can occur at the signal, feature, or decision level, often incorporating attention or gating mechanisms. This enables weighting based on reliability during physical movement, which is critical for gesture and sign language interfaces in multimodal gloves and electronic skins [61, 144, 147, 148].

As illustrated in Fig. 10c, Du et al. demonstrate how multimodal fusion can elevate a single skin patch toward clinical-grade sleep staging and the detection of sleep disorders [139]. Their wireless and highly efficient mechanoacoustic device, placed at the base of the neck, records triaxial vibrations. These vibrations jointly encode breathing effort, chest motion, and signatures induced by heart sounds while simultaneously capturing gross physical activity. A digital signal processing (DSP) pipeline extracts respiration and cardiac timing to construct a comprehensive feature set, including variables for heart rate variability, respiratory rate variability, and activity features derived from an accelerometer. For the learning phase, the network architecture begins with a one-dimensional residual network acting as a feature extractor on raw triaxial streams. It then fuses these deep features with the engineered DSP outputs through an array block of autoencoders. Subsequently, a bidirectional long short-term memory (LSTM) network models transitions between sleep epochs prior to classification. Evaluated in a clinical cohort including cases of sleep apnea, the framework highlights respiration-derived features as dominant for staging and disorder cues. This illustrates the critical importance of heterogeneous fusion when key biomarkers are only intermittently observable.

#### Transformer-Based Analysis of Time-Series Dependencies

Models utilizing Transformer architectures are increasingly adopted to analyze the lengthy and multichannel time-series data produced by soft and flexible wearables. In these systems, informative patterns can span hundreds to thousands of samples. Unlike recurrent pipelines that compress historical data into a single hidden state, Transformers employ self-attention mechanisms to directly model relationships between distant points in time. This approach is highly advantageous for rate-dependent motion, intermittent contact, and user-specific variability. In practice, sensor streams are tokenized as temporal patches or frames for each sensor, augmented with positional information, and processed by temporal attention blocks. These blocks learn which segments are most predictive for specific tasks, such as estimating joint angles, recognizing gestures, or classifying physiological states. Furthermore, attention mechanisms help reduce the weight of corrupted data windows. However, the computational and memory costs necessitate compact designs, including sparse attention, linear attention, or knowledge distillation, for on-device wearables.

Hu et al. integrated a stretchable electronic skin with a Transformer network to accurately infer the morphology of a soft robot [145]. The primary challenge lies in the fact that channels from the electronic skin provide indirect and spatially distributed observations of a continuous body. Furthermore, the mapping from the signal to the physical shape is highly nonlinear and coupled temporally under dynamic actuation. By learning attention across multichannel sequences, the Transformer integrates cues over a long horizon, understanding how earlier deformations constrain the present configuration. Simultaneously, it remains robust to transient disturbances, enabling high-resolution reconstruction from data generated by soft sensors. This Transformer-assisted perspective is echoed in soft human–machine interfaces, such as triboelectric touch pads employing Transformers to recognize gestures [149], and in lightweight temporal Transformer blocks designed for classifying electrocardiograms at the computing edge [23]. More broadly, as stretchable tactile arrays and printed electrode systems advance toward fine-detailed interactions and the decoding of gestures, modeling temporal context over longer durations becomes increasingly valuable [150].

Transformers exemplify how algorithmic advances can unlock richer information from hardware that is mechanically compliant yet produces highly complex signals. This architecture provides a robust temporal backbone that perfectly complements the fusion of heterogeneous sensors. Moreover, it can be combined seamlessly with spatial inductive biases, such as graph neural networks, when sensor layouts are highly irregular. Moving beyond the mere enhancement and compensation of raw signals, this approach marks a critical shift toward learning transferable representations that support complex recognition and precise structural reconstruction. This evolution ultimately enables context-aware intelligence that operates with minimal latency in subsequent generations of soft wearable systems.

#### Spatiotemporal Sensing with Graph Neural Networks (GNNs)

Graph neural networks (GNNs) are highly suited to spatiotemporal sensing in soft electronics. Many wearable platforms and electronic skins generate spatially coupled signals that lack a regular grid arrangement. Each sensing element, such as an electrode or a tactile pixel, is modeled as a node. Edges encode physical neighborhoods, wiring structures, or learned correlations. Through message passing, information aggregates across neighbors to form spatial features that are subsequently paired with temporal encoders. This formulation naturally accommodates irregular layouts, physical deformation, and missing channels. For tactile perception, models utilizing graph attention explicitly exploit structural relationships between tactile pixels [151]. Beyond basic classification, deep-learning-driven tactile resolution enhancement [152] and the simultaneous design of sensors and algorithms [153] emphasize that learning relationships among sparse nodes can recover rich spatial information.

As illustrated in Fig. 10d, Lee et al. highlight a stretchable array system for surface electromyography (sEMG) integrated with a graph neural network utilizing self-attention to recognize static and dynamic gestures [140]. The platform employs an expansive array of bipolar stretchable electrodes positioned to cover forearm muscles. This array is supported by an adhesive perforated patch that improves conformal contact during motion and extended wear. Algorithmically, self-attention constructs an input-adaptive adjacency graph, ensuring that edge weights reflect which pairs of electrodes are most informative for a given gesture segment. Temporal dynamics are subsequently learned on top of these spatial representations. The system reports an accuracy of approximately 97% across 18 gestures, even when trained with only a single trial per gesture. Furthermore, it maintains approximately 95% accuracy after more than 72 h of wear and 10 reuse cycles, highlighting its exceptional robustness against alignment noise and motion-dependent artifacts.

Graph neural networks provide a geometry-aware counterpart to the fusion of multimodal sensors and the learning of long sequences. By treating sensor topology as a primary input, these networks establish a robust foundation for spatial reasoning. As soft systems scale toward dense arrays and distributed sensing skins, reliability heavily depends on exploiting structured interdependence across channels under conditions of deformation, partial delamination, or variations in placement. Consequently, spatiotemporal learning utilizing graphs underpins reliable human–machine interfaces (HMIs) with high degrees of freedom and advanced tactile intelligence. This paradigm naturally connects to highly efficient neuromorphic architectures and event-driven inference methods, paving the way for the next generation of intelligent soft electronics.

#### Energy-Efficient Event-Driven Processing via Spiking Neural Networks

Spiking neural networks (SNNs) offer a highly efficient, event-driven alternative to traditional frame-based deep learning. This paradigm is crucial for soft wearables that must operate continuously under strict power and latency constraints. Instead of streaming dense signals at fixed sampling rates, these pipelines encode information as sparse spikes. Neural states update only when informative events occur, significantly reducing data movement and enabling millisecond-scale responsiveness. This rapid response is particularly valuable for capturing tactile transients, slip-like microdynamics, and the rapid localization of contact. In this context, the encoding scheme becomes as critical as the classification itself. The timing of spikes, such as the time elapsed before the first spike, can compactly represent fast touch dynamics for recognizing moving objects [154]. Simultaneously, concepts inspired by the peripheral nervous system motivate scalable and distributed electronic skins that locally convert analog physical deformation into spike-based representations [155].

To enable large-area tactile intelligence, Ortone et al. addressed critical practical barriers, including excessive wiring, high energy consumption in conventional AI, and limited scalability [141], as shown in Fig. 10e. The researchers integrated an electronic skin utilizing fiber Bragg gratings with an entirely event-driven spiking neural network to emulate early somatosensory processing. Notably, this architecture achieves up to a tenfold enhancement in spatial resolution and reports a 32% improvement in localization accuracy compared with advanced deep learning baselines. Furthermore, it generalizes effectively to multiple simultaneous touches and dynamic physical conditions. Crucially for deployment in wearables and robotics, the network is implemented on a mixed-signal neuromorphic chip. It maintains high decoding performance despite constrained analog resolution and inherent physical device-to-device mismatches, enabling highly parallel computations requiring < 1 mW of power. Beyond engineering metrics, this platform probes how biologically-inspired inhibitory connectivity motifs functionally shape tactile decoding, successfully linking physiological hypotheses to practical tactile validation.

CNNs and multimodal fusion models are highly effective for localized feature extraction and integrating heterogeneous data streams, making them ideal for multi-sensor health patches. Transformers excel at modeling long-horizon temporal dependencies and managing intermittent contact noise, which is critical for continuous dynamic shape reconstruction and gesture decoding. GNNs are uniquely suited for spatially distributed, irregular sensor networks, effectively handling the shifting topologies of large-area electronic skins and stretchable electrode arrays. Finally, SNNs provide the optimal architecture for ultra-low-latency, power-constrained edge applications, particularly in capturing rapid tactile transients and event-driven interactions where continuous digitization is impractical.

### Data Privacy, Security, and Trustworthy Deployment

Intelligent soft systems are evolving from passive data collectors into continuously operating platforms capable of clinically relevant inference and long-duration use [63, 90, 134]. Consequently, data privacy and security must be treated as system-level design constraints rather than downstream software afterthoughts. Unlike episodic measurements, wearable soft electronics capture dense, longitudinal streams of electrophysiology, hemodynamics, motion, and contextual data. These multimodal streams can inadvertently expose sensitive health and behavioral information extending far beyond the original sensing task. Compounding this issue, nominal deidentification does not guarantee protection against reidentification risks. Trustworthy deployment, therefore, demands a privacy-by-design approach across the entire sensing pipeline, spanning the skin interface, wireless relays, and the training, updating, and storage of models.

At the device and network levels, wearable health systems are exposed through body-area communication networks, short-range wireless pairing, smartphone or cloud relays, and firmware updates. These touchpoints create vectors for unauthorized access, spoofing, replay attacks, data tampering, and leakage of sensitive physiological or location data. Because soft wearables operate under strict battery, memory, and compute constraints, they cannot simply adopt the heavyweight security stacks used in conventional mobile electronics. Practical protection therefore requires lightweight encryption, mutually authenticated key establishment, secure storage and transmission, safe firmware-update pathways, and auditable access controls across the sensor-edge-cloud continuum [156].

At the algorithmic layer, on-device inference reduces exposure of raw data but does not by itself guarantee privacy. Privacy-preserving AI is increasingly important because collaborative learning pipelines remain vulnerable to model inversion, membership inference, and source inference attacks even when raw signals stay on the local device [157–160]. Federated learning is attractive for soft electronics because it keeps raw physiological data local while still allowing cross-user or cross-site model enhancements [158, 159]. It can be strengthened further with differential privacy, secure aggregation, and, where justified, additional cryptographic protection [157, 159, 160]. These safeguards, however, introduce trade-offs in communication cost, latency, update stability, and energy use. AI-integrated soft electronics should therefore be evaluated not only by inference accuracy and power efficiency, but also by privacy loss, attack robustness, data-retention policy, and user-governed control over data sharing and model updates.

Taken together, this section shows that artificial intelligence supports soft electronics at three connected levels: compensating for artifacts, hysteresis, and drift; learning richer multimodal and spatiotemporal representations; and enabling privacy- and security-aware deployment. These advances turn variable soft-sensor outputs into more reliable and transferable system decisions, but their practical impact depends on hardware that can execute them within strict energy and bandwidth limits. This requirement motivates the neuromorphic and in-sensor computing strategies discussed in the next section.

## Neuromorphic and In-Sensor Computing: Material-Level Intelligence

While Sect. 5 demonstrated how artificial intelligence serves as an essential algorithmic stabilization layer for interpreting complex, high-dimensional soft sensor data, executing these advanced models on conventional computing architectures frequently introduces severe energy and bandwidth bottlenecks. To overcome these real-world constraints, the computational paradigm must shift from software-based post-processing toward hardware-level intelligence. In this context, neuromorphic and in-sensor computing act as the direct physical embodiment of the AI principles discussed previously, translating algorithmic intelligence into material intelligence. This represents a major shift: moving away from reliance on external AI and instead embedding intelligence directly within the sensing material. As soft electronics evolve into dense, always-on networks, the main challenge is no longer algorithmic accuracy, but rather sustaining continuous operation under strict energy, bandwidth, and memory constraints. Because constant data digitization and wireless streaming are highly inefficient in these scenarios, neuromorphic principles like statefulness, locality, and analog computation provide a vital hardware-based solution. This approach minimizes data movement and enables real-time, closed-loop responses directly at the sensor interface.

**Fig. 11:**
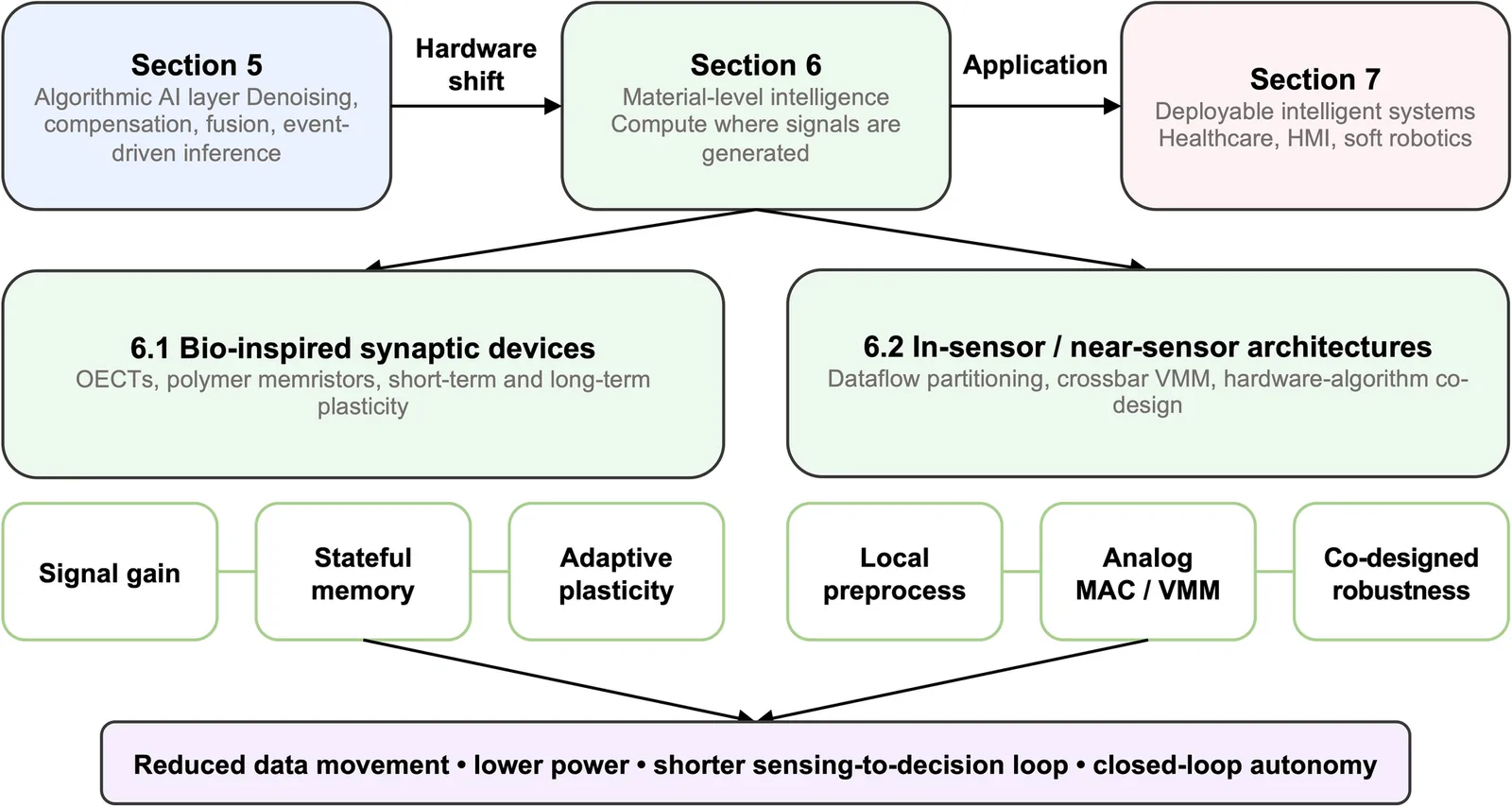
Conceptual framework of neuromorphic and in-sensor computing in intelligent soft electronics. This schematic illustrates the transition from algorithmic AI (Sect. 5) to material-level intelligence (Sect. 6), where computation occurs directly at the signal source. By integrating bio-inspired synaptic devices (Sect. 6.1) with in-/near-sensor architectures (Sect. 6.2), the framework provides essential capabilities like stateful memory, analog MAC/VMM, and local preprocessing. This integration significantly reduces data movement and power consumption, enabling closed-loop autonomy for deployable intelligent systems in healthcare, human–machine interaction, and soft robotics (Sect. 7)

As summarized in Fig. 11, Sect. 6 is organized around two coupled layers: device-level synaptic material primitives (Sect. 6.1) and architecture-level in-sensor/near-sensor computing strategies (Sect. 6.2).

### Bio-Inspired Synaptic Devices and Material Intelligence

Bio-inspired synaptic devices provide the physical foundation for embedding intelligence directly into soft materials, bypassing the reliance on external artificial intelligence. While algorithms can compensate for imperfect signals, a critical hardware bottleneck remains. For continuous wearables and electronic skins, the traditional von Neumann separation of sensing, memory, and computing creates severe limitations in power, latency, and data movement. Synaptic devices overcome these issues by functioning as building blocks that seamlessly merge signal amplification, memory, and adaptive dynamics within physically compliant forms. This native integration allows for local preprocessing, on-body personalization, and real-time adaptation exactly where signals are generated. Ultimately, by combining soft material engineering with neuromorphic principles like plasticity and analog computation, these devices lay the groundwork for advanced in-sensor architectures and scalable intelligent systems [31, 161–165].

#### Organic Electrochemical Transistors for Signal Amplification and Synaptic Emulation

Organic electrochemical transistors (OECTs) constitute a core hardware primitive for material intelligence in soft bioelectronics. This functionality arises because electrolyte gating couples ionic motion directly to electronic transport within an organic channel. The mixed ionic-electronic transduction yields remarkably high transconductance at low operational voltages in hydrated environments highly compatible with human skin. Simultaneously, ion kinetics provide tunable temporal dynamics. The ion-mediated volumetric doping and dedoping provide signal gain while introducing inherent statefulness, which serves effectively as analog memory and synaptic plasticity. Consequently, these transistors function simultaneously as localized amplifiers and neuromorphic elements. This dual capability enables stretchable wearable computing platforms embedded directly within sensors [166] and fully integrated flexible circuits [167].

**Fig. 12:**
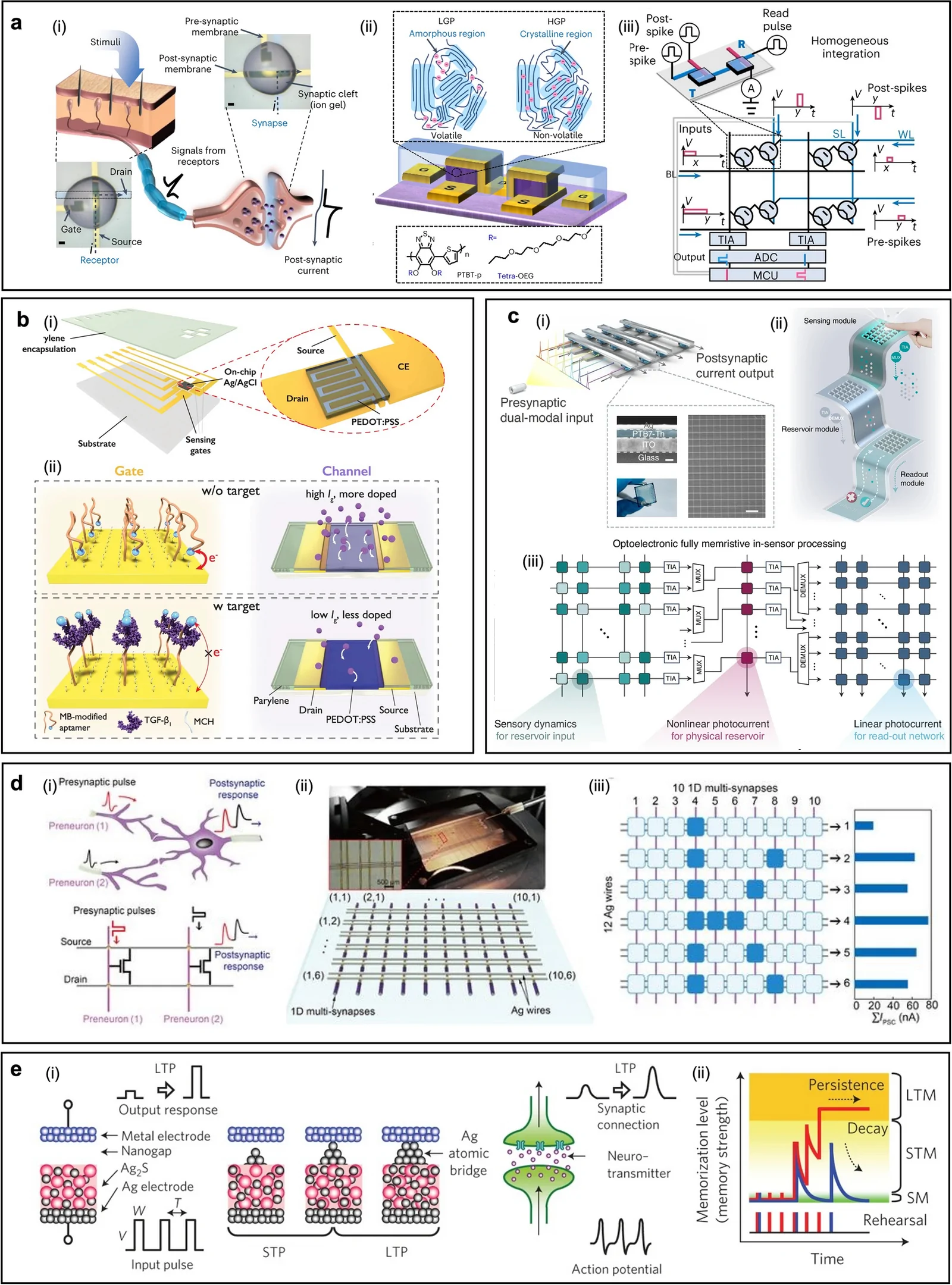
Emulation of Biological Neural Networks via Organic Electrochemical Transistors and Polymer Memristors. **a** Comparison of biological neural function with a cv-OECT-based artificial nervous system, illustrating the device’s dual operation as a volatile receptor and non-volatile synapse and its integration into a homogeneous spiking neural network architecture. Reproduced with permission [31]. Copyright 2023, Springer Nature. **b** Schematic and detection principle of a reference-OECT-integrated electrochemical aptamer-based sensor for TGF-*β*1, where target binding induces an aptamer conformational change that distances the methylene blue reporter from the gate. Reproduced with permission [168]. Copyright 2023, Springer Nature. **c** Design and morphological characterization of a fabricated optoelectronic memristor crossbar array utilized within an analog, fully memristive in-sensor reservoir computing system. Reproduced with permission [30]. Copyright 2025, Springer Nature. **d** Biological and artificial signal transmission comparison using a NOR-type synaptic array, combined with array implementation demonstrating pattern encoding via LTP/LTD states and row-wise IPSC readouts. Reproduced with permission [161]. Copyright 2020, American Association for the Advancement of Science (AAAS). **e** Comparison of an Ag_2_S inorganic synapse and a biological system, illustrating a multistore memory model where repeated stimuli form Ag atomic bridges to consolidate short-term into long-term memory. Reproduced with permission [169]. Copyright 2011, Springer Nature

As shown in Fig. 12a, Wang et al. proposed a multifunctional architecture that combines multimodal sensing with programmable conductance states for memory. This integration enables local preprocessing and synaptic weight updates within a single device [31]. The paradigm of sensing, storing, and computing simultaneously perfectly suits soft wearables constrained by strict power and bandwidth limits. It facilitates compact analog interfaces and simple computational blocks utilizing gel-gated stretchable layouts. In parallel, as presented in Fig. 12b, Ji et al. utilized these transistors as localized transconductance amplifiers for electrochemical aptamer sensors. This configuration boosts minute biochemical signals directly at the physical interface, thereby simplifying the requirements for external readout electronics [168].

Organic electrochemical transistors emerge as a unifying device class that closes the loop between soft physical sensing and embedded computation. By co-locating signal gain, memory retention, and adaptive ionic dynamics within a mechanically compliant form factor, these devices establish a critical foundation for advanced synaptic materials and in-sensor or near-sensor computational strategies. This device-centric perspective naturally extends to embodied demonstrations of continuous closed-loop operation. For instance, organic neuromorphic systems have successfully enabled the integration of sensory and motor functions for robotic learning [170] alongside multimodal behavioral conditioning inspired by biological systems [171].

However, we argue that the current literature often overemphasizes isolated device performance while neglecting the practical challenges of dynamic wear. The critical bottleneck for deploying OECTs is managing the inherent variability of ion kinetics under continuous mechanical deformation and fluctuating thermal environments. To transition from proof-of-concept to scalable intelligent systems, future research must prioritize stabilizing these temporal dynamics and tightly integrating them into robust, multi-device architectures capable of operating reliably on human skin.

#### Polymer-Based Memristive Switching for Non-Volatile Memory

Polymer memristors are most valuable to soft neuromorphic systems as distributed nonvolatile memory elements rather than as simple flexible analogues of rigid oxide memories. Their appeal lies in low-temperature processing, compatibility with solution-based fabrication, and mechanical similarity to textiles and elastomers, which make them naturally suited to large-area and wearable platforms. These advantages are especially important when learned weights must be retained locally without relying on rigid off-device memory. The main challenge, however, is no longer whether switching can be demonstrated, but whether analog conductance updates are sufficiently linear, stable, and reproducible for useful computing. Retention, cycle-to-cycle variation, and device-to-device mismatch remain critical bottlenecks even when multilevel states are available. Material strategies range from trapping interfacial charges and ionic or ferroelectric polarization to forming filamentary pathways, as exemplified by polymer analog synapses featuring atomic-scale conductive filaments [162] and fluoropolymer-based organic memristors for multifunctional flexible neural hardware [172].

Zhou et al. reported polymer memristors where programmable conductance dynamically tunes under combined electrical and optical stimuli. This dual modulation enables adaptable weight control and highly energy-efficient near-sensor processing [30] (Fig. 12c). This profound controllability aligns perfectly with reservoir computing embedded within wearable sensors utilizing optoelectronic polymers. Here, rich device dynamics facilitate learning multiple tasks without the constant need to offload raw data [173]. Complementarily, as shown in Fig. 12d, Ham et al. demonstrated fiber-shaped synaptic elements that are entirely compatible with textiles. These elements weave directly into fabrics, integrating persistent synapses into garments for wearable neuromorphic operations [161].

Polymer memristors therefore provide the persistence that purely dynamic synaptic devices often lack. Stable, analog, nonvolatile conductance states allow learned representations to survive deformation and long-term use, which is important for on-body personalization and autonomous operation. From a systems perspective, however, persistence alone is insufficient; the central challenge is whether that memory can be updated with enough precision under the nonideal conditions imposed by soft substrates, distributed wiring, and lightweight peripheral circuits. This issue is underscored by recent work on self-healing neuromorphic elements for decentralized sensory processing [163] and flexible neural networks based on organic memristors with biologically realistic plasticity [174]. In this sense, memristive switching in polymers is not merely a physical phenomenon, but a functional capability for next-generation intelligent soft electronics.

#### Emulation of Synaptic Plasticity: From Short-Term to Long-Term Potentiation

Emulating synaptic plasticity is meaningful in soft electronics not simply because it mimics biology, but because it offers a physical way to separate fast adaptation from durable memory. Short-term plasticity (STP) is useful when recent stimulus history should transiently shape the response, for example by filtering isolated noise or encoding local tactile context. Long-term potentiation (LTP), by contrast, becomes valuable when calibration, user-specific behavior, or task memories must persist over longer intervals. The central design question is therefore not whether a device reproduces every biological feature, but whether its intrinsic time scales align with the sensing task. Engineers pursue this separation using fast relaxational processes such as ionic accumulation or capacitive gating together with more stable state changes involving redox reactions, filament growth, or deep charge trapping. Mixed ionic-electronic conductors are particularly attractive for soft electronics because they enable ion-mediated spiking in organic electrochemical neurons and synapses [175] alongside multimodal architectures that unify sensing, memory, and processing [31].

As illustrated in Fig. 12e, Ohno et al. developed a single inorganic synapse capable of reproducing both STP and LTP within a unitary element [169]. Implemented as an Ag_2_S atomic switch device, its conductance modulates through the electrically driven formation and relaxation of a nanoscale conductive pathway. This mechanism makes the synaptic weight a direct function of pulse timing and repetition. Under sparse stimulation, the conductance enhancement relaxes toward the baseline, yielding a fading trace resembling STP that naturally filters isolated events. In contrast, repeated or closely spaced pulses progressively reinforce the conductive state, resulting in potentiation with extended longevity analogous to long-term memory. By mapping input statistics to retention time, this work established a physical consolidation rule where repetition stabilizes memory. This principle is directly relevant to soft sensors, where transient bursts of stimuli can be compressed into durable features for local learning and adaptation.

Many demonstrations can qualitatively show STP or LTP, but the more important benchmark for soft electronics is whether these dynamics actually reduce recalibration, suppress transient artifacts, or improve closed-loop adaptation under deformation and long-term wear. From this perspective, plasticity is valuable when it maps cleanly onto system needs: STP for transient filtering and context encoding, and LTP for persistent personalization and task retention. This interpretation directly motivates algorithm-device co-design for in-sensor or near-sensor computing architectures, where the goal is not biological imitation itself but energy-efficient autonomy in real operating environments.

### Architectures for In-Sensor and Near-Sensor Computing

At the architectural level, the focus shifts from individual synaptic devices to organized computing hierarchies capable of meeting the real-world constraints of distributed soft sensor networks. These systems must operate under tight power budgets and strict latency requirements for closed-loop interaction, where continuous digitization and wireless streaming are often impractical. By co-locating computation, memory, and communication directly around soft transducers, intelligence emerges at the physical interface rather than in a remote processor. This architectural strategy reduces data movement, tolerates device variability, and supports scalable integration, serving as the essential translation layer between sensing materials and system-level autonomy. Ultimately, these principles bridge the gap between high-level AI models and hardware-realizable implementations, converting algorithmic intelligence into energy-efficient, mechanically compliant, and application-ready soft systems [165, 166, 176–178].

#### Beyond the Von Neumann Bottleneck: In-Sensor versus Near-Sensor Processing

Beyond the von Neumann architecture, the dominant cost in wearable intelligence is frequently the movement of data. Continuously digitizing, transmitting, and storing high-rate streams from soft sensors can easily exceed the available power budget and introduce latency that compromises closed-loop operations. Consequently, two distinct strategies emerge to address this challenge. In-sensor computing embeds memory and primitive computation directly into the transduction element, ensuring that sensing and initial processing occur simultaneously within the same physical material or device. In this case, physical conductance or ionic state serves simultaneously as both the acquired signal and the computational state, so latency and bandwidth are minimized at the point of generation. By contrast, near-sensor computing keeps sensing and computation physically co-located but modular, using compact analog, mixed-signal, or lightweight digital front ends positioned immediately next to the sensor to extract features before higher-level inference.

Across key system dimensions, in-sensor processing is highly favorable for ultralow-latency, ultralow-power operations that require fine-grained local computation, such as temporal integration, thresholding, adaptive weighting, or event encoding. By transmitting only compressed state changes or task-relevant events, this approach drastically minimizes bandwidth demands and keeps data flow strictly local. Such efficiency makes it ideal for always-on wearables, reflex-like closed-loop control, and distributed electronic skins operating under severe wireless or memory constraints.

In contrast, near-sensor processing trades slightly higher latency and power consumption for coarser computing granularity. This trade-off enables shared computation across multiple channels, straightforward multimodal fusion, and easier integration with peripheral circuits and reprogrammable inference hardware. Its data flow relies on a short local pipeline that progresses from transduction and signal conditioning to feature extraction and downstream inference. Consequently, this architecture is especially suited for high-channel-count patches, multimodal interfaces, and adaptive systems that demand regular calibration, algorithm updates, or complex decision logic [166, 167, 173, 176, 179]. In our view, many near-term wearable platforms will favor this route because it retains most of the energy benefit of locality without completely sacrificing design flexibility.

**Fig. 13:**
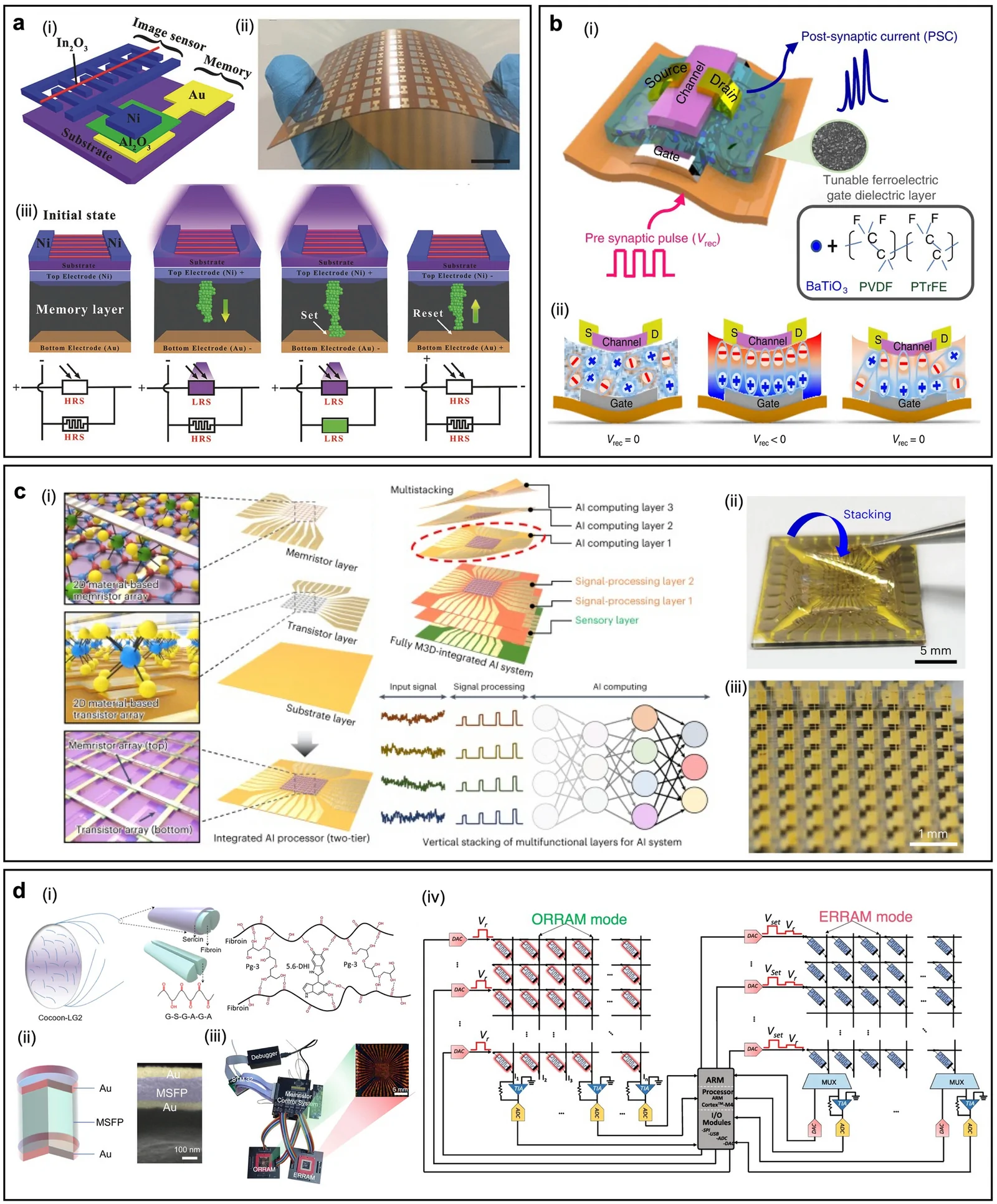
Hardware-Algorithm Co-Design Strategies for Efficient Neuromorphic Interfaces and Sensor-Level Processing. **a** A bioinspired visual memory unit integrating an image sensor with a resistive-switching memory element, featuring photographs of fabricated arrays on rigid SiO_2_ and flexible polyimide substrates alongside its underlying resistive-switching mechanism. Reproduced with permission [180]. Copyright 2018, Wiley–VCH. **b** Schematic and operating mechanism of a flexible intrinsic-synaptic tactile device, demonstrating postsynaptic current generation via ferroelectric dipole polarization. Reproduced with permission [181]. Copyright 2020, Springer Nature. **c** Schematic and morphological views of an edge-computing platform enabled by monolithic 3D integration of 2D materials, featuring stacked sensory, signal-processing, and parallel AI-computing layers. Reproduced with permission [182]. Copyright 2023, Springer Nature. **d** Overview of an MSFP-based multimodal resistive memory device, detailing its cross-sectional FE-SEM and modified silk fibroin protein structure, alongside the architecture and photograph of a fully hardware neuromorphic vision system. Reproduced with permission [183]. Copyright 2023, Springer Nature

As shown in Fig. 13a, Chen et al. effectively demonstrated these principles through an ultraviolet-light-responsive flexible memristor, which serves as the foundation for an artificial visual memory system [180]. Here, optically driven conductance modulation couples photodetection with analog and nonvolatile storage within a single flexible element. Rather than streaming full image arrays, the history of illumination is accumulated locally, enabling event-driven encoding and reducing redundant data readout. Lee et al. applied a related idea to touch with a flexible artificial tactile sensory organ that converts tactile stimuli into synapse-like outputs with inherent plasticity [181] (Fig. 13b).

The continuum of in-sensor and near-sensor processing is now populated by highly stretchable wearable platforms and gel-gated fully integrated circuits that embed analog preprocessing and simple learning directly into compliant hardware. Simultaneously, in-sensor reservoir computing utilizing optoelectronic polymers exploits intrinsic material dynamics for complex temporal inference [173]. Furthermore, primitive multimodal hardware natively emerges through flexible-memristor-based multimodal neurons [179], alongside in-sensor capacitive computing schemes that compute directly during data acquisition [176]. Taken together, these studies suggest that hybrid partitions, rather than purely in-sensor or purely external computation, are likely to dominate practical soft systems in the near term [184].

#### Analog Matrix Multiplication via Resistive Crossbar Arrays

Analog matrix multiplication within resistive crossbar arrays serves as a core engine for in-memory computing for neuromorphic systems and edge artificial intelligence. Programmable conductances at each junction between word lines and bit lines map a specific weight matrix. Applying an input vector as voltages yields column currents that effectively realize vector–matrix multiplication through Ohm’s law and Kirchhoff’s current summation. However, practical scaling faces challenges from sneak paths, voltage drops, inherent variability, limited precision, and signal disturbance. For flexible electronics, transferring these crossbar concepts to polymeric and organic memristors is highly attractive to achieve mechanical compliance and compatibility with printing. Nevertheless, this transition significantly increases demands on structural uniformity and the integration of peripheral components [185, 186].

In Fig. 13c, Kang et al. demonstrated the monolithic three-dimensional integration of electronics based on two-dimensional materials [182]. This approach is critical because the energy consumption and latency of crossbar accelerators are frequently dominated by complex wiring and peripheral circuits, such as drivers and analog-to-digital converters, rather than the analog multiplication process itself. Stacking these components monolithically with atomically thin channels and dielectrics offers a promising route to vertically integrate analog sensing interfaces, memory and computational layers, and control electronics. This strategy drastically shortens interconnects while substantially increasing computational density. This architectural paradigm suggests highly scalable fabrics utilizing multiple layered crossbars with tightly coupled peripherals. Such designs enable larger effective matrices and facilitate on-device adaptation, including efficient local learning within complex memristor networks [187].

Analog matrix multiplication is thus the hardware engine that converts synaptic devices from isolated memory elements into usable inference substrates. Its importance lies in exposing where the real system bottlenecks move once computation enters the material itself: often from multiply-accumulate operations to precision control, peripheral overhead, and error management. This is especially consequential for soft electronics, where mechanical compliance, distributed wiring, and low-temperature fabrication place additional pressure on uniformity and circuit integration. For that reason, the most meaningful progress is not simply larger crossbars, but crossbars whose analog efficiency survives the realities of soft-system packaging, calibration, and long-term use.

#### Algorithm-Hardware Co-Design for Efficient Neuromorphic Interfaces

Hardware-algorithm co-design addresses a recurring limitation in neuromorphic interfaces, specifically that device nonidealities cannot be treated as afterthoughts. The accuracy of resistive in-memory computing is inseparable from stochastic switching, nonlinear and asymmetric updates, array parasitics, and peripheral overhead. Instead of viewing hardware as a fixed backend, co-design deliberately couples learning rules, weight mapping, programming protocols, and interface circuits to the statistical behavior of the physical platform. From our perspective, this is the most realistic path forward for soft neuromorphic systems, because materials improvement alone is unlikely to eliminate the variability introduced by compliant substrates, distributed interconnects, and low-power operation [188, 189].

Hardware-aware retraining methods further embed realistic crossbar models during optimization. This approach recovers accuracy comparable to software levels even under practical nonideal conditions [178]. In parallel, hardware-native learning rules explicitly target analog crossbars to alleviate mismatches typically associated with traditional backpropagation [190]. Complementary strategies address precision and variability at the programming level. These include protocols for high precision programming that explicitly compensate for errors [191] and noise-informed probabilistic weight updates that reconcile stochastic changes in conductance with gradient-driven learning [192].

As presented in Fig. 13d, Zhou et al. demonstrated a complete neuromorphic visual system implemented entirely in hardware [183]. This system utilizes the multifunctional nature of devices to partition computation across a retina-and-cortex-inspired hierarchy. The researchers developed an array utilizing modified silk fibroin protein resistive memory that supports two distinct operational modes. The first is an optoelectronic mode featuring positive and negative photoconductance memory. The second is an electrical mode featuring analog resistive switching. Two physically identical arrays operate in entirely different roles. The optoelectronic front interface performs in-sensor preprocessing. This includes enhancing contrast, reducing noise, and extracting features through convolution-like operations. Meanwhile, the electrical back interface executes advanced recognition tasks in a near-sensor configuration.

By explicitly incorporating device statistics and circuit constraints into training and task partitioning, co-design can preserve accuracy despite physical variation while also reducing latency from sensing to decision. The same logic aligns with decentralized and fault-tolerant sensory nodes, including self-healing neuromorphic memtransistors for robotic skins [163]. More broadly, Sect. 6 suggests the clear design lesson that the most promising soft neuromorphic platforms will not be those that mimic biology most literally, but those that co-optimize local computation, mechanical compliance, calibration robustness, and scalable integration.

## System-Level Applications of AI-Integrated Soft Electronics

With the material, algorithmic, and neuromorphic foundations in place, Sect. 7 turns to how these advances perform in complete systems. It reviews applications in healthcare, human–machine interfaces, and soft robotics, emphasizing robustness, autonomy, and translational relevance.

At the application level, the key question is no longer whether soft electronics can sense or compute in isolation, but whether complete systems can operate robustly during real use. Clinical patches, immersive HMIs, and soft-robotic skins all require stable interfaces, reliable interpretation under distribution shift, and acceptable power, latency, and comfort. This section therefore focuses on system-level performance, with emphasis on robustness, autonomy, and translational relevance rather than peak laboratory accuracy.

A critical limitation in current applications is that high AI performance in controlled settings rarely translates to robust everyday operation. In real-world environments, factors like motion artifacts, sweat, and sensor displacement alter signal distributions. Because soft sensors couple mechanics and transduction, models often misinterpret these environmental perturbations as actual changes in user intent or state. Future research must adopt perturbation-aware validation protocols to test realistic wear conditions. This involves incorporating key benchmarks such as cross-session and cross-user generalization, tolerance to degraded channels and repeated attachments, as well as stability and minimal calibration during unconstrained movement [24, 33, 90].

Beyond nominal AI accuracy, the translational maturity of AI-integrated soft electronics should be judged using application-level metrics. For skin-interfaced healthcare and HMIs, particularly informative criteria include wearing comfort and interface quality, stability/robustness, and operational duration/autonomy. For soft robots and embodied e-skins, analogous maturity indicators include mechanical robustness, control stability under perturbation, load tolerance, and sustained autonomous operation [33, 90, 193–198]. Representative examples and quantitative metrics are summarized in Table 3.

### AI-Driven Personalized Healthcare and Closed-Loop Therapeutics

Personalized healthcare and closed-loop therapeutics mark the transition from foundational technology to real-world clinical impact. While soft electronics enable comfortable and continuous monitoring, their true value is realized only when raw data is transformed into actionable medical insights despite challenges like noise and user variability. AI serves as the essential bridge in this process, personalizing data interpretation and fusing diverse physiological signals into a coherent health context. By turning these insights into adaptive treatments, AI evolves soft platforms from passive sensors into fully autonomous medical systems. Ultimately, these clinical objectives demonstrate that technical advances in signal processing and energy-efficient intelligence are not merely academic exercises; they are vital requirements for creating reliable, large-scale healthcare wearables [63, 90, 194].

#### Real-Time Anomaly Detection and Digital Biomarker Profiling

Real-time anomaly detection and the profiling of digital biomarkers translate continuous streams from wearables into clinically meaningful events and personalized trajectories. Digital biomarkers encompass algorithm-derived features, including fundamental rhythms, morphology, variability, cross-modal patterns, and learned embeddings. These features correlate strongly with physiological states and can be securely tracked against an individual baseline to flag deviations at an early stage. In skin-interfaced soft systems, this process demands exceptional robustness against physical motion, contact drift, and inherent inter-user variability. Consequently, this demand strongly motivates the adoption of representation learning, including self-supervised or semi-supervised methods, alongside uncertainty-aware classification architectures. The ultimate aim is to transition from episodic clinical snapshots to context-aware continuous diagnostics, providing critical alerts with minimal latency [90, 199].

**Fig. 14:**
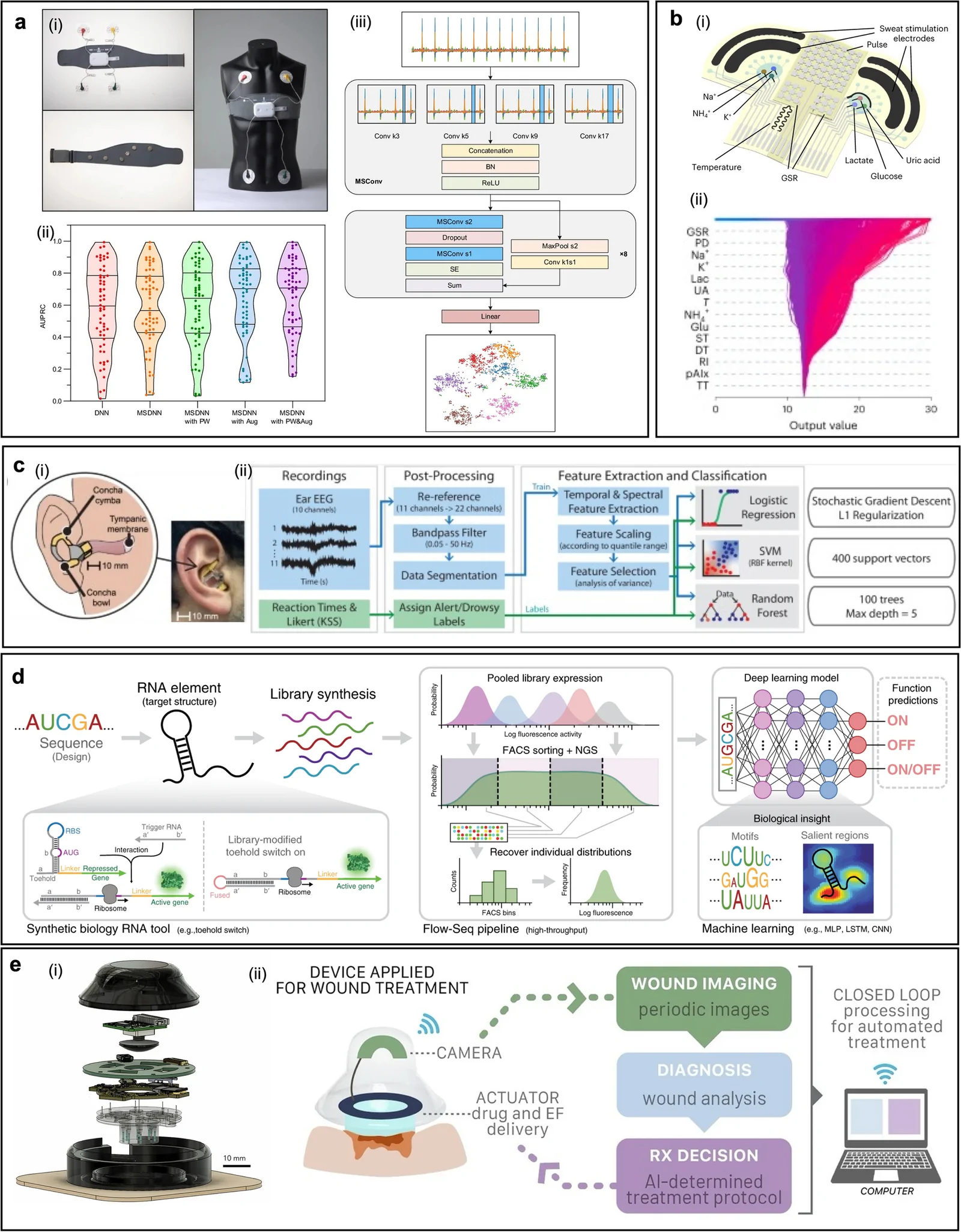
AI-integrated soft electronics for closed-loop personalized healthcare systems. **a** Overview of a wearable 12-lead ECG device and standard electrode placement highlighting real-world artifacts from electrode displacement and weak skin contact, and the architecture of the proposed multiscale convolutional network. Reproduced with permission [200]. Copyright 2023, Springer Nature. **b** Schematic of the flexible e-skin patch highlighting its integrated functions for vital-sign tracking and sweat-based sensing, alongside a decision plot demonstrating how a machine-learning model combines these physiological and biochemical markers. Reproduced with permission [201]. Copyright 2024, Springer Nature. **c** Electrode placement and signal processing pipeline for ear ExG recordings, followed by re-referencing, filtering, and motion artifact removal prior to feature extraction and model training. Reproduced with permission [193]. Copyright 2024, Springer Nature. **d** End-to-end design workflow for programmable RNA switches, demonstrating a toehold-switch model that combines switch/trigger architecture, pooled flow-seq measurements, and DNN-based functional prediction. Reproduced with permission [34]. Copyright 2020, Springer Nature. **e** CAD rendering of the wearable heal platform for adaptive bioelectronic wound therapy. Reproduced with permission [195]. Copyright 2025, Springer Nature

As shown in Fig. 14a, Lai et al. introduced a practical diagnostic framework for wearable 12-lead electrocardiograms (ECGs) based on self-supervised pretraining and task-specific data augmentations [200]. The approach leverages large unlabeled datasets to improve diagnostic performance under real-world conditions. In Fig. 14b, Xu et al. reported an AI-enhanced electronic skin that simultaneously monitors vital signs and sweat chemistry, including pulse waveform, galvanic skin response, body temperature, and multiple metabolites and ions [201]. A machine learning pipeline then successfully differentiates various external stressors and quantifies the resulting stress responses during extended wear. Similarly, as illustrated in Fig. 14c, Kaveh et al. introduced wireless EEG earpieces for neurological monitoring. By utilizing dry electrodes and compact classifiers, the system infers human alertness directly from subtle neural signatures. This approach provides a highly practical solution for drowsiness monitoring, effectively bypassing the requirement for conventional laboratory setups that rely on cumbersome wet electrodes [193].

#### Non-Invasive Biochemical Sensing of Sweat and Interstitial Fluids/RNA

Noninvasive biochemical sensing utilizing sweat and interstitial fluid is rapidly expanding the scope of soft wearables from basic biophysical monitoring directly to advanced molecular diagnostics. Compared with electrophysiological signals, molecular readouts are inherently highly dimensional. They encompass multi-analyte panels, complex spectra, images, and reaction kinetics. Furthermore, they are highly susceptible to confounding variables, such as sweat rate, ambient temperature, skin contamination, and time-varying transport dynamics through microfluidics and membranes. Consequently, artificial intelligence and machine learning have become indispensable enablers. They extract quantitative concentrations from optical and electrochemical readouts, precisely correct for substantial sampling variability, and map complex multi-marker patterns directly to clinically relevant phenotypes. Notable examples include skin-interfaced microfluidics coupled with machine-learning-enabled image processing for quantifying sweat biomarkers in remote settings [202]. Similarly, surface-enhanced Raman scattering (SERS) combined with artificial intelligence enables sophisticated diagnostic classification for gout-relevant signatures [203]. In parallel, continuous wearable immunoassays tracking specific targets, such as C-peptide, highlight exactly how algorithmic inference is necessary to stabilize biochemical tracking over extended durations and personalize physiological baselines [194, 204].

As presented in Fig. 14d, Angenent-Mari et al. introduced a distinctive and increasingly significant direction in programmable diagnostics. Their approach treats RNA not merely as an analyte, but as an information-processing substrate, enabling sophisticated molecular-level computation for diagnostic purposes [34]. Deep learning maps relationships between physical sequences and biological functions for engineered RNA regulatory elements. This enables a significantly more reliable and scalable design of switches that activate specifically in the presence of a target trigger RNA. Conceptually, this RNA switch design provides a clear path toward compact and highly selective recognition modules. These modules could be seamlessly integrated with wearable sweat or ISF sampling systems and directly transduced into optical or electrical outputs. Approaches that optimize diagnostic sensitivity and specificity through the intelligent design of detection chemistries [205] can be directly paired with soft microfluidic sampling and on-body readout. This synergy creates comprehensive, nucleic-acid-aware wearable diagnostics.

This biochemical layer marks a critical transition from basic physiological monitoring directly to molecular-science-grounded health intelligence. It significantly strengthens digital biomarker profiling and anomaly detection by adding crucial mechanistic specificity. Furthermore, it supplies highly actionable molecular states that can ultimately drive precise closed-loop clinical interventions. By seamlessly integrating soft sampling hardware with artificial intelligence for both decoding measurements and designing recognition chemistry, this paradigm frames exactly how the next generation of wearables can move toward truly personalized, context-aware diagnostics far beyond the traditional clinic [194].

#### Intelligent Feedback Control for Autonomous Drug Delivery

Intelligent feedback control for autonomous drug delivery represents the ultimate therapeutic endpoint for AI-integrated soft bioelectronics. Continuous sensing is coupled with real-time inference and a control policy that actively modulates the specific dose, timing, and modality under explicit safety constraints. Unlike open-loop schedules, closed-loop therapeutics can seamlessly adapt to rapid physiological changes, inherent inter-patient variability, and prolonged drift. They achieve this sophisticated adaptation by combining state estimation derived from biochemical, biophysical, or imaging readouts with predictive models and highly adaptive control algorithms. Wearable systems for managing diabetes increasingly embed edge machine learning to ensure significantly safer insulin delivery [206]. Similarly, sense-and-treat cardiovascular patches illustrate highly adaptive interventions based on continuous blood pressure monitoring [207]. Related feedback paradigms extend far beyond traditional drugs to encompass electroceuticals and assistive physical actuation. These include intrinsically-controlled functional electrical stimulation for stroke rehabilitation [208] alongside machine-learning-driven personalized wearable robotic assistance [209].

Li et al. introduced an adaptive bioelectronic platform for wound therapy that tightly integrates diagnostics, decision-making, and active treatment within a portable and wireless physical form [195] (Fig. 14e). The system combines a wearable wound-imaging module with an artificial intelligence physician. This module analyzes sequential images to accurately infer the specific healing stage and recommend an appropriate real-time intervention. Prescribed regimen updates dynamically as the microenvironment of the wound evolves, enabling complex combinations of bioelectronic stimulation and drug treatment that are highly healing-stage-specific. This direct pipeline from imaging to therapy perfectly exemplifies how soft and conformal hardware provides clinically meaningful feedback variables when paired with learned decision modules.

Ultimately, this paradigm represents the critical juncture where intelligent sensing directly becomes intelligent intervention. The true clinical value of multimodal wearables is ultimately measured by their capability to close the loop safely and entirely autonomously. This concept fundamentally unifies exceptionally diverse therapeutic applications, seamlessly connecting adaptive wound care, blood pressure regulation, and precise metabolic control with closed-loop rehabilitation-oriented physical actuation. Together with the intelligent formulation of extended delivery systems, these collective studies indicate a profound paradigm shift. They point clearly toward personalized and continuously-learning treatment systems operating precisely at the intersection of soft compliant materials, embedded machine intelligence, and strict clinical constraints.

### Intelligent Human–Machine Interfaces for Immersive Interaction

AI-integrated soft electronics enable immersive, natural interaction in fields such as extended reality (XR), teleoperation, and assistive communication. In these domains, systems must simultaneously deliver low latency, high comfort, and long-term durability for daily use. This application marks a fundamental shift from passive health monitoring to a bidirectional information channel, where soft systems both interpret user intent and provide meaningful sensory feedback during active motion. Advanced materials are essential to this process, as ultraconformal and breathable interfaces ensure stable sensing and actuation over time. Simultaneously, adaptive AI provides the intelligent inference required to generalize performance across different users and environments. Ultimately, immersive human–machine interfaces represent a comprehensive co-design challenge—integrating soft transducers, embedded computation, and personalized learning to establish the foundation for tactile intelligence and safe physical interaction [32, 33, 147, 196].

#### High-Precision Gesture Recognition via Sensor Fusion

High-precision gesture recognition is a cornerstone capability for immersive human–machine interfaces (HMIs). Control within virtual and augmented reality (VR/AR) demands decoding user intent with extremely low latency and high fidelity during natural motion, across diverse users, and throughout extended wear. In practice, relying on a single sensing modality proves inherently insufficient. Strain and pressure distributions capture hand poses effectively but frequently become ambiguous during physical object interactions. Inertial cues resolve dynamics seamlessly but inherently drift over time. Biopotentials, such as electromyograms (sEMG), encode motor intent directly but are highly dependent on specific users and precise sensor placement. Sensor fusion addresses these critical gaps by intimately combining heterogeneous channels—including soft stretchable sensors, inertial measurement units (IMUs), triboelectric signals, epidermal electrodes, and visual inputs—into a highly observable state space. Furthermore, biologically-inspired learning frameworks that seamlessly integrate visual and somatosensory streams illustrate how cross-domain information fusion significantly boosts generalization beyond single-sensor pipelines [196, 210].

**Fig. 15:**
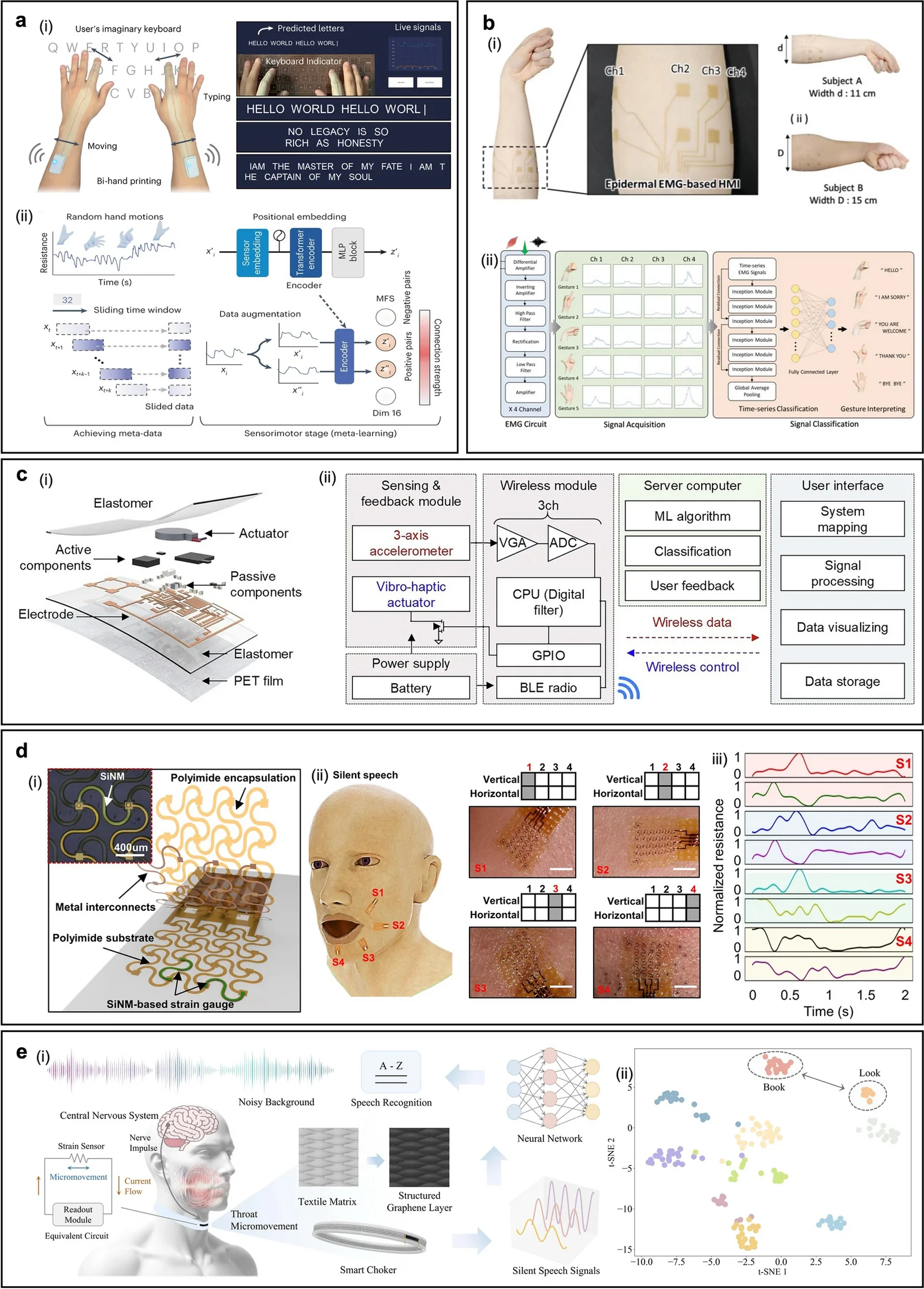
AI-integrated soft HMI platforms for immersive and bidirectional interaction. **a** Real-time two-handed typing recognition using nanomesh sensors, alongside the sensor signal-processing pipeline and unsupervised TD-C learning to construct the meta-feature space. Reproduced with permission [32]. Copyright 2023, Springer Nature. **b** An NW-DLF-printed epidermal EMG human–machine interface featuring its conformal forearm lamination, customized user layouts, and a block diagram of the associated hand-gesture recognition system. Reproduced with permission [211]. Copyright 2024, Wiley–VCH. **c** Exploded schematic of the elastomer-encapsulated device stack and electronic components, alongside a system-level block diagram detailing the main modules and wireless communication protocols. Reproduced with permission [33]. Copyright 2025, Springer Nature. **d** Ultrathin single-crystalline silicon nanomembrane stretchable strain gauge, illustrating a four-device facial mounting configuration alongside corresponding sensor deformations and time-series/heatmap resistance patterns during silent speech. Reproduced with permission [212]. Copyright 2022, Springer Nature. **e** A textile-graphene smart choker for silent speech recognition via neural-driven throat micromovements, featuring a lightweight decoding network and corresponding *t*-SNE visualizations of fine-tuned feature embeddings. Reproduced with permission [213]. Copyright 2024, Springer Nature

In Fig. 15a, Kim et al. introduced a substrate-free nanomesh receptor, demonstrating that highly conformal and mechanically unobtrusive epidermal interfaces can drastically stabilize signal acquisition by minimizing motion artifacts and skin-sensor impedance. Simultaneously, a meta-learning-based strategy rapidly adapts recognition models to entirely new tasks or users utilizing only minimal calibration data. This approach offers a highly attractive route when fusion-based models must maintain strict accuracy despite inevitable shifts in skin mechanics, physical placement, or individual behavioral styles [32]. In parallel, as presented in Fig. 15b, Yoon et al. demonstrated highly breathable and stretchable bioelectrodes utilizing metal nanowires on electrospun nanofiber membranes, emphasizing that fusion accuracy strictly begins at the initial sensory interface. Skin-compatible electrodes that rigorously minimize motion artifacts significantly improve the quality and comfort of long-duration biopotential recordings. This critical enhancement strengthens the reliability of intent-related features, which are subsequently fused with kinematic or tactile channels to decode gestures in extraordinarily fine detail [211].

This concept connects naturally to multimodal smart glove systems that combine complementary transducers to decode extraordinarily complex sets of gestures [147]. It also extends directly to machine-learning-powered smart textile gloves meticulously designed to capture precise hand motions and object interactions in everyday practical contexts [148]. Furthermore, fusing triboelectric and inertial data drastically expands systemic robustness during highly dynamic physical movements [214], whereas haptic feedback gloves effectively close the interaction loop by directly coupling precise gesture recognition with physical feedback to enhance embodiment within VR/AR [215]. By framing these exceptionally diverse systems under a unified perspective of sensory fusion, which potentially extends to computer-vision-assisted architectures, this paradigm clarifies the future trajectory. It demonstrates precisely how next-generation HMIs will achieve unprecedented precision and everyday practicality through the simultaneous optimization of soft hardware and multimodal intelligence.

#### AI-Enhanced Haptic Rendering and Texture Reproduction

AI-enhanced haptic rendering refers to the generation of machine-learning-driven actuator commands. These commands, encompassing vibrations, variations in pressure and shear, and electrical or thermal cues, actively elicit a desired tactile perception. Simultaneously, the reproduction of textures targets the complex spatiotemporal patterns encoding surface roughness, stick–slip friction behaviors, and transitions during physical contact. Within on-body soft devices, the mapping from the intended feeling to the exact physical actuation is strongly nonlinear and highly user- and device-dependent. Conditional generative models or optimization-based policies can efficiently search for waveforms that precisely match perceptual targets while strictly adhering to bandwidth and power constraints. Capturing dense tactile data, whether through biologically-inspired scanners or stretchable arrays of tactile sensors, provides the crucial training data required to learn highly compact representations of touch events.

As shown in Fig. 15c, Park et al. introduced a wearable interactive network for deep-learning-enabled full-body motion tracking and haptic feedback [33]. This research intimately couples distributed wearable sensing with networked haptic modules. It utilizes deep models to seamlessly infer full-body motion states during natural and completely unconstrained movement. The learned estimation of these physical states subsequently conditions closed-loop feedback. This mechanism allows haptic cues to be precisely timed and actively modulated alongside the dynamics of multiple joints, providing highly interactive guidance and embodied physical communication. This identical philosophy of sensing, inferring, and rendering underlies advanced smart glove systems designed for immersive VR/AR. In these platforms, multimodal sensing and precise haptic actuation are designed simultaneously to dramatically improve physical realism and overall usability [147, 215].

This capability proves absolutely pivotal because it moves fundamentally beyond merely recognizing user intent. Instead, it establishes truly bidirectional interfaces that actively shape human perception. While prior modules focus on extracting commands from the user, this rendering technology delivers highly informative and deeply personalized sensory feedback. Such targeted feedback can significantly stabilize human performance, dramatically reduce cognitive load, and enable a remarkably more natural sense of telepresence. This paradigm combines seamlessly with exceptionally high-fidelity tactile sensing designed for forceful and deformable physical interactions. It integrates beautifully with scanning-inspired tactile coding mechanisms, alongside machine-learning-empowered smart gloves to recognize diverse physical objects. Consequently, AI-enhanced rendering provides the definitive output channel. It entirely transforms soft electronics into closed-loop communication systems that remain profoundly perception-aware, driving the future of immersive VR and negligible-latency interactions.

#### Silent Speech Interfaces and Latency-Free VR/AR Interaction

Silent speech interfaces (SSIs) aim to decode linguistic intent directly from articulatory biological signals, such as subtle skin deformation and the precise motion of facial and neck muscles, without relying on airborne acoustics. Consequently, these interfaces enable privacy-preserving communication and highly robust interactions in noisy environments. Simultaneously, they offer a vital assistive pathway for users experiencing vocal impairments. The core technical challenge lies in the fact that these signals inherently possess very low amplitude, depend heavily on individual users, and are strongly affected by physical placement, sweat, and natural bodily motion. Therefore, exceptionally high-fidelity soft sensors must be paired directly with data-driven decoding models. These models learn complex nonlinear spatiotemporal patterns and successfully generalize across entirely different usage sessions. This paradigm aligns perfectly with VR/AR interactions requiring absolutely negligible latency. In these highly immersive environments, continuous intent decoding and speech-like hands-free commands seamlessly complement contactless spatial tracking and persistent wearable interfaces.

In Fig. 15d, Kim et al. demonstrated that ultrathin crystalline silicon strain gauges leverage the exceptional piezoresistive response of single-crystal materials while maintaining conformal mechanics completely compatible with human skin [212]. The resulting strain readouts exhibit a remarkably high signal-to-noise ratio (SNR). This quality perfectly matches deep neural decoders specifically tasked with mapping multichannel physical dynamics directly to discrete units of speech. In this architecture, the physical sensor provides completely stable mechanical transduction with exceptionally high resolution. Concurrently, the neural network effectively absorbs residual nonideal characteristics, including inter-subject variability, highly nonlinear skin mechanics, and the simultaneous activation of adjacent muscles, elegantly transforming them into entirely learnable representations. As illustrated in Fig. 15e, Tang et al. introduced ultrasensitive textile strain sensors, strongly emphasizing everyday wearability and systemic scalability [213]. This approach translates these interface concepts directly into highly breathable, garment-like physical forms. Simultaneously, it targets exceptionally high machine-learning efficiency, achieving remarkably strong decoding performance utilizing limited training data and lightweight inference operations.

This evolution enables significantly more natural and continuous interfaces strictly essential for both immersive reality and vital assistive technologies. This ambitious trajectory is strongly reinforced by machine-learning-assisted, tattoo-like ultrathin electronics completely designed for recognizing silent speech across all environmental conditions [216]. Furthermore, it is deeply supported by the precise decoding of directional facial movements utilizing advanced strain sensors [217] and highly efficient lip-language interpretation systems that explicitly reconstruct physical motions related to human speech [218]. These advanced capabilities can be seamlessly combined with user-generic ultrasound tracking systems for precise hand motion alongside highly scalable soft electrode arrays supporting persistent AR-enabled interfaces [219]. Consequently, these developments point decisively toward continuously active multimodal interaction stacks. Within these advanced systemic stacks, soft physical hardware and artificial intelligence jointly deliver unprecedented robustness, profound personalization, and truly imperceptible latency.

#### From Human–Machine Interfaces to Human–Machine–Environment Interaction and Embodied Intelligence

Beyond decoding user intent, next-generation wearable soft electronics are increasingly expected to perceive the surrounding environment and to use those exteroceptive signals as part of decision-making. This shift is closely related to embodied intelligence, in which sensing, body mechanics, and control are co-designed so that meaningful perception emerges from physical interaction rather than isolated signal acquisition alone. Recent studies already illustrate this transition. Wang et al. developed a reconfigurable omnidirectional triboelectric whisker sensor array for human–machine–environment interaction that can be reversibly deployed on diverse surfaces and resolve omnidirectional force and motion [65]. Zhou et al. reported an artificial epidermis capable of independently sensing NO_2_ gas and pressure, showing that wearable interfaces can jointly monitor the user state and external environmental exposure [220]. At the interface level, He et al. further demonstrated a fully biomimetic flexible sensor that switches seamlessly between contact and noncontact modes, supporting multimodal interaction beyond direct touch [61, 65].

This exteroceptive capability provides a natural bridge to tactile intelligence in soft robotics and e-skins. Once a soft interface no longer treats touch as a command input alone but as structured information about surfaces, objects, and interaction dynamics, the same sensing stack becomes relevant to robotic perception and adaptive control. Luo et al. showed that conformal tactile textiles can record and learn human–environment interactions across the body [221], and Jiang et al. demonstrated that a 1152-channel stretchable tactile glove coupled with visual–tactile learning can reconstruct dynamic hand–object states during manipulation. Extending this concept beyond wearable interfaces [150], Li et al. integrated multimodal sensing, actuation, and onboard decision-making in flexible electronic robots, explicitly framing environmental exteroception as part of embodied artificial intelligence [197]. Collectively, these examples clarify how wearable human–machine interfaces can evolve toward embodied systems that jointly encode self-motion, contact, and environmental context, thereby motivating the broader discussion of tactile intelligence in Sect. 7.3.

### Tactile Intelligence in Soft Robotics and E-Skins

Tactile intelligence is the critical “missing sense” that allows soft machines to operate autonomously, marking a transformative shift from traditional rigid robotics. While soft robots and electronic skins excel at safe and conformal contact, their inherent flexibility produces complex, nonlinear signals that traditional analytic models struggle to interpret. Artificial intelligence addresses this challenge by converting large-area tactile data into actionable perception, enabling systems to feel, understand, and respond during real-world interaction. This progress relies on merging functional soft materials with robust signal compensation and multimodal learning to manage environmental variability. To meet the strict power and latency demands of embodied agents, energy-efficient local intelligence is also becoming essential. Ultimately, tactile intelligence acts as a system-level proving ground where the co-design of hardware and algorithms is the vital pathway to achieving truly dexterous, safe, and adaptive soft robotics [145, 198, 222–225].

#### Deep Learning-Based Proprioception and Shape Estimation

Deep-learning-based proprioception and shape estimation address a fundamental mismatch between the physical mechanics of soft bodies and traditional robotic perception. Unlike rigid links featuring low-dimensional kinematics, soft robots and electronic skins deform continuously. This continuous physical deformation produces complex responses that are highly dimensional, inherently nonlinear, and heavily history-dependent. It requires accurately estimating internal posture, physical curvature, distributed physical strain, or fully three-dimensional (3D) surface morphology directly from sparse or widely distributed sensor readouts. These readouts typically emerge from resistive and capacitive arrays, optoelectronic foams, and electrical impedance tomography (EIT). Fully data-driven computational models prove particularly effective for addressing this exact challenge. Convolutional neural networks (CNNs) analyze complex spatial fields, recurrent neural networks (RNNs) ensure strict temporal continuity, and Transformer-based architectures excel at learning extended sequences. Furthermore, they naturally integrate crucial cues from entirely different modalities while actively compensating for systemic physical drift, mechanical hysteresis, and unpredictable contact-induced perturbations.

**Fig. 16:**
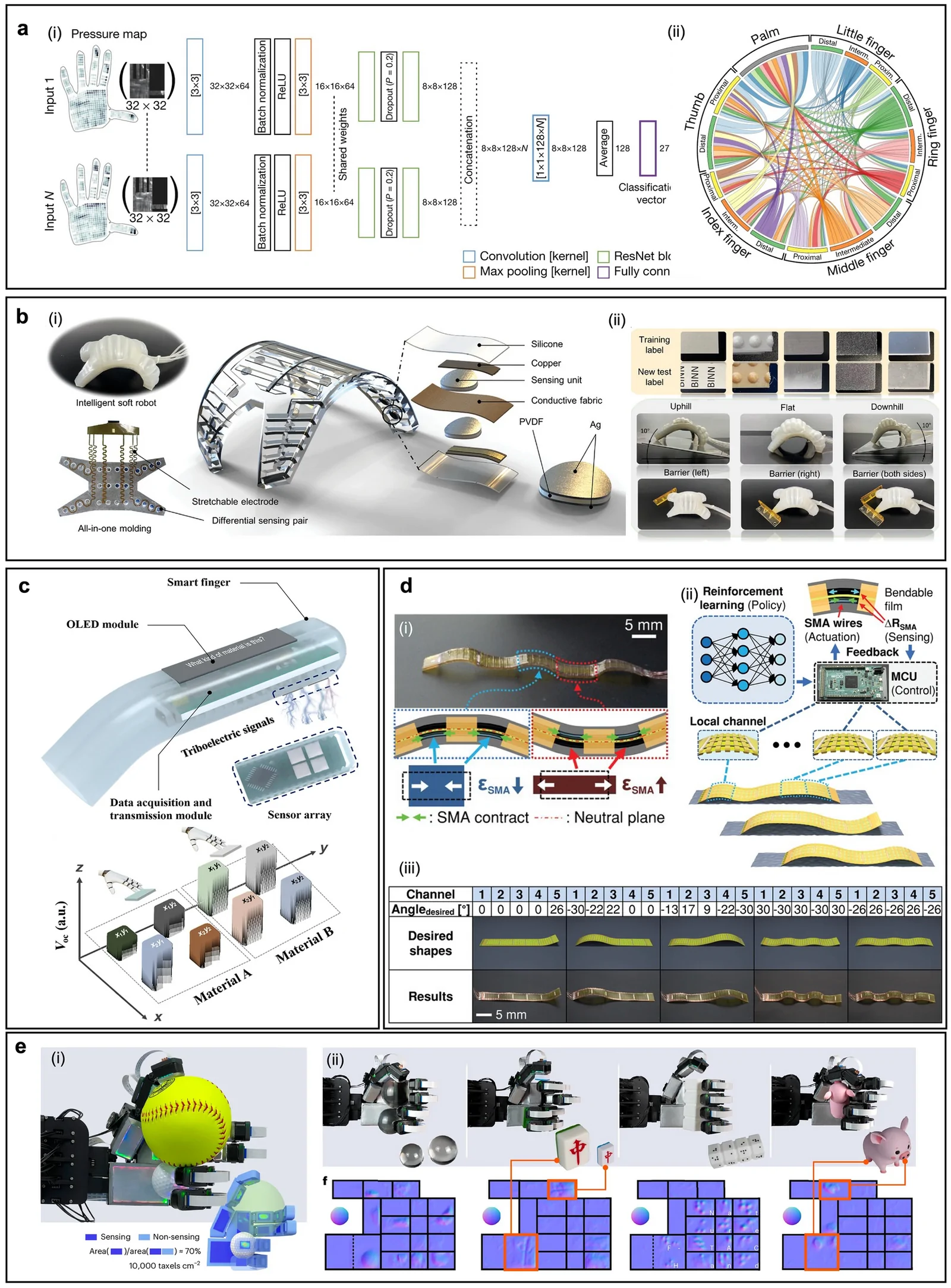
Tactile intelligence in soft robotics for autonomous sensing and interaction. **a** CNN architecture for tactile glove-based object recognition and corresponding hand-region co-activation maps during forceful gripping. Reproduced with permission [226]. Copyright 2019, Springer Nature. **b** Schematic of a soft robot integrated with an SSES-based electronic skin sensing interface, alongside a demonstration of model generalization in recognizing five previously unseen terrains. Reproduced with permission [222]. Copyright 2023, Wiley–VCH. **c** Architecture of the triboelectric tactile smart finger and its conceptual output waveforms for distinguishing contacted materials. Reproduced with permission [227]. Copyright 2022, American Association for the Advancement of Science (AAAS). **d** Design and actuation of a multichannel shape-memory alloy-driven soft robotic flatworm, alongside an RL-based closed-loop control. Reproduced with permission [225]. Copyright 2023, Wiley–VCH. **e** F-TAC Hand, a dexterous robotic hand equipped with a physiologically benchmarked, high-density tactile sensor array that records high-resolution contact patterns during grasping to enable adaptive, human-like manipulation. Reproduced with permission [228]. Copyright 2025, Springer Nature

In Fig. 16a, Sundaram et al. demonstrated that exceptionally dense, body-conformal tactile distributions can be effectively treated as learnable signatures defining complex hand poses and physical contact states. High-resolution machine learning models trained directly on these pressure patterns can successfully decode intricate grasp configurations and the broader context of physical interactions. This innovative approach provides a robust systemic blueprint for mapping distributed tactile data to latent kinematic and morphological variables entirely in real time [226]. Complementarily, as shown in Fig. 16b, Shu et al. introduced an advanced framework explicitly targeting the critical dual requirements defining modern soft robots. It focuses intensely on estimating internal self-deformation for precise proprioception while simultaneously remaining highly sensitive to external physical touch. By pairing e-skin-like sensing elements with data-driven inference, this research highlights how complex neural models can successfully disentangle tightly coupled mechanical responses.

This advanced perceptual backbone establishes the critical foundation necessary for achieving advanced tactile intelligence across soft robotics and sophisticated electronic skins. For instance, the successful rejection of physical disturbances in aquatic soft wings deliberately leverages embedded sensors for closed-loop control [229]. Simultaneously, deep-learning-powered highly stretchable tactile arrays successfully capture exceptionally forceful interactions with completely deformable objects [150]. Other significant architectural advances include EIT-based ultrathin skins for fully 3D tactile interfaces, Transformer-based models reconstructing physical morphologies with extraordinary resolution, and RNN-based perception demonstrating temporally coherent estimations of highly dynamic physical states [137, 145]. Together, these collective advancements forge a fundamentally critical and unbreakable link. They seamlessly bridge the profound gap between soft compliant materials capable of physical feeling and fully intelligent robotic systems that comprehensively understand their own physical shape.

#### Texture Classification and Slip Detection for Adaptive Grasping

Texture classification and slip detection represent cornerstone capabilities for adaptive grasping within soft robotics and advanced electronic skins. They translate raw contact signals directly into actionable cues regarding surface microstructure, complex frictional regimes, and the overall stability of the physical grip. Unlike static force estimation, these critical tasks depend entirely on rapid spatiotemporal dynamics. These dynamics include microscopic vibrations during active scanning, stick–slip transitions, and shear-induced rapid signal changes that immediately precede macroscopic mechanical failure. Accordingly, recent progress seamlessly couples exceptionally high-bandwidth tactile hardware with advanced machine-learning-based decoders. High-spatiotemporal-resolution tactile systems consistently demonstrate fine texture recognition under carefully controlled exploratory motions [223]. Simultaneously, bimodal sensors jointly measuring physical softness and surface texture provide significantly richer prior information for complex manipulation within entirely unstructured environments [230].

At the perceptual level, fusing multiple modalities, such as combining tactile feedback directly with computer vision, drastically improves generalization whenever isolated tactile cues remain ambiguous. This intelligent fusion strongly supports highly dexterous manipulation during everyday household tasks [231]. Another rapidly emerging direction involves real-time algorithmic signal unmixing. Here, active-learning-assisted optical tactile sensing precisely decouples highly dynamic touch components, successfully stabilizing downstream classification and precise slip inference [232]. This approach effectively transforms highly compliant graphene skins into exceptionally reliable modules for tactile perception [233].

As illustrated in Fig. 16c, Qu et al. demonstrated a triboelectric-sensing-based artificial smart finger designed specifically for identifying materials [227]. This innovative work leverages the fundamental triboelectric effect, specifically contact electrification and electrostatic induction, to generate highly distinctive electrical signatures completely without external power during physical touch and active sliding. Because these complex triboelectric waveforms remain extraordinarily sensitive to interfacial charge transfer, the microscopic evolution of contact, and intricate frictional interactions, they naturally encode highly discriminative features strictly essential for precise material identification. The key contribution of this research lies in demonstrating that material-aware tactile perception can be achieved utilizing exceptionally compact hardware paired directly with fully data-driven recognition algorithms.

Focusing on advanced tactile intelligence establishes the foundational enabling layer for fully autonomous, exceptionally safe, and highly capable soft robots. This specific capability proves absolutely pivotal because it directly targets decision-critical sensory primitives, namely surface texture and physical slip, which comprehensively govern grasp stability and dexterous physical manipulation. This paradigm beautifully bridges remarkably diverse sensing modalities, spanning triboelectric systems, optical arrays, and advanced graphene skins [227, 232, 233], directly with highly scalable strategies transferring learned knowledge across sensors [234] and contextual fusion spanning multiple modalities [231]. Together with biologically-inspired signal pathways, such as artificial organic afferent nerves explicitly designed for closed-loop tactile feedback [224], these collective advancements establish the ultimate foundation for highly complex interaction policies. Within advanced policies trained through reinforcement learning, the exceedingly reliable perception of slip and texture firmly becomes the indispensable feedback substrate for highly adaptive and entirely autonomous robotic control.

#### Reinforcement Learning for Collision Detection and Safe Interaction

Reinforcement learning (RL) provides a natural framework for collision-aware control and safe physical interactions within soft robotics. In these systems, the physical body deforms continuously, making analytical modeling of contact dynamics exceedingly difficult. The fundamental concept involves closing the operational loop connecting distributed soft sensing—encompassing both proprioception and touch—with the learning of active policies. This integration empowers robots and electronic skins to detect incipient collisions or unexpected physical contacts, infer the location and force of these contacts from high-dimensional signals, and generate highly adaptive actions that perfectly balance task success with physical safety. Because soft robots operate under partial observability and nonideal sensor characteristics, RL policies are frequently paired with learned state estimators, such as recurrent encoders. Furthermore, they are rigorously trained utilizing safety-aware objectives, incorporating contact penalties, constraint-based rewards, or sophisticated shielding mechanisms.

In Fig. 16d, Ju et al. presented a closed-loop control strategy for soft robots that utilizes coordinated reinforcement learning policies combined with internal proprioceptive sensing [225]. This architecture explicitly treats embedded internal sensing as the fundamental state backbone for learning highly contact-resilient behaviors. By leveraging proprioceptive feedback to accurately infer physical configurations and external perturbations, the learning algorithm establishes coordinated actuation strategies. Complementarily, as shown in Fig. 16e, Zhao et al. demonstrated that embedding extremely high-resolution touch across robotic hands transforms physical manipulation [228]. Comprehensive touch maps provide immediate critical cues for the onset of contact, the distribution of physical pressure, and precursors to physical slip. This enables highly adaptive grasp regulation that perfectly mimics human dexterity while remaining inherently safer. Together, these advancements emphasize that success relies not merely on isolated learning algorithms. Instead, reinforcement learning becomes exponentially more powerful when empowered directly by exceptionally high-bandwidth soft sensing, effectively transforming collision events directly into highly actionable learning signals.

This integration represents the absolute apex of decision-making and control. After successfully learning internal shape and complex tactile semantics, the intelligent system must autonomously act with absolute safety entirely in real time. Deep reinforcement learning tailored for actuating magnetic soft robots [235] alongside the successful transfer of models from simulation to reality for multimodal locomotion [236] profoundly highlight how learned policies flawlessly adapt across highly dynamic physical regimes. Concurrently, recurrent-model-based embedded sensing [137] and the concept of expected sensing for jointly detecting proprioception and physical contact [237] provide the indispensable perceptual substrates rendering safe reinforcement learning entirely feasible.

Taken together, the application examples in this section show that the value of AI-integrated soft electronics lies not in isolated model accuracy, but in the coordinated performance of materials, interfaces, computation, and control during real use. Personalized healthcare, immersive HMIs, and tactilely intelligent soft robots all depend on the same system-level requirements: stable long-term interfacing, efficient local or edge intelligence, robust generalization across users and contexts, and clear metrics for safety, reliability, and autonomy. These cross-cutting lessons frame the concluding perspective in Sect. 8, where the remaining bottlenecks and future opportunities for deployable intelligent soft systems are synthesized.

Table 2 provides a comprehensive overview of how artificial intelligence is integrated into soft and flexible wearable electronics. It systematically categorizes the specialized materials used for smart devices alongside the specific AI models employed to analyze complex biometric data.

**Table 2:** Materials and AI models for AI-integrated soft and wearable electronics

Category	Materials	DL model/AI computation	Application	References
Signal enhancement and compensation	Screen printed silver (Ag) electrode	2D CNN denoising auto-encoders	Textile ECG noise/motion artifact removal	[19]
	Hydrogel electrode + AgNW	CNN feature extractor + FC	Rehab motion activity recognition	[24]
	PVC/nylon + Cu	Dilated RNN + prototype learning	Lip language decoding	[135]
	Ecoflex + Ag electrode array	CNN autoencoder + AdaBoost	Cuffless blood pressure estimation	[143]
Neural architectures for multimodal wearable data	Screen printed silver (Ag) + dielectric ink + conductive hydrogel	Hyperdimensional computing (HDC)	Hand gesture recognition	[144]
	Polyurethane + Electrospun PAN + Gold (Au)	Bi-LSTM RNN + FC	Hand motion + object recognition	[148]
	Ecoflex + Carbon black (CB) + EGaIn	Transformer	Morphology reconstruction	[145]
	PI (Polyimide) + Ti + Cu	Temporal CNN/GLU + GNN	Gesture recognition	[140]
	microstructured elastomer; flexible substrate	Spike coding + MLP/kNN	Texture classification	[155]
Bio-Inspired synaptic devices and material intelligence	PEDOT:PSS OECT + Au	On-site analog amplification	Electrochemical aptamer-based biosensing	[168]
	Ag₂S + Ag atomic bridge	In-device Filament/ionic dynamics	Hardware synapse STP/LTP emulation	[169]
Architectures for in-sensor and near-sensor computing	PEDOT:PSS + SEBS + Au + ion-gel + AgNW	Reservoir computing + FC	Biosignal edge computing + EMG	[166]
	BT NP / P(VDF-TrFE) + pentacene + PI	Synaptic transistor	Tactile sensing/memory/filter	[181]
Personalized healthcare and closed-loop therapeutics	PI/PDMS/PET + Ag/C	XGBoost (cls/reg) + SHAP	Stressor type/anxiety score	[201]
	PDMS + Ag/AgCl	Autoencoder + LQR + DRL (actor-critic)	Wound stage + adaptive EF/drug	[195]

*FC* Fully Connected layer, *SHAP* SHapley Additive exPlanations, *LQR* Linear Quadratic Regulator, *DRL* Deep Reinforcement Learning, *CNN* Convolutional Neural Network, *RNN* Recurrent Neural Network, *LSTM* Long Short-Term Memory, *GNN* Graph Neural Network

Table 3 complements this model-centric landscape by comparing application-level maturity indicators, including task-level AI performance, interface quality, system robustness, operational autonomy, and current limitations, across representative healthcare, HMI, and soft robotic systems.

**Table 3:** Application-level evaluation metrics and current maturity indicators for representative AI-integrated soft electronic systems

Application	AI metric	Comfort/interface	Stability/duration	Current stage/limitation	References
Anomaly detection (dry ear-EEG)	93.2–93.3% drowsiness accuracy	User-generic dry in-ear electrodes; compact wireless form factor	35 h dataset across 9 subjects	Population-trained neural monitoring is feasible, but validation remains task-specific	[193]
Biomarker profiling (reusable hydrogel EEG)	91.38% attention classification	310 ohms impedance; reusable adhesion 104 kPa (30 cycles)	20,000 cycle stability, 14-day signal fidelity 25.2 dB	Strong interface maturity, but cohort and task remain limited	[90]
Biochemical sensing (multiplex microneedle eMPatch)	0.996 classification, *R*^2^ = 0.977	30 min skin recovery; no visible irritation; no erythema/inflammation after 7 d	> 88% retention over 120 min, RSD < 2.64%, stable under bending/twisting	Mechanically robust, but evidence is still largely preclinical	[194]
Closed-loop therapy (a-Heal wound platform)	ML-guided stage diagnosis + adaptive EF/drug control	Portable, wireless, bandage-integrated wearable	Imaging + therapy updated every 2 h over day 0–7	Proof-of-concept closed loop; longer protocols limited by device failure/external power	[195]
Immersive HMI (motion tracking + haptics)	~ 97% classification, AUPRC 0.994, ~ 40 ms inference	No irritation after 24 h; no redness/itching/discomfort	24.5–27 °C epidermal temperature, ~ 160 h transmission	Strong wearability benchmark, but orientation/placement changes still matter	[33]
Interactive HMI (organogel pressure HMI)	98% alphabet recognition	Self-adhesive, biocompatible organogel	Stable for 1 month, 40–95% RH, − 20 to 45 °C	Promising robust HMI, but task complexity remains limited	[196]
Embodied soft robot (AI-embodied FEbot)	Onboard self-learning/hyperdimensional computing	Multimodal proprioception + exteroception	87.6 mm/s vertical climbing, 5.1 × payload, survives 500 kg compression	Embodied intelligence is advancing, but standardized long-duration autonomy metrics are still lacking	[197]

For wearable healthcare/HMI rows, comfort/interface emphasizes conformability, irritation, thermal burden, low impedance, or minimally invasive coupling. For soft robots, the analogous maturity indicators are mechanical robustness, perturbation tolerance, payload, and sustained closed-loop operation

*AUPRC* Area Under the Precision-Recall Curve, *RSD* Relative Standard Deviation, *RH* Relative Humidity, *EF* Electric Field

## Conclusion and Outlook

This review has summarized the recent advances driving the convergence of soft electronics and artificial intelligence, spanning from materials to intelligent systems. Deployable performance is shaped by the coupled behavior across physical transduction, integration density, and learning pipelines under realistic wear, rather than by isolated improvements in any single layer. Stable, low-noise coupling to the body and deformation-tolerant conduction remain prerequisites for extracting meaningful information. Simultaneously, scalable integration and interconnect architectures define what can be measured, how densely it can be sampled, and how reliably it can be routed, thereby shaping the data streams that learning systems must interpret.

Recent progress in soft materials and interfaces has broadened the design space beyond filler-based composite percolation concepts to include intrinsically stretchable conductors, conducting polymers, and platforms that support conformal epidermal coupling and low-impedance interfacing. In long-duration wear, comfort is inseparable from signal quality. Breathable substrates and skin-compatible interfaces influence moisture management and contact stability, and these effects propagate directly to drift, noise, and artifact susceptibility at the inference input.

As systems expand from individual sensors toward wearable platforms, the importance of integration and manufacturability becomes central to system design. Scalable fabrication and multilayer interconnect strategies enable higher channel counts and multifunctional stacks, but they also increase the dimensionality and variability of the acquired data streams. As multimodal and arrayed configurations become more common, data interpretation becomes a primary design constraint, given that on-body signal statistics shift with placement, motion, and environment. Battery-free and energy-harvesting approaches can extend operation time, but they tie duty cycle, sensing modality, and compute budget together, reinforcing the value of co-design between data acquisition and on-device processing.

To bridge the gap between laboratory prototypes and commercially viable intelligent soft systems, the field requires tighter coupling between scalable manufacturing, standardized validation, and drift-tolerant artificial intelligence. Future progress depends not only on producing larger or denser devices, but also on ensuring that batch-level reproducibility, longitudinal stability, and cross-user robustness are quantified with shared benchmarks. Particularly for wearable systems intended for prolonged real-world operation, standardized reporting of fabrication windows, device-to-device variation, aging behavior, recalibration frequency, and out-of-distribution performance will be essential for meaningful comparison across studies. Commercial translation will therefore be accelerated not by hardware innovation or algorithm development alone, but by a co-design framework in which fabrication control, reliability testing, and adaptive learning are developed as a single pathway toward reproducible and transferable intelligent soft electronics.

Learning pipelines are used to stabilize information extraction through artifact suppression, compensation of nonideal responses, and mitigation of session-to-session drift, while remaining efficient enough for edge execution. As platforms expand to multimodal and arrayed configurations, algorithms must exploit spatiotemporal structure and modality complementarity, yet remain robust to missing channels, variable contact conditions, and user-to-user differences. Deployment constraints will further push toward tighter integration between acquisition and inference, including selective sampling, on-device feature extraction, and adaptive inference that reduces reliance on continuous high-bandwidth transmission.

A key opportunity lies in the tighter coupling between soft hardware and learning. Approaches that reduce data movement and shorten the sensing-to-decision loop, including neuromorphic and in-sensor computing concepts, can better match dense soft sensor networks to edge constraints. In practice, a central benchmark is whether AI pipelines can deliver reliable, generalizable inference from imperfect and time-varying soft signals during everyday wear, enabling intelligent soft systems that operate continuously and with consistent performance outside laboratory conditions. Equally, performance claims should be judged by how gracefully inference degrades under motion artifacts, partial detachment, and environmental shifts, rather than by curated laboratory accuracy alone.

While complex multimodal models enhance system robustness, they often exceed the stringent power, memory, and thermal limits of skin-interfaced platforms. Furthermore, long-term environmental exposure and mechanical wear inevitably cause sensor drift, meaning practical deployment requires continuous, drift-aware calibration strategies rather than static, one-time setups.

As laboratory prototypes transition to manufacturing scales, batch-to-batch variations further complicate AI generalization. Consequently, mass-production reliability and AI robustness must be validated concurrently, highlighting an urgent need for shared, standardized benchmarks regarding wear duration, power consumption, and cross-batch consistency. Moving forward, isolated advancements in softer devices or more accurate models will no longer be sufficient. Instead, progress relies on platform-level co-optimization that seamlessly integrates materials, edge hardware, and adaptive algorithms. Ultimately, the field must shift its evaluation priorities from peak laboratory accuracy toward durable autonomy, manufacturable reliability, calibration-light operation, and interoperable system standards.
